# Magnetoencephalography reveals differences in brain activations for fast and slow responses to simple multiplications

**DOI:** 10.1038/s41598-021-97927-8

**Published:** 2021-10-13

**Authors:** Giorgio Arcara, Rachele Pezzetta, S. Benavides-Varela, G. Rizzi, S. Formica, C. Turco, F. Piccione, C. Semenza

**Affiliations:** 1 IRCCS San Camillo Hospital, Via Alberoni 70, Lido, 30126 Venice, Italy; 2 Department of Developmental Psychology and Socialization, University of Padova, Padua, Italy; 3 Department of Neuroscience (Padova Neuroscience Centre), University of Padova, Padua, Italy; 4 Department of Experimental Psychology, Ghent University, Ghent, Belgium; 5 Riabilitazione, Azienda Ospedale - Università di Padova, Regione Veneto, Italy

**Keywords:** Neuroscience, Psychology

## Abstract

Despite decades of studies, it is still an open question on how and where simple multiplications are solved by the brain. This fragmented picture is mostly related to the different tasks employed. While in neuropsychological studies patients are asked to perform and report simple oral calculations, neuroimaging and neurophysiological studies often use verification tasks, in which the result is shown, and the participant must verify the correctness. This MEG study aims to unify the sources of evidence, investigating how brain activation unfolds in time using a single-digit multiplication production task. We compared the participants' brain activity—focusing on the parietal lobes—based on response efficiency, dividing their responses in fast and slow. Results showed higher activation for fast, as compared to slow, responses in the left angular gyrus starting after the first operand, and in the right supramarginal gyrus only after the second operand. A whole-brain analysis showed that fast responses had higher activation in the right dorsolateral prefrontal cortex. We show a timing difference of both hemispheres during simple multiplications. Results suggest that while the left parietal lobe may allow an initial retrieval of several possible solutions, the right one may be engaged later, helping to identify the solution based on magnitude checking.

## Introduction

Several sources of evidence seem to suggest that a simple arithmetic task—as one-digit multiplication—entails a relatively complex process, bilaterally located in the brain^1–3^. This is in contrast with traditional explanations of multiplication deficits in the neuropsychological literature. Dehaene and Cohen’s review^4^ of traditional accounts concluded that only the left hemisphere, in the lower parietal lobe, stores a repertory of tables (i.e., over-learned single-digit multiplications (e.g.,7 × 3 = 21; 6 × 7 = 42), referred to, in math psychology, as "arithmetical facts"). A lesion to this system would lead to mistakes in the retrieval of such "facts". A closer inspection of neuropsychological evidence, however, shows that in mental multiplication there is also a crucial contribution by the right hemisphere, insofar as right brain-damaged patients commit a significant number of mistakes. This fact, however, was not noticed and commented upon before recent studies^5^.

Nowadays, the notion that both hemispheres are involved in simple multiplication is supported by evidence from different techniques (fMRI^1,6^, TMS^7^, EEG^8^, MEG^3^, and PET^9^). An interesting hint on the relative role of the left and right hemispheres (especially of parietal lobes) comes from studies with Direct Current Electrocortical stimulation (DCE) during neurosurgery^10,11^. Results in these studies showed errors associated with the inhibition of either left or right parietal sites. Crucially, the errors were qualitatively different according to the hemisphere that was stimulated. Inhibition of left sites, resulting in a greater role for the right hemisphere, was associated with a prevalence of approximation errors (e.g., 7 × 3 = 20). In contrast, inhibition of the right sites, with the left hemisphere taking a relatively greater burden, showed a prevalence of retrieval errors^10^, namely the solution was erroneously chosen from stored solutions (e.g., 7 × 3 = 28). Thus, each hemisphere would potentially contribute to obtaining the solution of the operation: the left hemisphere would act by retrieving a possible solution, and the right hemisphere by indicating the approximate numerical interval where such a solution should be sought. A particular strength of these latter results was related to the high spatial specificity of the technique employed (i.e., DCE), which allowed for direct interference with specific brain areas. However, only specific sites were stimulated, and it was not possible to investigate more complex networks or track the involvement over time of specific areas. The knowledge of the time-course of brain activation during simple mental multiplication and the lateralization of these processes mostly comes from electroencephalography (EEG) studies on healthy participants.

One of the first studies that investigated the time-course activity and hemispheric involvement during one-digit multiplication was performed with ERPs (event-related potentials), using a verification task (e.g., 6 × 4 = 24: “yes or no?”^8^). They found a distinction between simple and difficult problems—while the former involved a short-lived activation in the left parietal electrodes, the latter rose more slowly in the electrodes over the same areas. Additionally, with increasing processing times, effects were found on electrodes of both hemispheres. In another study, Jost and collaborators^12^ found that the effects depended on the problem size, with operations classified as "larger problems" (e.g., 7 × 8) showing larger slow negativity in frontocentral and right temporal electrodes, as compared to "smaller problems" (e.g., 2 × 3). While EEG studies on mental multiplication mostly employed ERP analyses, several studies on other aspects of mental arithmetic (i.e., addition or subtractions) exploited time–frequency analysis to investigate modulations on specific oscillatory responses^13–18^. Even if none of these time–frequency studies focused specifically on the lateralization of areas, results suggest that modulation in certain frequencies is associated with specific strategies, with theta increase associated with fact retrieval^14^ and alpha decrease associated with the use of procedural strategies^17,19^. Thus, the study of the oscillatory responses can provide useful complementary information on mental processing^20^. Importantly, even if many of these EEG studies explicitly suggested the potential involvement of either the left or the right hemisphere, these claims were based on results obtained at electrode level. Given the low spatial resolution of the EEG, any conclusion on the brain areas involved (without appropriate analysis, such as source estimation) may be potentially misleading^21^ and should be taken cautiously. This limitation has been recently overcome by Salillas et al.^3^ with magnetoencephalography (MEG), which warrants a very good spatial resolution when source estimation methods are used. In the study of Salillas et al.^3^, which also used a verification task, results confirmed the involvement of both hemispheres and at least three brain networks involved, which included the bilateral inferior frontal areas, mainly activated in responses to correct solutions, a left-lateralized frontoparietal network, which is activated in responses to incorrect table-related solutions, and a right-lateralized frontoparietal network, activated in responses to unrelated solutions.

Despite this vast literature, there are still many open questions on the relative role of specific brain areas during simple single-digit multiplications. One open question concerns the choice of the task design. While in neuropsychological^22^ and DCE^10,11^ studies, patients are asked to orally retrieve the result (in this way the experimental task corresponds closely to the behavior of interest), most of the evidence on the time-course of single-digit multiplication in neuroimaging (e.g., PET, fMRI) and neurophysiology (e.g., EEG, MEG) is based on verification tasks^3,8,23–28^. Indeed, the experimental design based on verification has some advantages, as it avoids the problem of oral response artifacts and allows for finer control of the experimental setting. However, verification is a less frequent task in everyday life. Moreover, it introduces some potential confounding effects. Besides encoding both operands, verification tasks could strongly induce plausibility checking strategies, also requiring the evaluation of the proposed answer and its mathematical relation with the operands. Instead of performing a calculation, a participant could wait for the proposed response, first use some plausibility checks, and then come up with a decision (correct or not). This is particularly relevant for incorrect responses. In theory, one could accurately classify an incorrect response (e.g., 5 × 4 = 21) as “wrong” without accessing the actual correct response, but just retrieving from memory general information that in single digit multiplication involving 5 and 21 is not a possible result. Although this potential problem has been taken into account by classifying incorrect responses between “table related” or “not table related”^23^, it is not possible to fully exclude that the participant relies on strategies to perform the task, rather than reflecting the investigated behavior of performing calculations. Other studies employed alternative methods to tackle these limitations, for example using a task in which several operations were concatenated and ERPs between the operations were analyzed (e.g., 2 × 3 + 7)^12^. In that task, participants must implicitly produce the interim result of the concatenated operation before they can go on with the next calculation step. Even if this task limits the possibility of plausibility checking, it still relies on verification and cannot completely rule out the possibility that approximate calculations were performed, and that the final answer was based on plausibility.

In the present study, we tried to overcome these limitations by designing a task that specifically tackles these issues and provides additional hints on how multiplication occurs in the brain and on how the involvement of specific areas (focusing on left and right parietal lobes) unfolds in time. To this aim, we used MEG, exploiting its excellent temporal and good spatial resolution. We used a simple production task, in which participants were asked to say aloud the response as fast as possible. This allows a direct comparison with the paradigms used in the neuropsychological and DCE literature on one side, and with neuroimaging and neurophysiological data obtained with healthy participants, on the other side. To compare response efficiency, we adopted an individual-based approach to classify the problems into two groups. In our case, efficient (i.e., correct and fast, *Fast Responses*) and inefficient (i.e., correct and slow, *Slow Responses*) responses were compared on an individual basis (for an analogous strategy, see for example^29–31^). This allowed us to compare not fast and slow responders, but fast and slow responses, within the same individual, comparing two activities in which the processing is likely to be very similar, but with different efficient outcomes. As in previous verification studies^3,14^, we focused our analysis on both source activity and time–frequency analyses. Based on previous clinical findings, with production tasks in neuropsychological populations and in DCE^10^, we focused on the left and right parietal lobes, hypothesizing a sequential activation of the left followed by activation of the right hemisphere, especially prominent in *Fast* compared to *Slow Responses*. We expected that in the case of *Fast Responses,* the averaging procedure used to determine brain activation would likely lead to higher values in the areas involved in fact retrieval, as compared to the *Slow Responses*^28^. This is because the average of signals across different trials highlights the activations that are consistent across all trials and more variable brain responses (i.e., in timing) are likely to be canceled out in the averaging. We expected a higher consistency for *Fast Responses,* as compared to the more variable *Slow Responses*, and that this would lead to higher brain activation detected for the *Fast Responses.*

## Results

### Behavioral results

Participants had a mean accuracy of 87% (range 70–98%) in the task. The results on only accurate responses were used to distinguish *Fast* or *Slow Responses* (see “Behavioral data pre-processing and trial categorization”). *Fast Responses* had an average response time of 930 ms (SD 110), whereas *Slow Responses* had an average response time of 1580 ms (SD 200). *Fast Responses* also showed significantly lower variability across participants as compared to *Slow Responses* [F(1,20) = 273.54, p < 0.001].

Details on the behavioral analysis of *Fast* and *Slow Responses* across participants and across runs are reported in the Supplementary Information. No participant reported discomfort during the task in the short debriefing performed at the end of the task.

### MEG results

#### Source analysis on ROI

**Figure 1:**
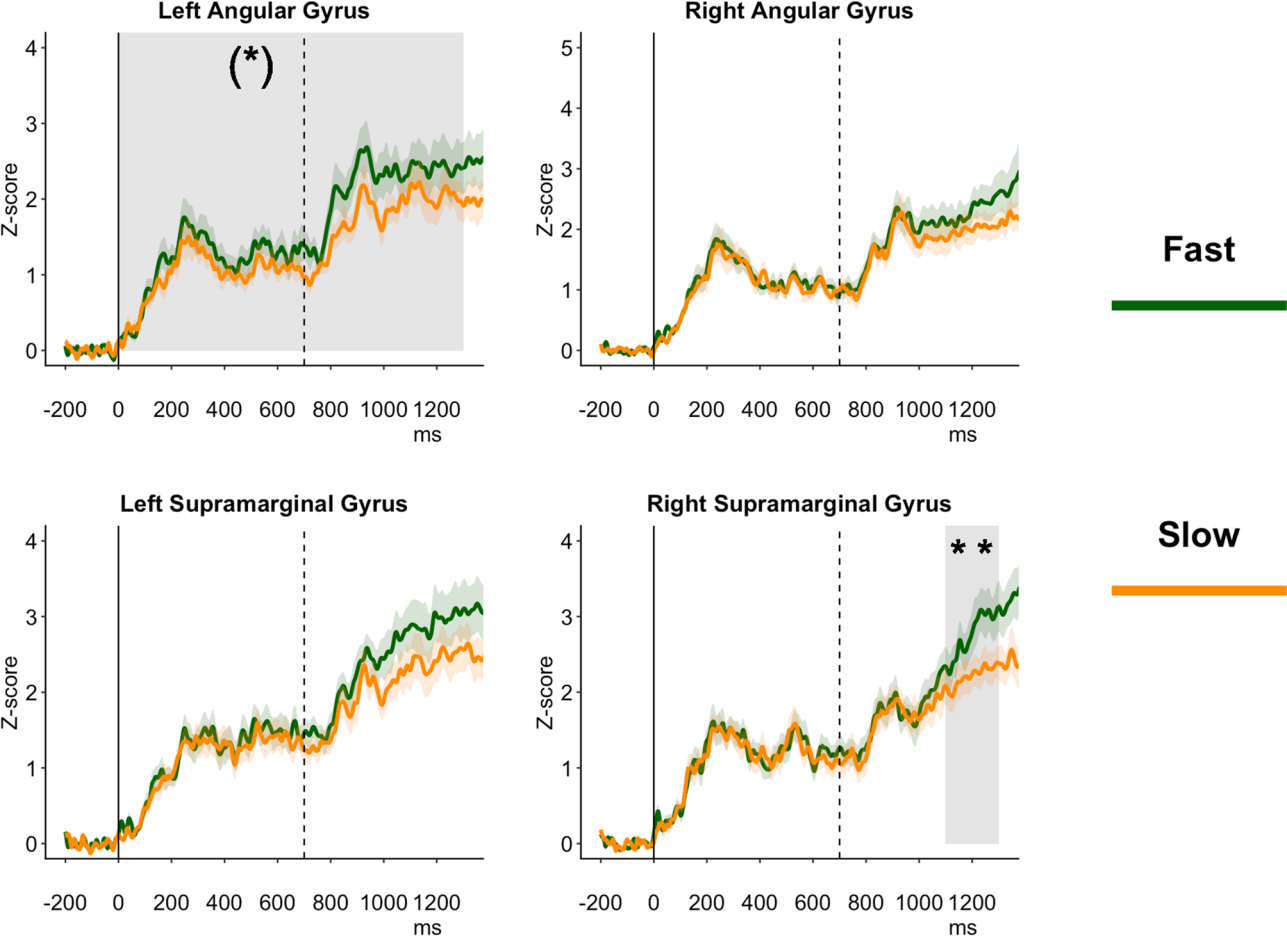
ROI analysis time-locked to the First Number. The figure shows the Source Activity z-scores for each Region of Interest (ROI). Each source waveform represents the average time series of z-scores of all participants within an ROI. Z-scores were calculated from source absolute values of activation. Shaded bands indicate standard errors around the mean. The gray areas and asterisks enclosed in round brackets ("(*)") highlight the time windows in which the ANOVA showed significant differences in the main effect. The asterisks without square brackets ("*") indicate the time windows in which there was a difference in the post-hoc analysis. The solid vertical lines indicate the appearance of the *First Number*, whereas the vertical dashed lines indicate the appearance of the *Second Number*. The figure was drawn by the first author of the paper—Giorgio Arcara.

Results on the source activity on a priori selected ROI are shown in Fig. 1.

##### Left angular gyrus

The ANOVA investigating the difference in z-scores after the *First Number* on Left Angular Gyrus showed a main effect of *Response* [F(1,20) = 9.74, p = 0.03, ges = 0.03], and a significant effect of *Time Interval* [F(12,240) = 23.55, p < 0.001, ges = 0.30]. The main effect of *Response* was associated with higher z-scores for *Fast* as compared to *Slow Responses*. The effect of Time Interval indicated a gradual increase of z-scores after the presentation of the *First Number*, with a peak at around 900–1000 ms after the *First Number*, which is about 300 ms after the presentation of the *Second Number*.

##### Right angular gyrus

The ANOVA investigating the differences on Right Angular Gyrus showed only an effect of interval [F(12, 240) = 20.71, p < 0.001, ges = 0.31]. Corrected post-hoc t-tests investigating this effect showed that the mean z-scores were different across different intervals, increasing almost monotonically and with the highest value in the last time window, 1200–1300 ms after the *First Number* (that is 500–600 ms after the *Second Number*).

##### Left supramarginal gyrus: first number

The ANOVA on the z-scores after the *First Number* on Left Supramarginal Gyrus showed a significant main effect of *Time Interval* [F(12, 240) = 33.73, p < 0.001, ges = 0.26]. Z-scores increased monotonically, with the highest value in the last time window (1200–1300 ms after the *First Number*, that is 500–600 ms after the *Second Number*).

##### Right supramarginal gyrus: first number

The ANOVA on the *First Number* on the Right Supramarginal Gyrus showed a significant effect of Interval [F(12, 240) = 26.17, p < 0.001, ges = 0.36], and a significant interaction between *Response* and *Time Interval* [F(12, 240) = 4.07, p = 0.03, ges = 0.02]. The post-hoc t-tests investigating the significant interactions showed that *Fast Responses* and *Slow Responses* were significantly different only in the last two time windows (1100–1200 ms and 1200–1300 ms), that is from 400 ms after the presentation of the *Second Number*.

### Whole-brain cluster-based permutation analysis of source activity

**Figure 2:**
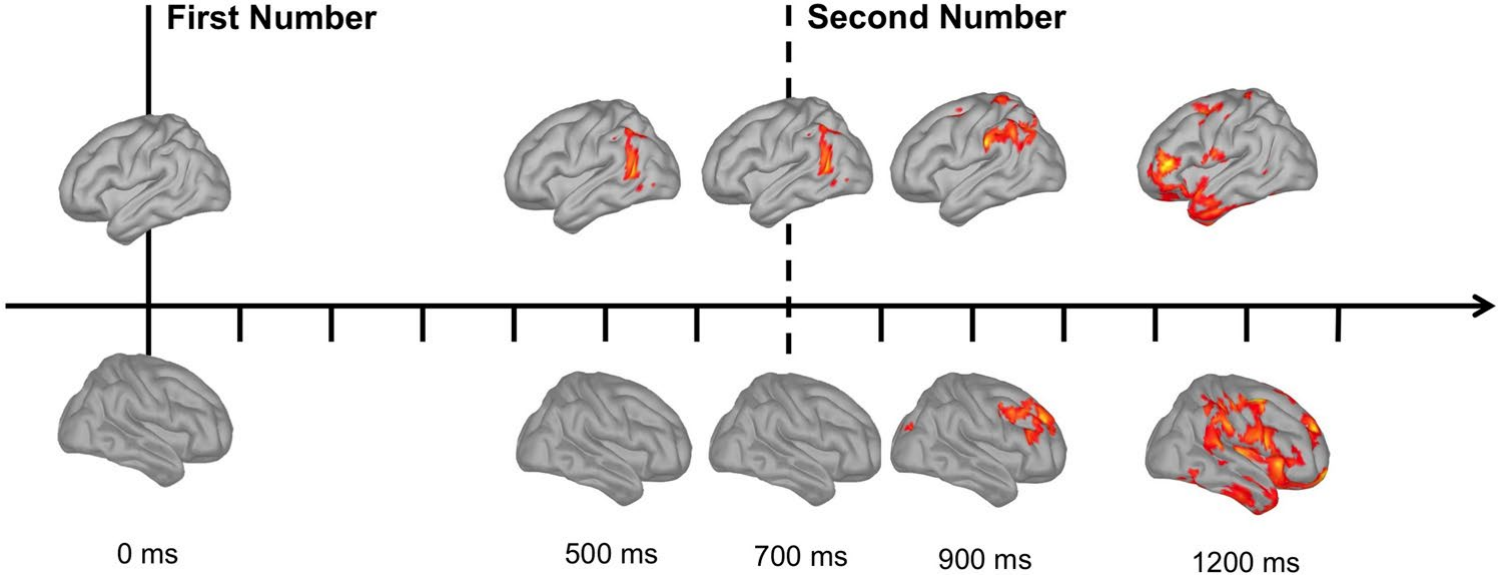
Results of cluster-based permutation analysis. The figure shows a schematic representation of the results of cluster-based permutation analysis, comparing *Fast Responses* and *Slow Responses* (*Fast* > *Slow*). Colors highlight the areas in which the significant effects were mostly localized. In all cases, *Fast Responses* were associated with higher values (z-scores) compared to *Slow Responses*. The solid vertical line indicates the appearance of the *First Number*, whereas the vertical dashed line indicates the appearance of the *Second Number*. Differences were initially found in the left hemisphere (mostly in the Angular Gyrus), starting at about 500 ms after the *First Number* and lasting also after the second stimulus. Within the right hemisphere, significant differences were found about 200 ms after the presentation of the *Second Number* in the right frontal cortex, followed by a more widespread effect starting at encompassing also the right parietal cortex. The figure was drawn by the first author of the paper—Giorgio Arcara.

The cluster-based permutation analysis showed significant differences between *Fast* and *Slow Responses*. In all cases, the analysis pointed to higher activations for *Fast* as compared to *Slow Responses*. In particular, two clusters were found, capturing the effects on the two hemispheres [*ps* = 0.009]. Results on the first cluster were started about 500 ms after the presentation of the *First Number* and were localized in the left hemisphere, especially in the Angular Gyrus. This higher activation was also visible in the rest of the analyzed time points until approximately 1000 ms. However, after the presentation of the *Second Number*, larger activations for *Fast Responses* were also found in the left frontal lobe, close to the Left Inferior Frontal Gyrus (see the upper part of Fig. 2).

The second cluster captured the effects localized in the right hemisphere, again with higher activation for *Fast Responses* as compared to *Slow responses*. This effect was evident from about 900 ms after the presentation of the *First Number* (that is about 200 ms after the presentation of the *Second Number*) and was localized initially in the right Dorsolateral Prefrontal Cortex (DLPFC). On later time points (at 1200 ms, approximately 500 ms after the *Second Number*), the effect was also localized in the Right Supramarginal Gyrus, in nearby areas of the right parietal cortex, together with the Right Insula and Right Frontopolar areas (see the lower part of Fig. 2).

### Time–frequency ROI analysis

**Figure 3:**
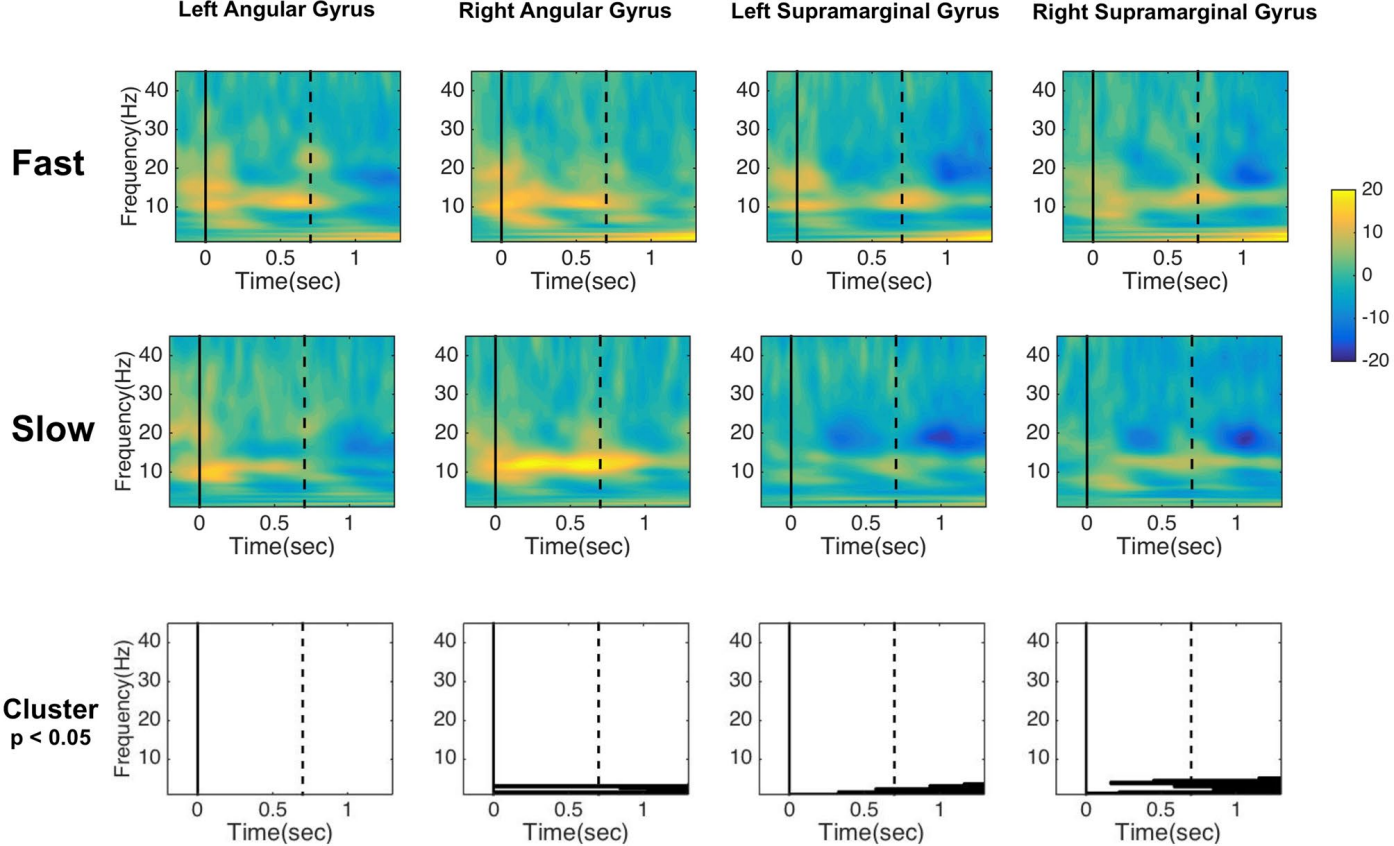
Results of time–frequency analysis on *Fast* and *Slow Responses *. The figure shows the results of the time–frequency analysis based on Morlet deconvolution, comparing *Fast Responses* and *Slow Responses*. The two upper rows show the average response of the ERS/ERD change relative to a baseline window of − 500 to 300 ms. The first row depicts the average results in each ROI for *Fast* and *Slow Responses*. The bottom row shows the significant difference calculated with cluster-based permutation, with black areas denoting the time points and frequencies belonging to the cluster with significant effects. In all significant effects, *Fast Responses* had higher magnitude values than *Slow Responses*. The significant differences were found for slow frequencies, mostly in the delta and partially in the theta range. The solid vertical line indicates the appearance of the *First Number*, whereas the vertical dashed line indicates the appearance of the *Second Number*. For a better inspection of the results, larger figures are reported in the Supplementary Information. The figure was drawn by the first author of the paper—Giorgio Arcara.

Results from the cluster-based permutation performed on time–frequency analysis showed a significant difference between *Fast* and *Slow* in three ROIs: Right Angular Gyrus [p = 0.01], Left Supramarginal Gyrus [p = 0.008], and Right Supramarginal Gyrus [p = 0.002]. Significant differences were mostly observed in the delta frequency band (2–4 Hz), and, in the Right Supramarginal Gyrus, in the theta range (4–5 Hz). The effects in Delta were observable in all the analyzed epochs in Right Angular Gyrus, Left Supramarginal Gyrus, and Right Supramarginal Gyrus. The effect in Theta in the Right Supramarginal Gyrus was already observable after the presentation of the *First Number*. Results are reported in Fig. 3.

### Correlation analysis between source activity and response time

The correlation analysis investigating the relationship between source activity (average in the time windows included in the ANOVA) and Response Time did not show any significant correlation after correction for multiple comparisons. Detailed results are reported in the Supplementary Information.

## Discussion

The present MEG study investigated the temporal involvement of the left and right hemispheres (with a focus on the left and right parietal lobes) in performing simple multiplications. Participants were asked to say aloud the result of single-digit multiplication problems, with the two operands (*First Number* and *Second Number*) presented sequentially. We designed this experiment to overcome some limitations of the past literature of neuroimaging and neurophysiology studies on mental multiplication, which mostly relies on verification tasks (i.e., after the operation is showed, the results are presented, and the participants are asked to respond whether the proposed solution is correct or not) in which participants may use some sort of familiarity judgment instead of computing an answer. The present production task more closely reproduces the behavior of performing a simple multiplication. It is more similar to the tasks used in studies with neuropsychological patients or using DCE during neurosurgery, filling the gap between the different fields of literature. We compared *Fast* and *Slow Responses*, determined on an individual basis^30,32^, using the rationale that the higher activation in the *Fast Responses* will unveil the more efficient (i.e., fast) route for accomplishing the task. MEG data were analyzed using source activity and time–frequency analysis, which provide strictly related, but complementary, information.

In the ROI analysis on source activity, focusing on the left and right parietal lobes, we found an initially higher activation of the Left Angular Gyrus for *Fast* as compared to *Slow Responses*, starting after the presentation of the *First Number* and lasting after the *Second Number*, up to few hundred milliseconds before responding. We also found a significantly higher activation for *Fast Responses* after the presentation of the *Second Number* (400–500 ms and 500–600 ms after its appearance) in the Right Supramarginal Gyrus (see Fig. 1). The results were in line with the bottom-up analysis using cluster-based permutation. Although results of cluster-based permutation analysis cannot be used to identify when and where some effects are present (due to the way the null hypothesis is defined in this analysis, Maris & Oostenveld 2007), they could be seen as suggestive of potential effects and the potential starting points for further other analyses in future studies. A qualitative inspection of the results showed higher activation in *Fast Responses* as compared to *Slow Responses*, mostly expressed in left parietal areas, then in right frontal areas, and finally also in left frontal areas and, in a more widespread fashion, in centro-parietal and frontal areas of the right hemisphere.

These results, obtained within a simple production task, are surprisingly similar to those employed recently with a more traditional verification task, also in MEG. Indeed, Salillas et al.^3^ found that in the case of a comparison of correct vs incorrect solutions in single-digit multiplication, a complex network of areas, encompassing both parietal and frontal areas of the left and right hemisphere, were involved, peaking about 600 ms after the solution was presented. We observed a similar pattern, especially in a late time window, about 500 ms after the *Second Number*. These similarities, as seen from one side, suggest that production and verification tasks engage similar areas in simple multiplication, and that results from a verification task are not excessively distorted by the design. However, production and verification tasks also showed some differences. In the production task, we found an earlier involvement of the left parietal cortex, after the first operand was presented, which lasted during the presentation of the second operand. This resembles the results found with a verification task using ERP at electrode levels by Kiefer and Dehaene^8^, who also investigated responses after the second operand and who found an initial left-lateralized response for a priori defined easy multiplications^8^. These results underline a similarity between verification and production tasks, also highlighting the importance of analyzing brain responses not only after the solution is presented^3,23^ but additionally after the second operand is presented. This initial involvement of the left hemisphere is in line with traditional accounts that state a role of left parietal areas for retrieving results in simple multiplications (i.e., fact retrieval).

Interestingly, significant differences between *Fast* and *Slow Responses* were already observed after the *First Number*, but before the *Second Number*. Since, at that stage, it was not possible to retrieve the result of the multiplication, this activation could reflect other generic processes, such as a higher allocation of attention. Alternatively, we may speculate that this activation in this stage could be related to a preliminary selection of some possible results. Concerning this last possibility, in the context of this experiment, each *First Number* could be followed only by 7 possible other numbers (same number multiplication, and multiplication with 1 and 0 were excluded), narrowing the actual potential results that should be retrieved. For example, if the *First Number* is 8, the potential results are 16, 24, 32, 40, 48, 56, and 72. Thus, it is possible that after seeing the *First Number,* a participant starts to select a candidate result (or several candidate results) and the higher activation of the Left Angular Gyrus for *Fast Responses* captures this effect. This interpretation would be again in line with the DCE findings during awake surgery, which shows table selection errors (e.g., 7 × 3 = 28) when the left parietal lobe takes over the task after inhibition of the right parietal lobe^10,11^.

Within the right hemisphere, about 500 ms after the *Second Number*, a higher activation was found in the right frontal areas, especially in the DLPFC and in the right insula. The activation of the right DLPFC could be related to monitoring processes involved during the retrieval^33,34^ or to working memory demands^1^. The activation of the right insula could suggest that *Fast Responses* are associated with consistent involvement of attention in the same time windows^35^. There was not a higher involvement of the frontal lobe in the *Slow* as compared to *Fast Responses*, in contrast with fMRI findings^15^. Given the methodology employed, this result is not surprising. The temporal precision of MEG allowed us to track the relatively higher (and faster) activations of left and right areas during fact retrieval. However, if the activation of an area is inconsistent across trials, or participants, the high temporal resolution of MEG is detrimental to the possibility of capturing the effect. In fact, the averaging procedure cancels out the activations that are not consistent over time. This is confirmed by analysis of RTs (see also Supplementary Information) and is the case of an expected higher frontal activation in *Slower Responses*. Hence, these results do not imply that there is no involvement of frontal areas in *Slow Responses*, but that in *Fast Responses* there is a consistent activation of right frontal areas before providing the answer.

To shed further light on multiplication processing we used time–frequency analysis. In the existing literature on arithmetic processing, time–frequency analysis was used to unveil the neural correlates of different strategies used to accomplish the task^17^. We decided not to focus on specific a priori frequencies, and we opted for a bottom-up approach, investigating the frequencies from 1 to 45 Hz. Statistical analysis showed reliable differences between *Fast* and *Slow Responses* in both hemispheres, with effects restricted to the lowest frequencies, in the range of delta (2–4 Hz) and theta (5–7 Hz). As compared to source activity, time–frequency analysis showed significant differences between *Fast* and *Slow Responses* in slightly different areas, as no difference was found in the Left Angular Gyrus but in the Left Supramarginal Gyrus, and effects were also found for the Right Angular Gyrus. Although one can be tempted to conclude that time–frequency analysis captured qualitatively different processes as compared to source activity, as the two areas were very close, a more cautious interpretation is that this difference is related to the limits in the spatial resolution of the MEG. The results in the delta range resemble the effects obtained in the source activity analysis and are partly related to the slow-wave enhancement that generates the source activity components^21^ (we explored this aspect in an additional exploratory analysis, which showed that some of the low-frequency effects were no longer significant after removing the evoked activity, i.e., phase-locked, before performing the statistical analyses; see Supplementary Information) Importantly, these results confirm the role of both the Left and Right parietal lobes in fact retrieval. Given the low-frequency range and the temporal smearing due to the Morlet deconvolution, we cannot speculate about the timing of effects on the delta range. Interestingly, within theta frequencies (about 5–7 Hz), we found slightly higher temporal resolution. Results showed higher magnitude changes in theta in the *Fast* rather than *Slow Responses*, already after the presentation of the *First Number*. We may hypothesize that this is a general orienting activity and cognitive control, which has been found with frontal and parietal distribution, for optimal target processing and decision making^36–38^. The fact that the increased theta magnitude began soon after the presentation of the *First Number,* and continued during the presentation of the *Second Number,* can be associated with an orienting activity or with the role of theta oscillations in the successful recollection of memories and source retrieval^39,40^. Of note, higher theta modulations have been previously associated with fact retrieval^14^. Thus, these results would be in line with the traditional account of the role of the left hemisphere in mental multiplications^4^, supported and extended by the more recent development of DCE studies^10^.

A qualitative inspection of time–frequency results also showed other interesting aspects. For example, *Slow Responses* were associated, in the upper alpha band, with lower magnitude, as compared to *Fast Responses* in the last 200 ms of all ROIs (see Figures in the Supplementary Information for larger versions of time–frequency results). In studies focused on additions^19^, the authors found that lower magnitude (i.e. desynchronization) in upper alpha (10–13 Hz), as compared to baseline, was associated with the use of a specific strategy to solve the problem, that is to rely on procedural rules^17,19^. Importantly, because these differences in the alpha band were not statistically significant, we can only speculate that this pattern of time–frequency is also related to strategy differences between *Fast* and *Slow responses*. Future studies focusing on these frequency bands could explore this possibility. Importantly, we did not explicitly assess for the strategy used by the participants (see for example^41^), neither did we suggest them to use specific strategies, but given the observed reaction times (see also Supplementary Information), it is reasonable to conclude that in the case of *Fast Responses* a direct retrieval (i.e., a “fact retrieval”) from memory occurred.

Altogether, these results, integrated with both existing neuropsychological and DCE^10,11^ and neuroimaging and neurophysiological literature, allow us to sketch a tentative explanation of the involvement of the left and right hemispheres in single digit multiplication. We speculate that this task could be related to the activation of the left parietal cortex (in particular, the Left Angular Gyrus), reflecting the initial retrieval of potential candidates. This activation is followed by the activation of the Right Supramarginal Gyrus, which could reflect a process of checking the magnitude of the possible candidates retrieved by the left hemisphere. The left hemisphere plays a crucial role in the retrieval process but it is, at the same time, prone to errors, insofar as it may choose one table result that is next to the wanted one^10^. By contrast, one of the putative functions of the right parietal lobe would thus be to help identify (and perhaps amend) these errors. Thus, the present data seem to be compatible with a view that the right hemisphere always enters, automatically, into this play, even when the process is fast and smooth. Interestingly, the proposal of a "quality check" role of the right supramarginal gyrus is also in agreement with findings from a variety of numerically related tasks including quick decisions about the number of syllables in a word^42^ and significant disruption of time judgments when rTMS was applied to this area^43^. Finally, the activation of other more frontal areas could reflect the motor execution (Left Inferior Frontal Gyrus) and some additional monitoring processes to check if the selected result is correct (Right Dorsolateral Frontal Cortex). However, although consistent with the present findings, this interpretation needs to be confirmed by future research that could try to characterize whether the interference of the right parietal regions is detrimental only to mathematical processes or it generalizes to other kinds of mental representations.

A potential criticism of this study is that the effects could reflect motor preparation. However, there are several arguments to refute this interpretation. First, if the observed activations were merely a consequence of articulatory preparation we would have expected a high correlation between response times and the brain activation (which was not the case, see Supplementary Information). Second, if the effect was merely an effect related to motor preparation we would have expected similar (but) shifted, time courses of source activity in *Slow* vs *Fast* responses, but this is not the case, as the time courses are mostly overlapping. Third, the different activations across conditions are not in the areas that are expected in tasks that include simple retrieval of a word (i.e., simple naming task). Indeed, a naming task in MEG shows typical activations in the classical areas of language production (mostly left-lateralized), but not in right frontal areas^44^. Even if right parietal effects may be found, they are expected to be in earlier time windows^44,45^. Finally, a motor preparation effect is expected to be associated with a decrease in beta frequencies, which should be higher and more consistent in the case of *Fast* as compared to *Slow Responses*. Time–frequency plots (as clearly in Fig. 3) suggest that this is not the case. There was no significant difference in betas across conditions, but rather there is a qualitative difference in the opposite direction, with a larger beta decrease in the case of *Slow Responses*.

### Limitations

The present study has some limitations that need to be underlined. First, given the correlational nature of MEG recordings and the type of contrast used (*Fast* vs *Slow Responses*), we cannot disentangle which area is “necessary” for a given operation or the areas that are always involved. Rather, the contrast between *Fast* and *Slow Responses* shows the activations that are more likely to be present before a *Fast Response,* that is, an efficient response. The actual importance of these activations should be corroborated by neuromodulation studies (e.g., TMS^7^). Secondly, the sample included in the current study was mostly composed of people with a degree in Psychology or Linguistics (see Supplementary Information for details, Supplementary Table S1). Third, our dichotomous distinction between *Fast* and *Slow Responses* is also just one of the possible methods to classify the responses, and finding the optimal method to study response efficiency (in terms of speed) is an issue that could be investigated in future studies. A third issue concerns the potential bias driven by signal variability, which, also according to our hypotheses, could be different across conditions. An exploration of this aspect can be found in the Supplementary Information. Shortly, we found that in the analysis on source activity there was no systematic difference in variability between conditions, while some differences were found in TF analysis, related to the mathematical operations of the analysis itself (see Supplementary Information).

Another final aspect to take into account is the potential overlap between response efficiency (as we classified in the present study) and problem size. There is a wide literature on the effects of problem size in mental multiplication^12,19,46,47^. “Large problems”, that is problems including multiplication with larger numbers (e.g., 7 × 9), require longer reaction times than “small problems” (e.g., 2 × 3). In the present study, it is not possible to tease apart the two effects because, often, fast responses were related to small problems (see Supplementary Information, Supplementary Table S2); however, this was not always the case. Future studies with a larger number of multiplications included in the experimental settings could have enough stimuli to better address this aspect and clarify (and distinguish) if specific and different routes depend on efficiency and on problem size, and/or their interaction.

## Conclusions

In summary, the findings of the present study fill the gap between neuropsychological and DCE studies on the one hand—which used mostly production tasks—and neuroimaging and neurophysiological studies on the other hand—which used mostly verification tasks. We interpret the results as consistent with recent literature and compatible with the idea of joint participation of the two hemispheres in single-digit multiplications^10^. Using an approach based on a comparison of fast and slow responses, results showed a higher activity for fast responses in the left angular gyrus starting after the first operand and in the right supramarginal gyrus after the second operand. The sequence of brain hemisphere involvement would be routinely adopted by literate people for problems that do not require extra effort, presumably the easiest and most practiced ones. The specific areas sub-serving these processes seem to be, consistent with most (permanent and temporary) lesion and neuroimaging studies, the angular and the supramarginal gyrus in both the left and the right hemispheres. The results we presented open several new questions on the potential role of parietal lobes and other frontal areas during fact retrieval that could be addressed by further research on the field.

## Materials and methods

### Participants

A sample of 21 healthy participants voluntarily took part in the study (11 female and 10 male). We excluded participants with any neurological or psychiatric disease that could affect cognitive performance or who had a history of developmental dyslexia or dyscalculia. The mean age of participants was 26 years (SD 3, range 20–32, Q1 = 24, median = 25, Q3 = 28) and their mean education was 17 years (SD 2, range 13–22, Q1 = 15, median = 18, Q3 = 18). All participants were right-handed. Details on participants are reported in the Supplementary Information.

### Ethics statement

The study was approved by the local ethics committee (Comitato Etico per la Sperimentazione Clinica della provincia di Venezia e IRCCS San Camillo) and conducted following the guidelines of the Declaration of Helsinki.

### Recording procedure

Before entering the magnetically shielded room, participants underwent initial preparation, which consisted of the placement of three head coils, to monitor head position during MEG recording, and eight external electrodes. These electrodes were used to record VEOG, HEOG, ECG (bipolar montage), and the muscular activity of the mouth, with two electrodes placed on the orbicularis oris (upper left and lower right corners of the mouth).

Continuous MEG signal was acquired using a whole head 275-channel system (CTF-MEG). Data were sampled at 1200 Hz, with a hardware anti-aliasing low pass filter at 300 Hz. Before the experimental session, participants underwent a 5-min resting state session (not analyzed in the present work) and then undertook the experimental task that consisted of three runs of 5 min each. The total duration of the experiment was approximately 30 min. After the MEG recording session, three Vitamin-E capsules were placed exactly on the coil positions and the participants completed an MRI scan (Philips Achieva, 1.5 T). This procedure allowed the co-registration of MEG data and MRI data. The MRI scan was always performed on the same day of the MEG recording. In all recordings, head movements never exceeded the threshold of 5 mm on any axis.

### Task procedure

Upon signing the informed consent form, participants were instructed that they would be required to perform simple calculations during a MEG recording. Before the beginning of the experiment, and after the instructions were given, the participants had the possibility to rehearse the multiplication tables, to limit the potential effect of anxiety on the task performance. In a pilot study, several participants asked if it was possible to rehearse the multiplication tables before performing the task and reported to be distressed not to have this opportunity. This possibility was only given before the experimental trials.

The task recording session was divided into three runs of 5 min each, with small breaks between each run. The experiment was programmed with the free software Psychopy^48^ (version 1.82), running on a PC. The task was a mental multiplication with two one-digit numbers. Participants were required to say aloud the result of the multiplication of the two numbers.

**Figure 4:**
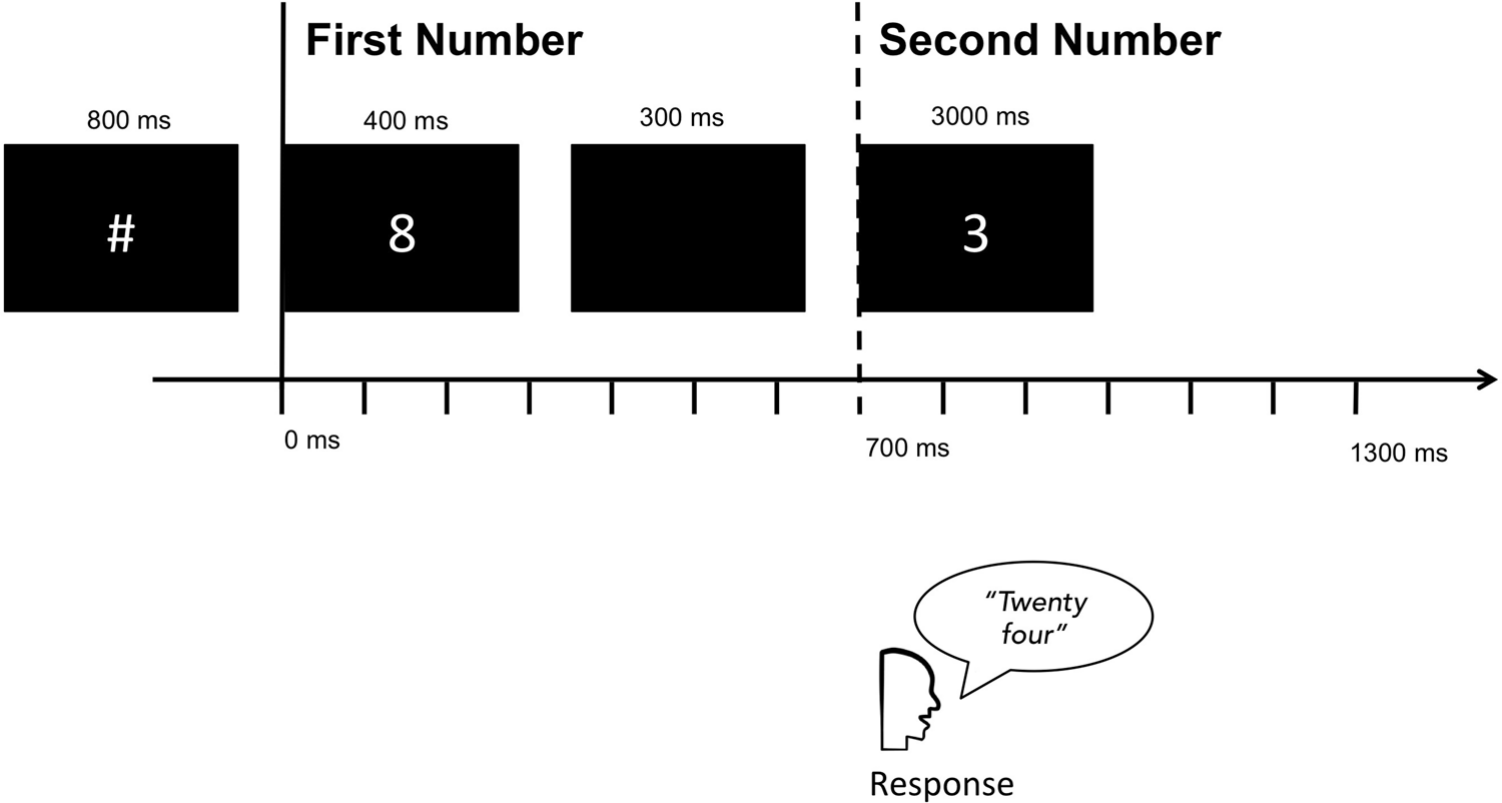
Schematic representation of the Task. The figure illustrates the task procedure. The participants were asked to answer after the *Second Number*, providing the results of the multiplication between the numbers. Analyses were conducted on source activity time-locked to the *First Number* (8 in the example, solid vertical line) and to the *Second Number* (3 in the example, dashed vertical line). The space between two ticks in the x-axis indicates a 100 ms interval. The figure was drawn by the first author of the paper—Giorgio Arcara.

Each trial was organized as follows: first, a fixation symbol (#) was presented for 800 ms, followed by the first number (henceforth, *First Number*) presented for 400 ms, a blank screen for 300 ms, and then a second number (henceforth, *Second Number*) lasting for 3000 ms. Participants were instructed to respond as quickly and as accurately as possible, saying aloud the response before the *Second Number* disappeared (see Fig. 4), which was up to 3000 ms after the *Second Number*. They were explicitly instructed to avoid hesitations and mouth movements before the response. Before the beginning of the experimental task, participants underwent a familiarization session with five stimuli. All stimuli subtended about 1.5° of visual angle on the horizontal plane. Stimuli were presented using white Courier font on a black background screen. We recorded the latency of the oral response after the *Second Number* and divided the correct responses into *Fast* or *Slow* on an individual basis.

### Stimuli

All combinations between one-digit numbers were used in each session, excluding same number multiplication (e.g., 5 × 5, 2 × 2) and multiplication including 1 or 0 as an operand (as in Ref.^23^). This led to 54 different operations that were repeated three times in the three sessions, which constituted the whole experiment. Within each session, the presentation of each possible combination was randomized.

### Behavioral data pre-processing and trial categorization

Behavioral data were analyzed to categorize responses given by participants as *Fast* or *Slow,* on an individual basis. This methodology has been previously applied in several domains. Van den Berg and colleagues, for example, analyzed the EEG activity associated with fast and slow RTs within the same individual in a task of visual-search^29^. In another choice-response task, trials of correct responses were ordered by RTs in quartiles and the EEG activity was analyzed separately^32^. Again, a similar methodology was also applied by Novikov et al.^30^ who performed within-subject EEG analysis with a distinction between fast and slow behavioral responses (based on the individual medians), to investigate the two different mechanisms that underlie response-speed during a sustained attention task. Similar to the above-mentioned strategies, in our study, efficient (i.e., correct and fast) and inefficient (i.e., correct and slow) responses were compared on an individual basis^31^. In our case, for *Fast Responses,* the brain is likely to follow a practiced, quick, and efficient route to retrieve the correct response, whereas in *Slow Responses* it is likely that less efficient (and more variable) strategies are utilized^28^. Notably, participants provided correct answers also in the trials with a *Slow Response* (ensuring that the stimuli were processed), but the slower response time indicates that the processing was less optimal.

Vocal responses were recorded as .wav files with Psychopy used to define the onset of the response from the *Second Number*, using custom code and the CheckVocal free software^49^. After defining the vocal response times, correct responses from each subject were divided into three equal groups. *Fast Responses* were operationally defined as the 18 fastest correct responses within a run, calculated separately for each participant. *Slow Responses* were operationally defined as the 18 slowest correct responses within a run, calculated separately for each participant. The remaining 18 intermediate responses were not used for further analyses. Using this procedure, the *Fast Responses* corresponded approximately to scores below the 33rd percentile, and the *Slow Responses* correspond approximately to the scores above the 66th percentile. Previous studies have already applied analogous strategies of stratifications in several domains to create different quantiles of the response-time distribution^32,36,50^. The separation we adopted was chosen to ensure the highest possible number of stimuli and the highest possible separation between *Fast* and *Slow Responses*. As the experiment included three runs, this led to a total of 54 trials (18 × 3), before trial rejection, for each subject, for each condition (*Fast* vs *Slow*). This strategy yielded a reasonable number of trials to have a satisfactory signal-to-noise ratio, as qualitatively determined in pilot studies. Importantly we do not claim that data are distributed bimodally as Fast and Slow (this was confirmed also by visual inspection of data distribution), but the *Fast* vs *Slow* categorization allowed us to rely on well-known statistical analyses for MEG data at the source level (i.e., cluster-based permutation). To investigate whether our hypothesis (of increased variability for *Slow Responses*) was supported by the data, we conducted an ANOVA, using the standard deviation of RT of responses for each participant within *Fast* as opposed to *Slow Responses* (see Supplementary Information, Supplementary Fig. S.1.2). Details on changes of reaction times over runs, as well as other additional details, are reported in the Supplementary Information.

### MEG preprocessing

MEG data pre-processing followed a standard pipeline that included filtering of continuous data (high pass: 0.1 Hz, notch filter 50 Hz and harmonics), artifacts removal with Signal-Space Projection algorithm (SSP), data segmentation (epochs starting from − 2 s from the *First Number* to 3.7 s, which was 3 s after the *Second Number*), trial rejection of epochs to check for residual artifacts. For extensive details on MEG preprocessing see Supplementary Information.

### Source estimation

For the source analysis, individual T1 MRI scans were used. The MEG forward model was calculated with the overlapping spheres method and the source reconstruction was performed with the wMNE (weighted Minimum Norm) algorithm, calculated on the cortex. The noise covariance was calculated from 3 min of empty room recording. Details on the source estimation procedure can be found in the Supplementary Information.

### Source activity analysis

To calculate source activity, from each initial epoch of MEG recordings, a smaller set of epochs was then extracted. This set of epochs was time-locked to the *First Number* (with − 200 ms before the *First Number* used for baseline correction) and lasted from − 200 to 1300 ms after the stimulus, Thus the epoch included the presentation of the *First Number* from 0 to 400, a blank from 400 to 700, and the presentation of the *Second Number* from 700 to 1300. We limited the analysis to 600 ms after the *Second Number* (that is 1300 ms after the *First Number*) because it was before the fastest response time across all participants, which was at 608.5 ms. Sensor data from trials were averaged to obtain the source activity and filtered with a low-pass filter at 40 Hz. Averages were made separately for Response Type (*Fast* vs *Slow*). Source activity analyses were divided into two main groups: ROI (Regions of Interest) analyses and Whole-brain analyses.

In the ROI analysis, MEG data were analyzed on a priori selected ROIs, chosen according to the hypotheses and from the existing literature. For this analysis we focused on the following ROIs: Left Angular Gyrus, Right Angular Gyrus, Left Supramarginal Gyrus, and Right Supramarginal Gyrus from the Destrieux atlas^51^. ROIs were defined according to the FreeSurfer automatic parcellation of cortex surface^52^. For both ROI and whole brain-analysis, sensor data were rectified and projected to the cortex using the common kernel and were projected on a common template (MNI, Colin27), made by 15,000 vertices, using the FreeSurfer registration method implemented in Brainstorm^53^. Data were then transformed in z-scores, using the same baseline employed for the baseline correction for the ERF (i.e., − 200 to 0 ms before the First Number, see Supplementary Information). Resulting data and then spatially smoothed with a Gaussian kernel with Full-Width Half Maximum (FWHM) of 3 mm. For the ROI analysis, we extracted z-scored time series from each ROI using the mean values of all vertices contained in that ROI.

### Time–frequency analysis

To provide a more complete picture of the MEG activations, we also used time–frequency analysis. For the time–frequency (TF) analysis, starting from the initial trials (− 2 to 3.7 s), we extracted at single-trial level MEG time series, separately for each ROI. These time series were calculated as the first components of a fast PCA on the time series of all vertices belonging to the ROI (based on the built-in Brainstorm function). We then performed a Morlet Wavelet time–frequency decomposition of this extracted signal on a broad range of frequencies, ranging from 1 to 45 Hz (with a step of 1 Hz). Wavelets were built starting from the mother wavelet with 1 Hz and 3 s of Full-Width Half Maximum (FWHM), and generating all remaining wavelets from this initial one, keeping constant the number of cycles across frequencies.

With the time–frequency decomposition, we extracted the Magnitude values separately for each frequency at the single-trial level. These values were averaged across runs, separately for each condition, such that two averages were obtained for each participant—one for *Fast* and one for *Slow Responses* (with values for each ROI). We finally calculated the Event-Related Synchronization/Desynchronization (ERS/ERD), using as baseline the − 500 to 300 ms time window before the *First Number*, by applying the formula [(Epoch_Signal − Baseline_mean)/(Baseline_mean)] × 100.

### Statistical analyses

Behavioral and MEG data analyses were performed with erpR package^54^. For the analysis of source activity at the ROI level, preliminary visual inspection showed that data were normally distributed. Therefore, parametric analyses, including ANOVA and t-tests, were used. All analyses, that is, ROI analysis of source activity, Whole Brain source activity, and Time–frequency, were performed on the same epoch interval, that is from the presentation of the *First Number* to 600 ms after the presentation of the *Second Number* (so from 0 to 1300 ms after the *First Number*).

ROI analyses were performed by means of repeated measures ANOVAs, separately for each ROI. The analysis included the following variables: *Time Interval* (thirteen levels: 0–100 ms, 100–200 ms, 200–300 ms, 300–400 ms, 400–500 ms, 500–600 ms, 600–700 ms, 700–800 ms, 800–900 ms, 900–1000 ms, 1000–1100 ms, 1100–1200 ms, 1200–1300 ms) and *Response* (two levels: *Fast, Slow*). Data for each time interval were calculated as arithmetic means of all values within the interval. Due to the relatively small number of participants, there was no possibility to include more factors in the ANOVAs. Given the potential differences in responses and signal range across ROIs, we used four separate ANOVAs, one for each ROI. For the sake of completeness, we also completed an additional ANOVA analysis (reported in the Supplementary Information) using type 1 sum of squares. Although type 1 ANOVA is typically not recommended, it allowed us to also include the ROI factor in a single analysis. Interestingly, this additional ANOVA including the ROI factor, confirmed the results, showing an additional interaction of ROI, Response, and Interval, with an analogous pattern to the one reported in the manuscript. To compensate for inflated type 1 error, we corrected all p-values obtained across all ANOVAs for all ROIs (considered together) with False Discovery Rate correction for multiple comparisons (FDR)^55^. Post-hoc comparisons for significant ANOVA effects were calculated as paired t-tests, with FDR correction applied separately for each group of post-hocs. The effect size for each effect in the ANOVA was calculated as global eta squared (ges)^56^.

To analyze source activity at the whole-brain level, a cluster-based permutation approach was used, using data of all 15,000 vertices of source-reconstructed activations. We performed a single cluster-based permutation analysis, encompassing the overall epoch considered in all analyses, starting from the presentation of the *First number* (0 ms) to 1300 ms afterward (that is 600 ms after the *Second Number*). To reduce the computational burden of this analysis, and following standard indications^57^, we down-sampled the data to 150 Hz before performing the cluster-based permutation. In this analysis, the number of permutations was set to 1000, the minimum number of neighbors was set to 2, and the alpha level was set to 0.05.

To analyze time–frequency data, we performed a single cluster-based permutation on the 0–1300 ms time window (without downsampling), on all Frequencies and ROIs. In this analysis, the number of permutations was set to 1000, the minimum number of neighbors was set to 0 (because the topographic information was lost in the ROI time-series extraction procedure), and the alpha level was set to 0.05.

Finally, to analyze the relationship between source activity and Response times, we calculated a series of Spearman correlations between the average reaction time of the participants for *Fast *and for *Slow Responses *(separately), with the average source activity value for each ROI, separately for each level of *Time Interval*. To account for the large number of comparisons of correlations, p-values were adjusted with FDR correction.

## Supplementary Information

Supplementary Information.

## Data Availability

Data will be made available, upon permission from the host institution, as the data controller.

## Code availability

Code will be made available, upon request.
