# Supplementary material for: Magnetoencephalography reveals differences in brain activations for fast and slow responses to simple multiplications
Source: Sci Rep. 2021 Oct 13;11:20296. doi: 10.1038/s41598-021-97927-8 (PMC8514455; doi:10.1038/s41598-021-97927-8)
Supplement: Supplementary file 1 — Supplementary Information. [file 41598_2021_97927_MOESM1_ESM.docx]

**SUPPLEMENTARY MATERIALS**

The present file contains the supplementary materials of the article

**"Magnetoencephalography reveals differences in brain activations for fast and slow responses to simple multiplications"**

Arcara G.^1*^, Pezzetta R.^1*^, Benavides-Varela S.^2,3^, Rizzi G.^3^, Formica S.^4^, Turco C.^1^, Piccione F.^5^ and Semenza C.^3^

^1^ IRCCS San Camillo Hospital, Venice, Italy

^2^ Department of Developmental Psychology and Socialization, University of Padova, Padova, Italy

^3^ Department of Neuroscience (Padova Neuroscience Centre), University of Padova, Padova, Italy

^4^Department of Experimental Psychology, Ghent University, Ghent, Belgium

^5^Riabilitazione, Azienda Ospedale - Università di Padova, Regione Veneto, Italy

Additional materials, in particular files for interactive inspection of the MEG results can be found in the Open Science Framework project, at this permalink

<https://osf.io/b32xy/>

Details on Participants 2

MEG Preprocessing 3

Source estimation 3

Multiplication problems and classification into Fast and Slow Responses 4

Details on Response Times across runs 5

Time Frequency Plots 9

Time Frequency plot - Left Angular Gyrus 10

Time Frequency plot - Right Angular Gyrus 11

Time Frequency plot - Left Supramarginal Gyrus 12

Time Frequency plot - Right Supramarginal Gyrus 13

Additional Time Frequency analyses 15

Magnitude data distributions 15

Statistical results of Time Frequency analysis removing Evoked activity 16

Statistical results of Time Frequency analysis using Power 18

Time course of signal variability 20

Correlation of inversion kernel weights 25

Details on ANOVAs presented in the main manuscript 27

ANOVA - Left Angular Gyrus 28

Post-hoc Left Angular Gyrus – Response Type 29

Post-hoc Left Angular Gyrus – Time Interval 29

ANOVA - Right Angular Gyrus 33

ANOVA - Left Supramarginal Gyrus 37

ANOVA - Right Supramarginal Gyrus 42

Corrected P-values for ANOVAs across all ROI 56

Details on additional ANOVA 58

POST-HOCS for Additional ANOVAs 59

References 60

# Details on Participants

The table below reports some details on the participants.

| ID | Sex | Age | Years of Education | Title/Occupation |
| --- | --- | --- | --- | --- |
| MH001 | F | 25 | 18 | Master Degree in Psychology |
| MH002 | F | 25 | 18 | Master Student in Psychology |
| MH003 | M | 25 | 18 | Master Degree in Psychology |
| MH004 | F | 25 | 18 | Master Degree in Psychology |
| MH005 | F | 24 | 15 | Master Student in Theater |
| MH006 | F | 32 | 21 | PhD in Linguistics |
| MH007 | F | 30 | 18 | PhD candidate in Linguistics |
| MH008 | F | 22 | 15 | Lab Technician |
| MH009 | M | 28 | 15 | Lab Technician |
| MH010 | M | 27 | 18 | Engineer |
| MH011 | M | 32 | 22 | Medical Doctor with Specialzation in Internal Medicine |
| MH012 | M | 24 | 15 | Master Student in Medicine |
| MH014 | M | 24 | 15 | Master Student in Linguistics |
| MH015 | M | 26 | 15 | Master Student in Linguistics |
| MH016 | M | 28 | 18 | Master Degree in Psychology |
| MH017 | F | 24 | 18 | Master Degree in Psychology |
| MH018 | F | 24 | 18 | Master Degree in Psychology |
| MH019 | F | 20 | 18 | Master Degree in Psychology |
| MH020 | M | 25 | 15 | Master Student in Psychology |
| MH021 | F | 24 | 18 | Master Degree in Psychology |
| MH022 | M | 28 | 13 | High school graduate/Unemployed |

**Table S.1**. **Participants’ details.** The first column reports the participant’s ID (for comparisons with other tables). The second column reports the sex (Female or Male), the third column the age in years. The fourth column reports the number of completed years of education. The fifth column reports the type of Degree/school completed or the current job.

# MEG Preprocessing

MEG data pre-processing was performed with Brainstorm53 MATLAB toolbox (March 2015 version), which is documented and freely available for download online under the GNU general public license (<http://neuroimage.usc.edu/brainstorm>). Source activity in the Region of Interest (ROI) and behavioral data analyses were performed with the erpR package54. Continuous data were initially filtered with a notch (50 Hz and harmonics at 100, 150, 200 and 250 Hz) and high pass filter at 0.1 Hz. Then, Signal-Space Projection algorithm (SSP) was used to identify and remove cardiac and eye movement artifacts from the recordings. As opposed to independent component analysis (ICA), SSP is preferred for MEG signals, because MEG recordings are typically characterized by a high number of sensors and comparatively small number of timepoints, making ICA less reliable. SSP components with artefactual topography were removed by a trained experimenter. Triggers associated with stimulus presentation were thus divided according to the speed of the response, individually (see Behavioral data pre-processing and trial categorization section). To improve accuracy of trigger timing, digital triggers were adjusted off-line according to the actual stimulus presentation assessed with a photodiode using a Brainstorm built-in function.

After filtering and removal of artefacts with SSP algoritm, d ata were segmented into epochs starting from -2 seconds from the First Number to 3.7 seconds after the First Number. The length of the epochs was chosen to allow a complete visual inspection of the entire trial, from the fixation to the final oral response during the trial rejection phase. Trials were then visually inspected and those containing artifacts were excluded from subsequent analyses. In this trial rejection phase, we ensured that there were no relevant mouth movements prior to the response, as measured with the EMG. After trial rejection, each participant had, on average, 49.54 (SD = 2.34) epochs for the Fast Responses [range = 43-52] and 48.86 (SD = 2.70) epochs for the Slow Responses [range = 44-53]. The number of epochs for each condition was not significantly different when tested with a t-test [t(20) = 0.73, p = 0.47].

# Source estimation

For the source analysis, Individual T1 MRI scans were segmented by means of the recon-all routine of FreeSurfer image analysis suite, which is documented and freely available for download online (<http://surfer.nmr.mgh.harvard.edu/>). The technical details of these segmentation procedures are described in prior publications55. MRI and MEG data were registered according to the head-coil positions, identified with E-vitamin capsules. From the segmented MRI data, the MEG forward model was calculated with the overlapping spheres method. Source reconstruction was calculated on the cortex surface, constraining the source directions to be orthogonal to surface meshes. Source reconstruction was performed with the wMNE (weighted Minimum Norm) algorithm, using the Brainstorm default settings. The noise covariance, necessary for source estimates using wMNE, was calculated from 3 minutes of empty room recording, made at the end of the recording session for each participant (empty room data followed the same pre-processing steps of regular data). By following Brainstorm procedure, a common kernel for source estimation was calculated taking into account head model (based on individual MRI and head position relative to sensors) and noise covariance calculated from empty room. All analyses were performed at source level.

# Multiplication problems and classification into Fast and Slow Responses

The following table reports the relationship between problem size and the classification in Fast and Slow responses. As expected, more difficult problems were often associated with Slow Responses, whereas easier problems were more often associated with Fast Responses.

| Operation | Fast Response Proportion | Slow Response Proportion | Operation | Fast Response Proportion | Slow Response Proportion |
| --- | --- | --- | --- | --- | --- |
| 2 x 3 | 0.87 | 0.13 | **6 x 2** | 0.96 | 0.04 |
| 2 x 4 | 0.83 | 0.17 | **6 x 3** | 0.60 | 0.40 |
| 2 x 5 | 0.95 | 0.05 | **6 x 4** | 0.39 | 0.61 |
| 2 x 6 | 0.81 | 0.19 | **6 x 5** | 0.31 | 0.69 |
| 2 x 7 | 0.91 | 0.09 | **6 x 7** | 0.17 | 0.83 |
| 2 x 8 | 0.78 | 0.22 | **6 x 8** | 0.33 | 0.67 |
| 2 x 9 | 0.93 | 0.07 | **6 x 9** | 0.00 | 1.00 |
| 3 x 2 | 0.93 | 0.07 | **7 x 2** | 0.90 | 0.10 |
| 3 x 4 | 0.59 | 0.41 | **7 x 3** | 0.70 | 0.30 |
| 3 x 5 | 0.75 | 0.25 | **7 x 4** | 0.14 | 0.86 |
| 3 x 6 | 0.45 | 0.55 | **7 x 5** | 0.52 | 0.48 |
| 3 x 7 | 0.71 | 0.29 | **7 x 6** | 0.09 | 0.91 |
| 3 x 8 | 0.21 | 0.79 | **7 x 8** | 0.27 | 0.73 |
| 3 x 9 | 0.13 | 0.87 | **7 x 9** | 0.00 | 1.00 |
| 4 x 2 | 0.84 | 0.16 | **8 x 2** | 0.69 | 0.31 |
| 4 x 3 | 0.51 | 0.49 | **8 x 3** | 0.31 | 0.69 |
| 4 x 5 | 0.75 | 0.25 | **8 x 4** | 0.05 | 0.95 |
| 4 x 6 | 0.20 | 0.80 | **8 x 5** | 0.53 | 0.47 |
| 4 x 7 | 0.05 | 0.95 | **8 x 6** | 0.13 | 0.87 |
| 4 x 8 | 0.07 | 0.93 | **8 x 7** | 0.23 | 0.77 |
| 4 x 9 | 0.05 | 0.95 | **8 x 9** | 0.11 | 0.89 |
| 5 x 2 | 0.98 | 0.02 | **9 x 2** | 0.95 | 0.05 |
| 5 x 3 | 0.91 | 0.09 | **9 x 3** | 0.53 | 0.48 |
| 5 x 4 | 0.85 | 0.15 | **9 x 4** | 0.00 | 1.00 |
| 5 x 6 | 0.22 | 0.78 | **9 x 5** | 0.13 | 0.87 |
| 5 x 7 | 0.16 | 0.84 | **9 x 6** | 0.03 | 0.97 |
| 5 x 8 | 0.22 | 0.78 | **9 x 7** | 0.06 | 0.94 |
| 5 x 9 | 0.23 | 0.78 | **9 x 8** | 0.10 | 0.90 |

**Table S.2. Proportion of Fast and Slow Responses across different operations.** The table shows all the operations included in the Experiment. For each operation the proportion of overall trials classified as Fast Responses and Slow Responses (across all participants) is reported.

# Details on Response Times across runs

We assessed the change of Response time across runs by means of a repeated measures ANOVA with two within variables: Response  *type* (Fast, Slow) and *Run* (1,2,3). The dependent variable was the Response Time (i.e., the onset of a correct response).

The ANOVA showed a significant effect of *Response Type* [F(1,20) = 567.50, p < 0.001] and significant effect of *Run* [F(2,40) = 8.91, p < 0.001] but no significant interaction of *Response type* and *Run*. The post-hoc t-tests investigating significant effect of Run indicate faster reaction times in Block 3 as compared to Block 2 and Block 1 (FDR corrected post-hocs < 0.05), but no significant different between Block 2 and Block 3 (FDR corrected post-hocs > 0.05). Note that the significant effect of Response Type was trivial, as the Response Type was a dichotomous variable based on RTs. Results are reported in Figure S.1. Detailed values separated for each Subject and Run are reported in Table S.3


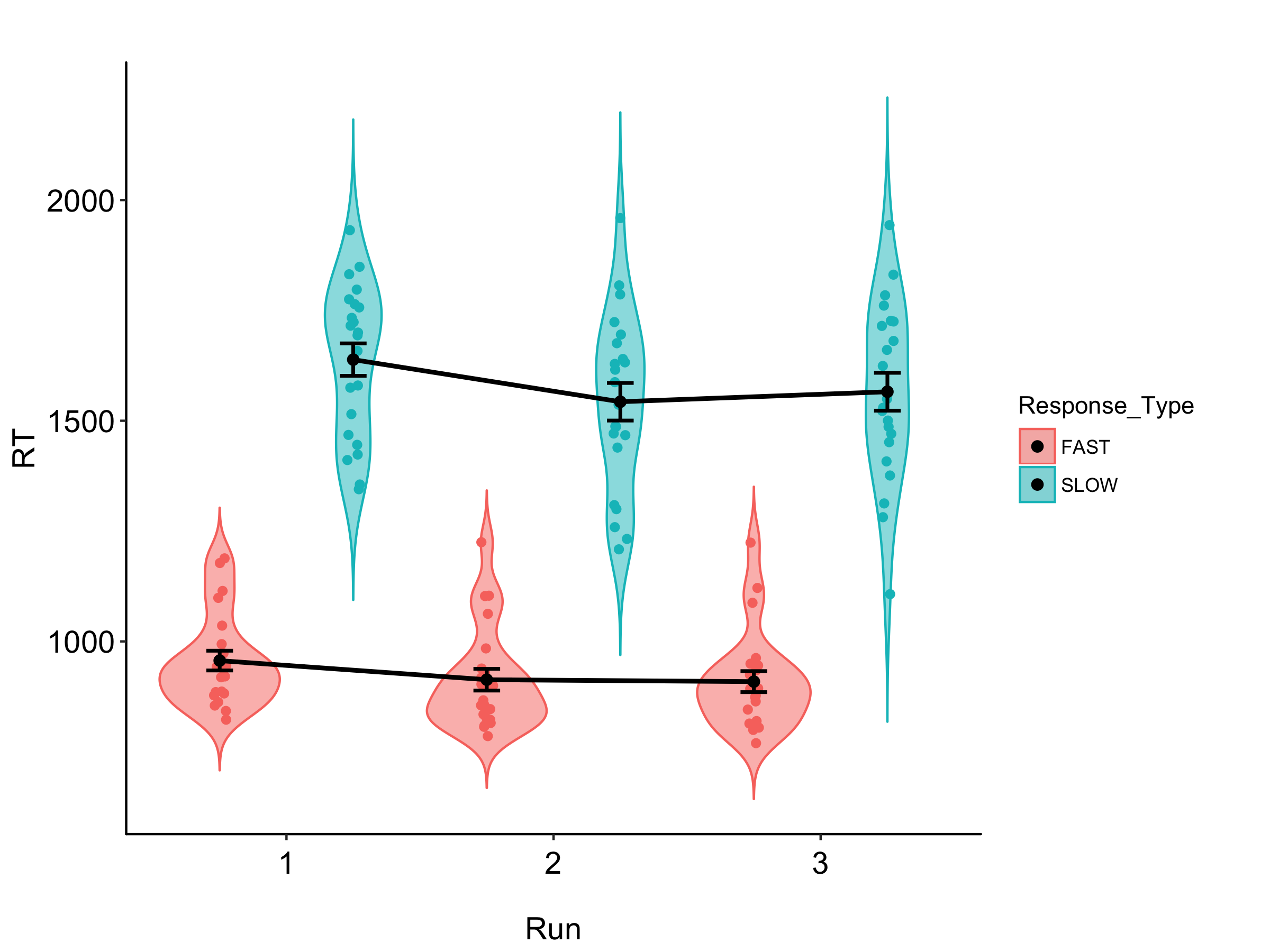


**Figure S.1.1 Mean RT across Runs.** The figure shows the change of mean RTs across runs. The red and green colored points indicate individual performances, while the black points indicate the average within each run.

| Subject | ResponseType | RT Run 1 | RT Run 2 | RT Run 3 |
| --- | --- | --- | --- | --- |
| MH001 | FAST | 823 | 815 | 820 |
| MH001 | SLOW | 1575 | 1487 | 1522 |
| MH002 | FAST | 1178 | 1225 | 1224 |
| MH002 | SLOW | 1832 | 1959 | 1943 |
| MH003 | FAST | 842 | 809 | 814 |
| MH003 | SLOW | 1445 | 1209 | 1281 |
| MH004 | FAST | 886 | 903 | 924 |
| MH004 | SLOW | 1658 | 1807 | 1725 |
| MH005 | FAST | 1036 | 984 | 950 |
| MH005 | SLOW | 1757 | 1640 | 1529 |
| MH006 | FAST | 1099 | 1104 | 1121 |
| MH006 | SLOW | 1775 | 1695 | 1726 |
| MH007 | FAST | 973 | 855 | 935 |
| MH007 | SLOW | 1715 | 1676 | 1681 |
| MH008 | FAST | 944 | 835 | 800 |
| MH008 | SLOW | 1411 | 1232 | 1107 |
| MH009 | FAST | 1188 | 1103 | 1088 |
| MH009 | SLOW | 1733 | 1786 | 1831 |
| MH010 | FAST | 885 | 823 | 805 |
| MH010 | SLOW | 1515 | 1487 | 1486 |
| MH011 | FAST | 855 | 785 | 769 |
| MH011 | SLOW | 1723 | 1471 | 1500 |
| MH012 | FAST | 863 | 807 | 807 |
| MH012 | SLOW | 1423 | 1259 | 1313 |
| MH014 | FAST | 942 | 826 | 864 |
| MH014 | SLOW | 1932 | 1467 | 1761 |
| MH015 | FAST | 937 | 900 | 894 |
| MH015 | SLOW | 1694 | 1587 | 1408 |
| MH016 | FAST | 882 | 867 | 893 |
| MH016 | SLOW | 1468 | 1309 | 1451 |
| MH017 | FAST | 921 | 918 | 898 |
| MH017 | SLOW | 1356 | 1536 | 1549 |
| MH018 | FAST | 945 | 856 | 874 |
| MH018 | SLOW | 1700 | 1616 | 1784 |
| MH019 | FAST | 994 | 930 | 877 |
| MH019 | SLOW | 1580 | 1439 | 1376 |
| MH020 | FAST | 943 | 939 | 945 |
| MH020 | SLOW | 1797 | 1724 | 1661 |
| MH021 | FAST | 1114 | 1063 | 962 |
| MH021 | SLOW | 1849 | 1629 | 1624 |
| MH022 | FAST | 878 | 847 | 846 |
| MH022 | SLOW | 1345 | 1300 | 1470 |

**Table S.3 RT across run.** The table reports average RTs separated by Subjects , Run and Response type.

**
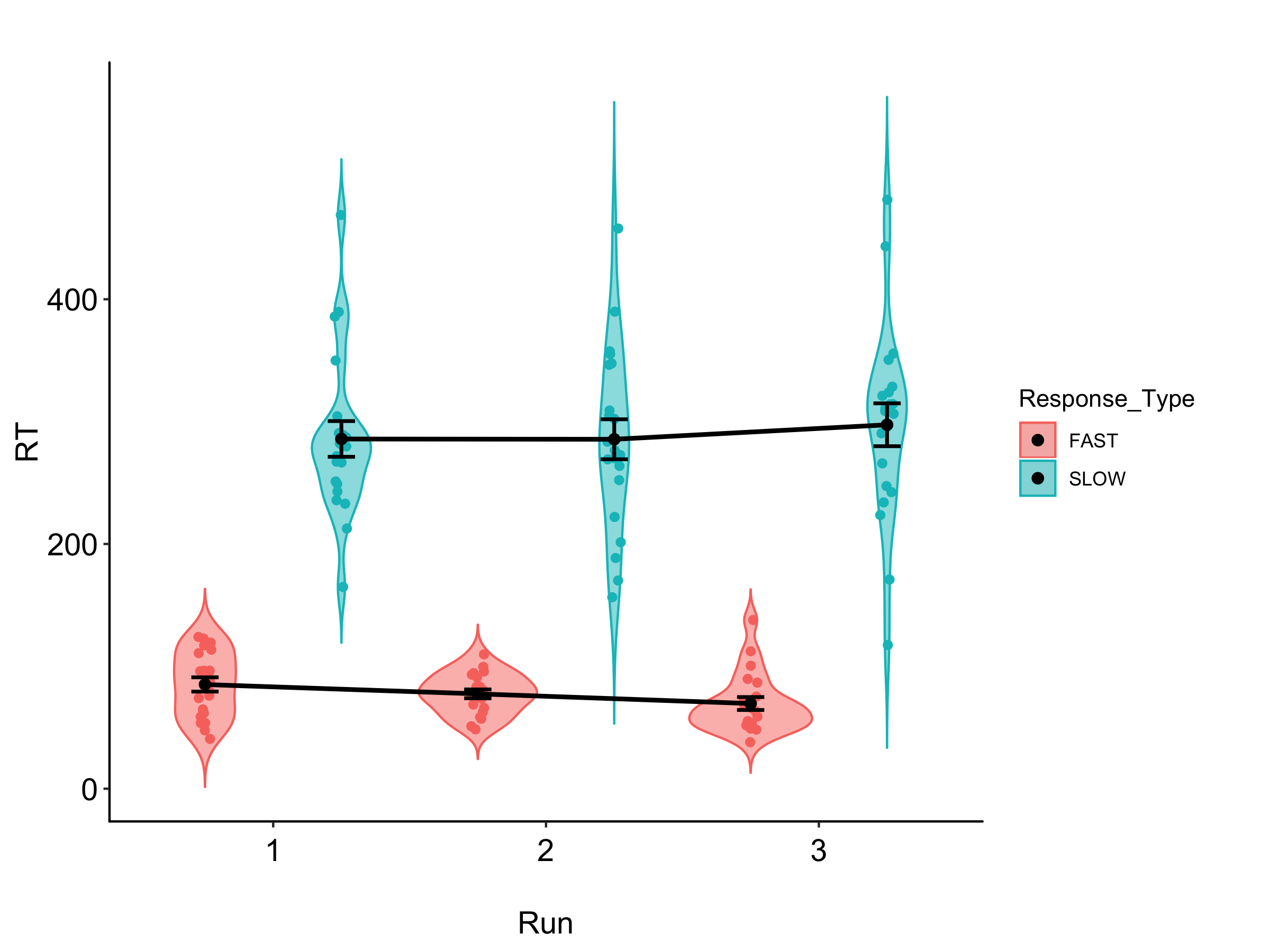
**

**Figure S.1.2 Standard Deviation of RTs across Runs.** The figure shows the change of standard deviation of RTs across runs. The red and green colored points correspond to single participants, while the black points indicate the average of participants’ SD within each run

**Correlations between source activity**  **and Response Time**

The following table reports the significant correlations between source activity and response times for uncorrected p-values.

The full results are available as .Rdata object at the present permalink of the Open Science Framework site <https://osf.io/b32xy/>.

| interval | ROI | r | df | Response | p | p FDR |
| --- | --- | --- | --- | --- | --- | --- |
| 100-200 | Right Supramarginal Gyrus | 0.50 | 19 | Fast | 0.02 | 0.99 |
| 0-100 | Left Angular Gyrus | -0.55 1 | 19 | Slow | 0.009 | 0.99 |

# Time Frequency Plots

The following pages present some additional figures for a better inspection of Time Frequency plots. The files used to generate this figures are available from this OSF permalink <https://osf.io/b32xy/> in the form of a Brainstorm protocol. By using the Brainstorm software (Tadel, Baillet, Mosher, Pantazis, & Leahy, 2011) it is possible to interactively explore the results.

## Time Frequency plot - Left Angular Gyrus


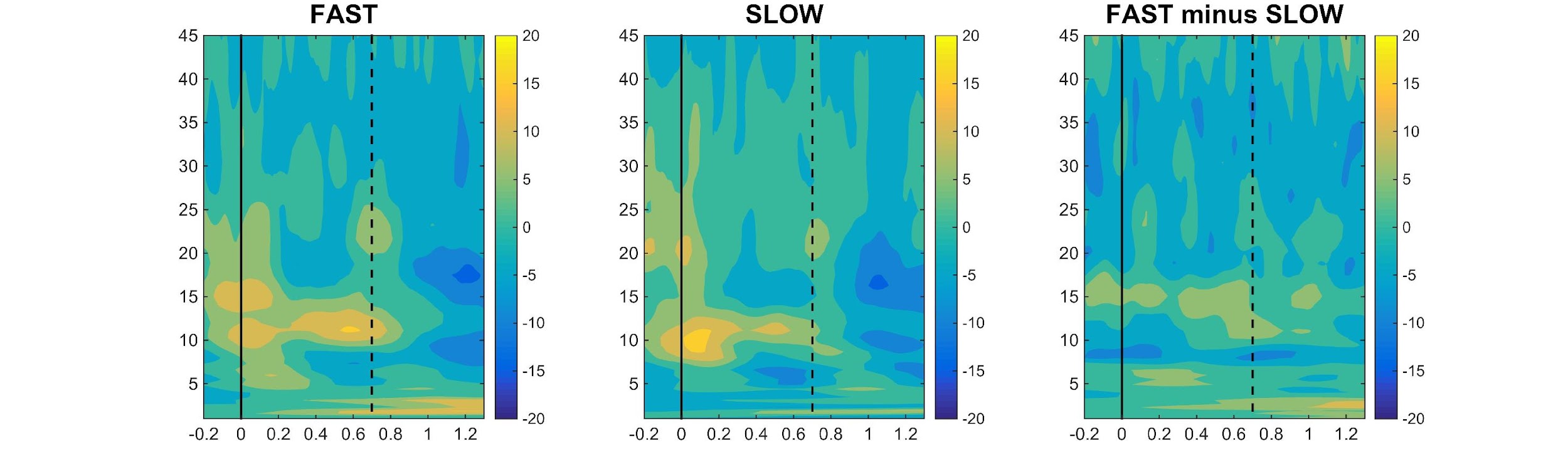


**Figure S.2 Time Frequency plot of Left Angular Gyrus.** The figure shows the results of the Time-Frequency analysis based on Morlet deconvolution, comparing *Fast Responses* and *Slow Responses*. The first two panels (from the left) show the average response for Fast and Slow responses of the ERS/ERD change relative to a baseline window of -500 -3 00 ms. The third panel shows the difference Fast minus Slow. The solid black line indicates the presentation of the First number, while the dashed black line indicates the presentation of the second number.

## Time Frequency plot - Right Angular Gyrus


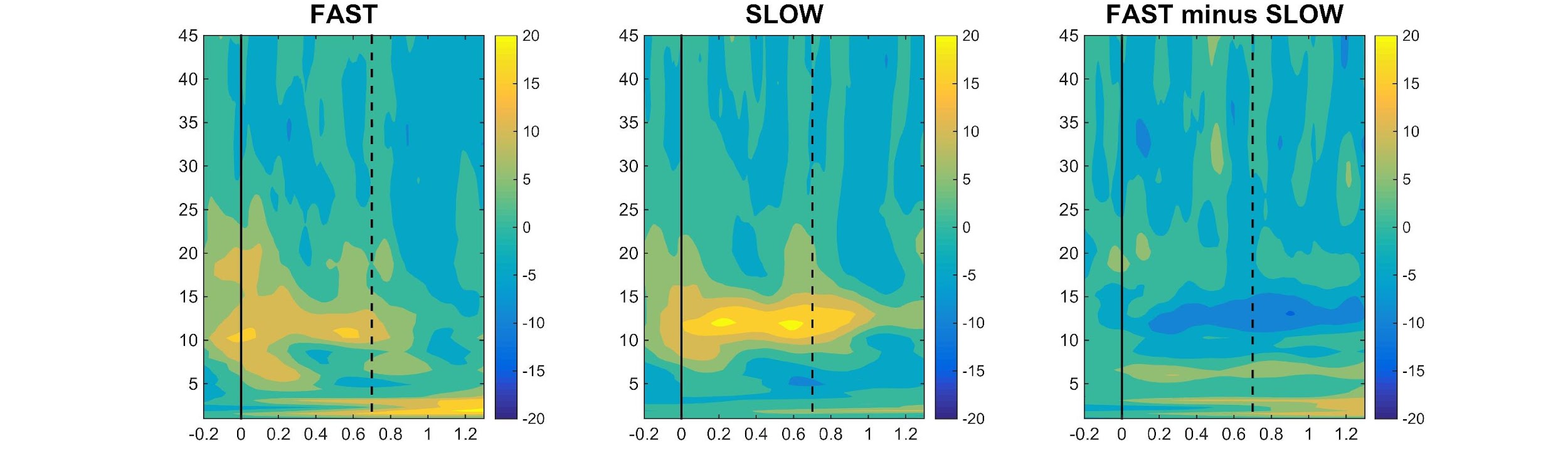


**Figure S.3 Time Frequency plot of Right Angular Gyrus.** The figure shows the results of the Time-Frequency analysis based on Morlet deconvolution, comparing *Fast Responses* and *Slow Responses*. The first two panels (from the left) show the average response for Fast and Slow responses of the ERS/ERD change relative to a baseline window of -500 -3 00 ms. The third panel shows the difference Fast minus Slow. The solid black line indicates the presentation of the First number, while the dashed black line indicates the presentation of the second number.

## Time Frequency plot - Left Supramarginal Gyrus


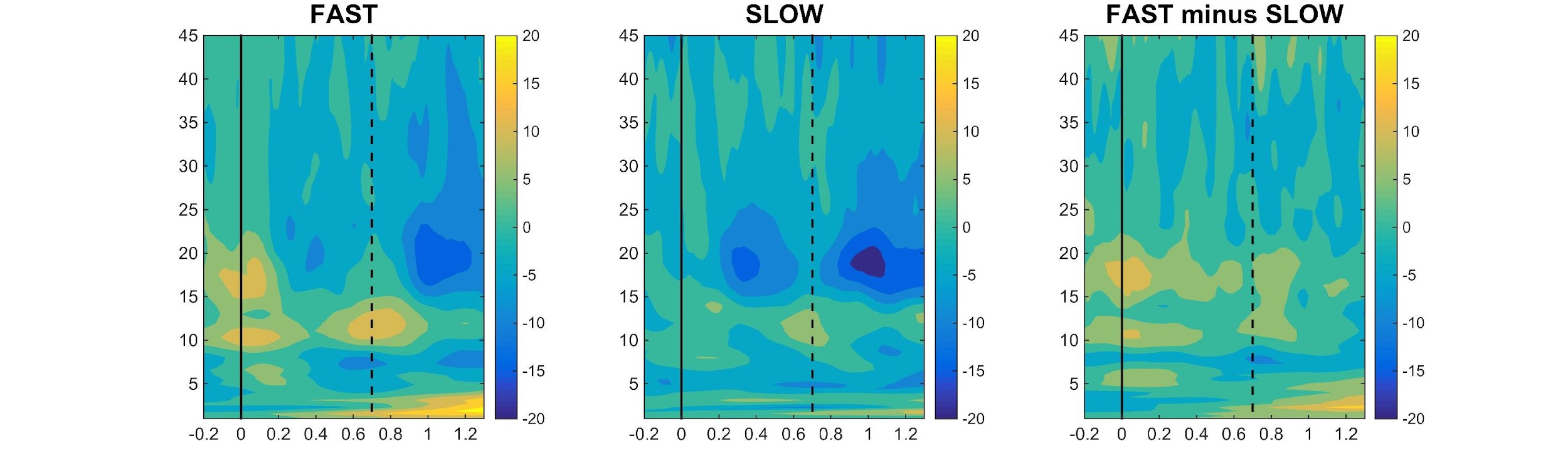


**Figure S.4 Time Frequency plots of Left Supramarginal Gyrus.** The figure shows the results of the Time-Frequency analysis based on Morlet deconvolution, comparing *Fast Responses* and *Slow Responses*. The first two panels (from the left) show the average response for Fast and Slow responses of the ERS/ERD change relative to a baseline window of -500 -3 00 ms. The third panel shows the difference Fast minus Slow. The solid black line indicates the presentation of the First number, while the dashed black line indicates the presentation of the second number.

## Time Frequency plot - Right Supramarginal Gyrus


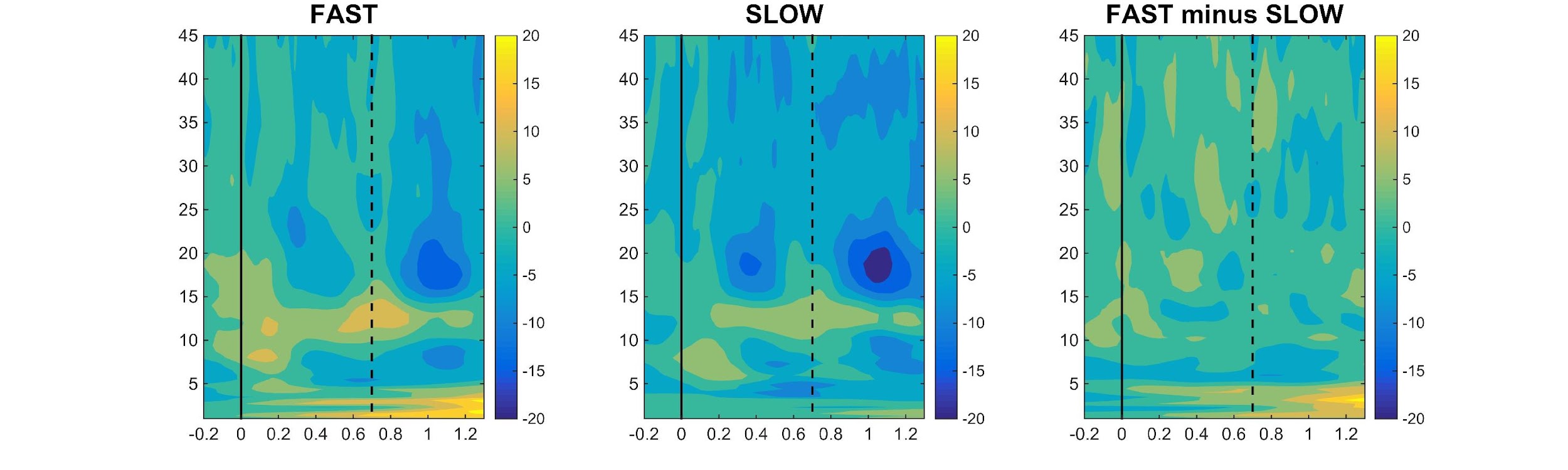


**Figure S.5 Time Frequency plots of Right Supramarginal Gyrus.** The figure shows the results of the Time-Frequency analysis based on Morlet deconvolution, comparing *Fast Responses* and *Slow Responses*. The first two panels (from the left) show the average response for Fast and Slow responses of the ERS/ERD change relative to a baseline window of -500 -3 00 ms. The third panel shows the difference Fast minus Slow. The solid black line indicates the presentation of the First number, while the dashed black line indicates the presentation of the second number.

**
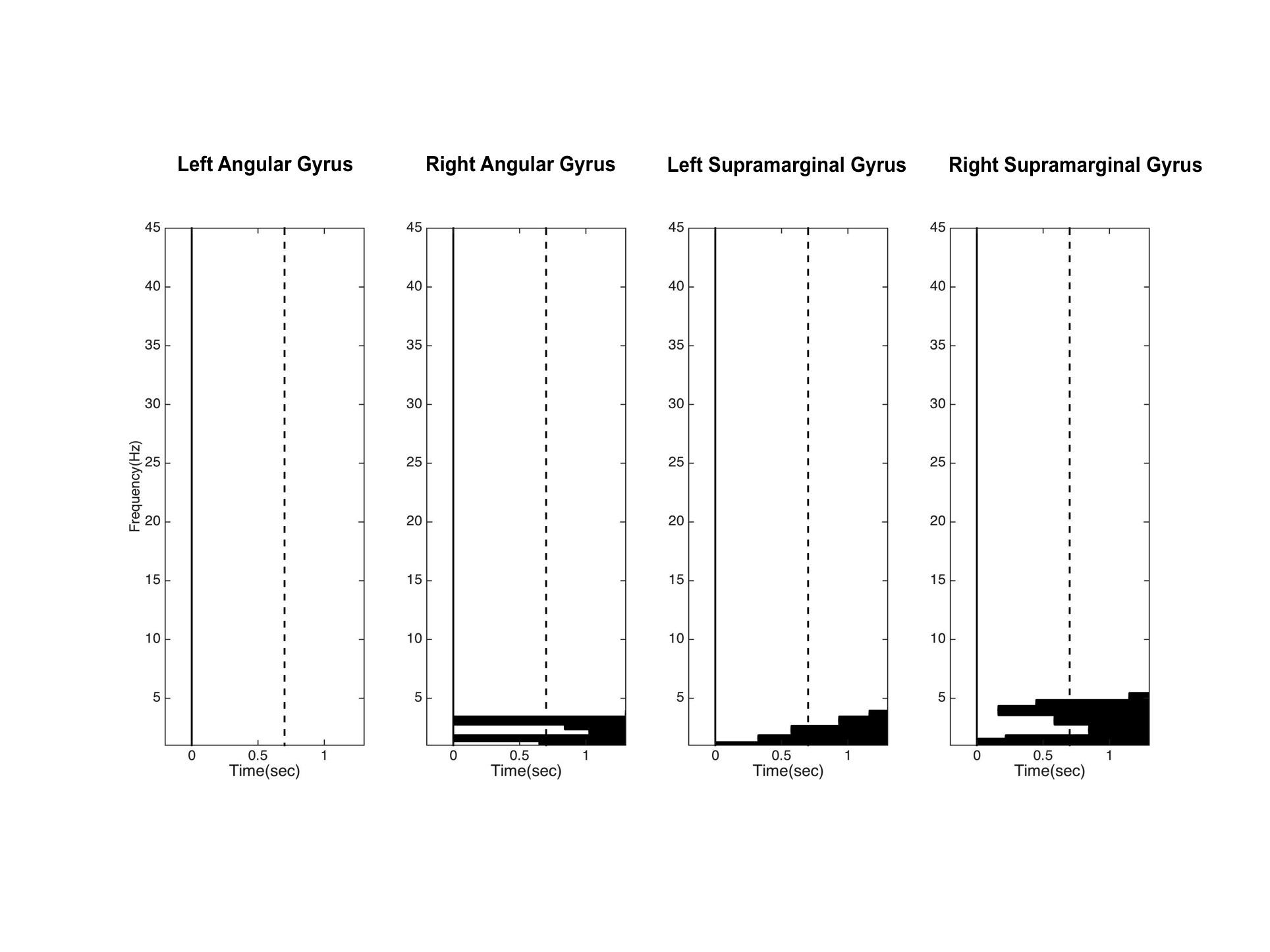
**

**Figure S.6 Highlights of statistically significant results of Time Frequency Analysis**. The figure shows the significant differences of the cluster-based permutation performed for Time Frequency Analysis reported in the manuscript (p = 0.05). The black areas denote the significant clusters, in which *Fast Responses* had higher values than *Slow Responses*. The present figure is a larger version of the bottom panel of Figure 4 in the Manuscript.

# Additional Time Frequency analyses

This section reports some additional analyses to investigate the robustness of Time Frequency Results.

## Magnitude data distributions

Data distribution of Average Time Frequency responses used in the analysis.


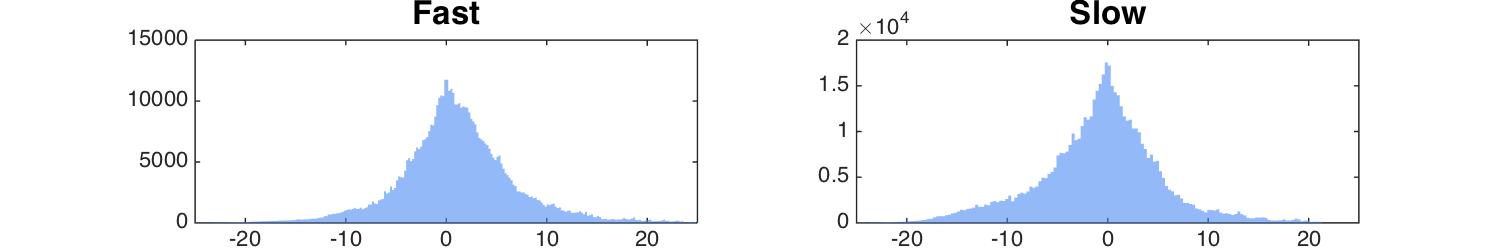


**Figure S7.1 Data Distribution of average Magnitude data.** The two panels report, separately for Fast and Slow Response, the data distribution of Magnitude Values used for the Time Frequency analysis included in the manuscript.

Data showed a symmetrical distribution, with a slight leptokurtosis as compared to normal distribution.

## Statistical results of Time Frequency analysis removing Evoked activity

Time Frequency analysis allows to capture time-locked responses which are not necessarily phase-locked. On the other hand, ERF allows capturing phase-locked responses. With these premises, part of the Time Frequency results could be highly overlapped with results derived by ERF. In particular, Time Frequency responses on low frequency windows (in the delta/theta range) could be highly related to the response obtained with ERF.

To provide a more complete picture we report here statistical analysis of Time Frequency data after removing the contribution of the phase-locked response. This is made by running a time frequency analysis removing, by each epoch, the average of all epochs (that is the ERF, or evoked response). The analysis showed statistically significant results, indicating that most of the observed results were overlapped to those of source activations (obtained from ERFs).


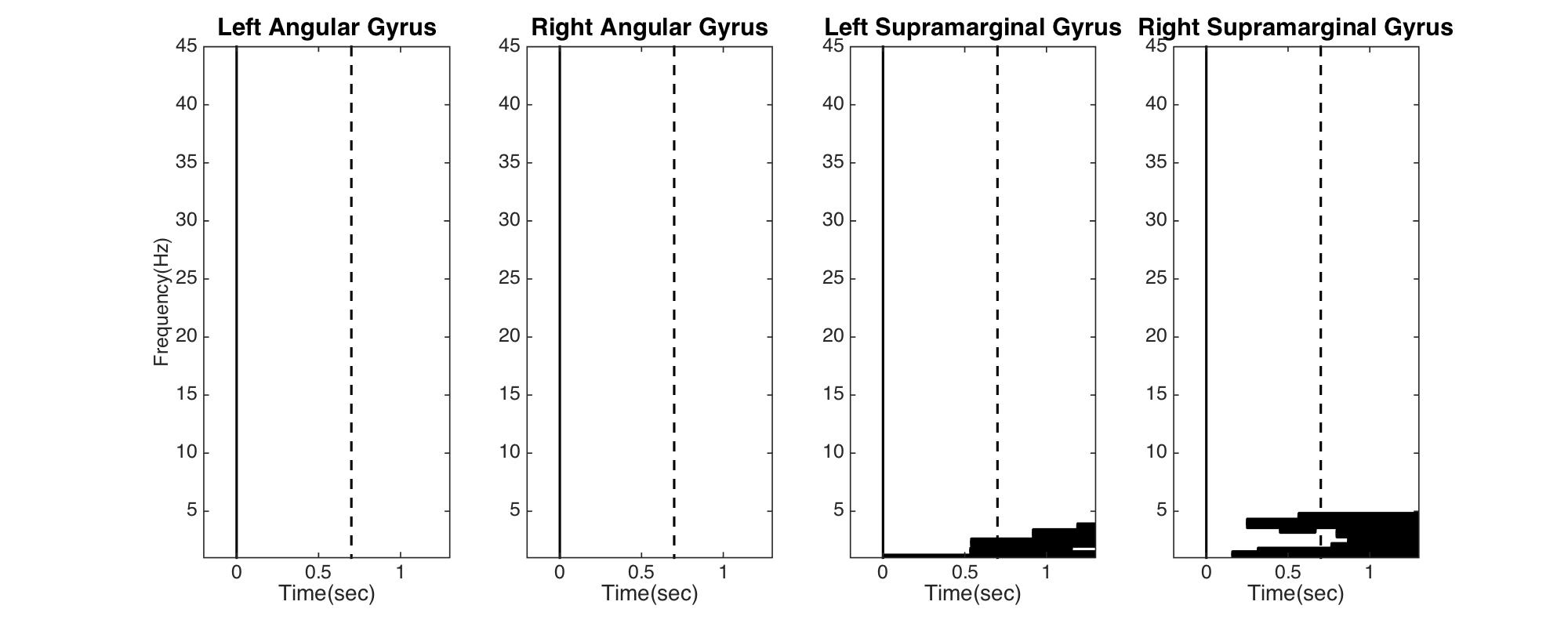


**Figure S.7.2 Highlights of statistically significant results of Time Frequency Analysis after removing the evoked response**. The figure shows the significant differences of the cluster-based permutation performed for Time Frequency Analysis after removing the evoked response. The black areas denote the significant clusters, in which *Fast Responses* had higher values than *Slow Responses*.

##

##

## Statistical results of Time Frequency analysis using Power

To investigate whether the observed results were related to the choice of the Time Frequency measure (we opted for Magnitude), we also re-run the analyses another common measure employed in TF analysis, i.e. Power (Power is calculated as Magnitude^2^).

The exact same pre-processing steps were performed on the data. Statistical results using Power showed a remarkable similarity to the ones obtained with Magnitude, confirming the robustness of the findings (see figure S6).


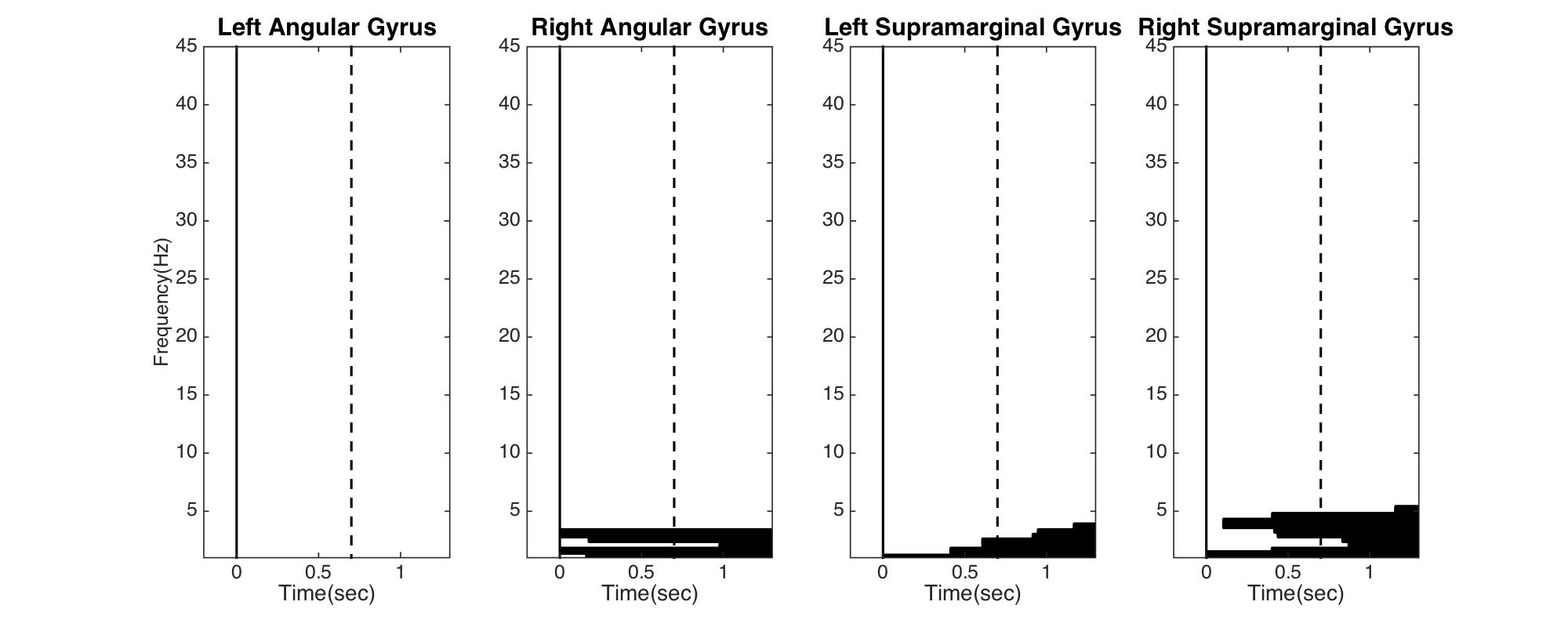


**Figure S.7.3 Highlights of statistically significant results of Time Frequency Analysis using Power**. The figure shows the significant differences of the cluster-based permutation performed for Time Frequency Analysis using Power as measure. The black areas denote the significant clusters, in which *Fast Responses* had higher values than *Slow Responses* (See figure S6 for comparison to results using Magnitude).

# Time course of signal variability

A potential issue of results of the present study is related to the potential systematic variability in the signal across conditions, which could be a bias for the statistical analysis. To shed light on this potential issue, we performed additional analyses to investigate if the two different conditions differed not only in average activity (as shown in the main manuscript), but also in variability.

**Source activity**

Concerning source activity, we investigated whether there was a systematic increase in variability over time. We performed the same pre-processing steps as for the average source activations, but using standard deviations in place of average as summary measure for each participant. The figures below show the average change in variability (in standard deviation). A slight increase in standard deviation can be observed over time, but this does not appear to be related to the time windows in which significant effects were found.


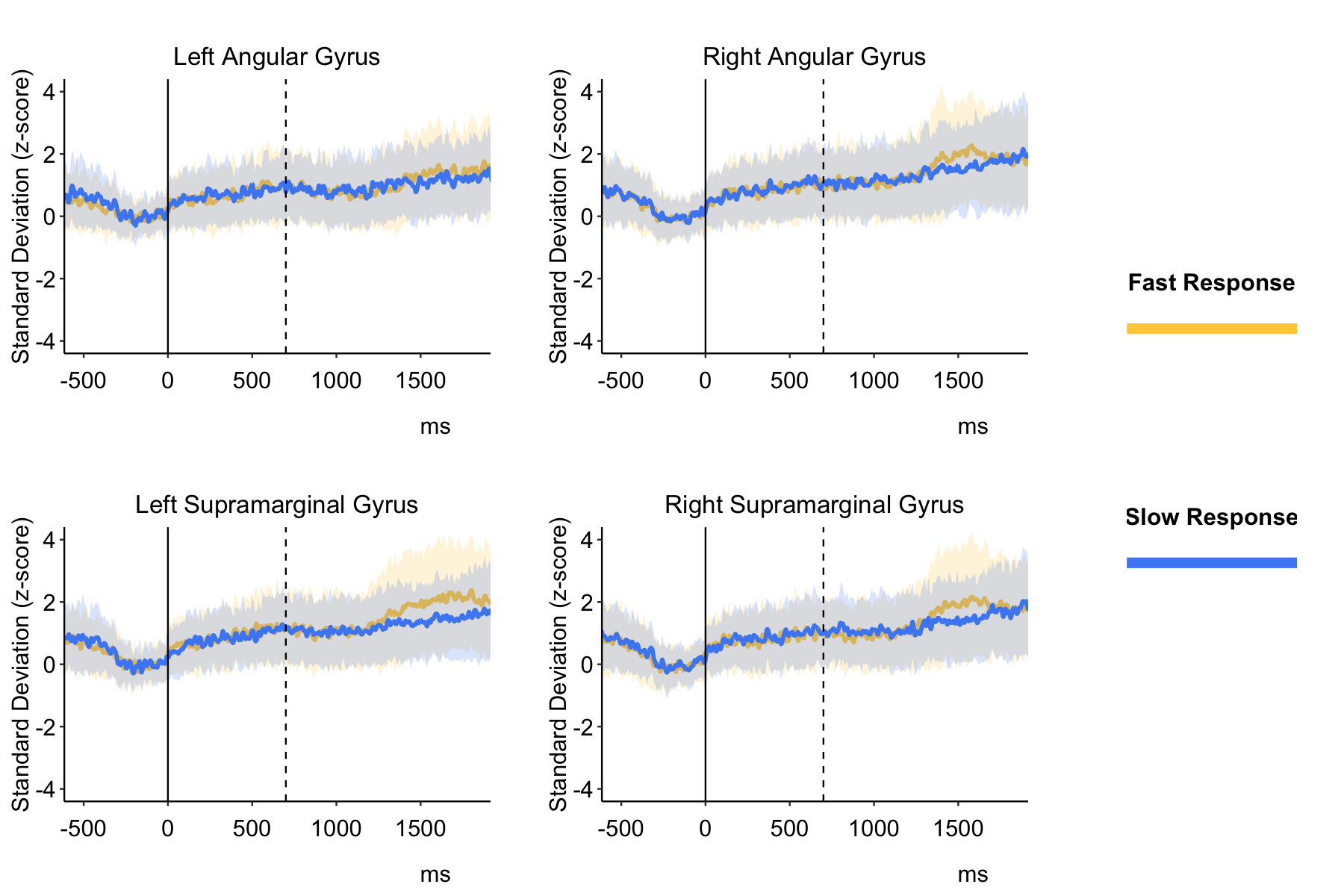


**Figure S8.1 Time course of the standard deviation of source activations**

This is confirmed by the plot showing differentials (Slow minus Fast, see Figure S.8.2), in which the data showed clearly that standard deviation changes (as compared to baseline) were similar across conditions. Importantly a difference is instead observed in the time window in which the oral response was present, with higher variability for Fast as compared to Slow response. This is expected given the artifact of oral responses in earlier time windows for Fast responses, but note the time window showing this difference was not analyzed in the manuscript. These results shed light on one of the starting hypotheses of our papers, which suggested that slow responses could show lower activation values, due to increased variability. This appears not to be the case, as changes in variability seem very similar between Fast and Slow responses. Hence, this result can be tentatively interpreted as pointing to an overall reduced activation of the areas evidenced in the analyses in Slow as compared to Fast responses.


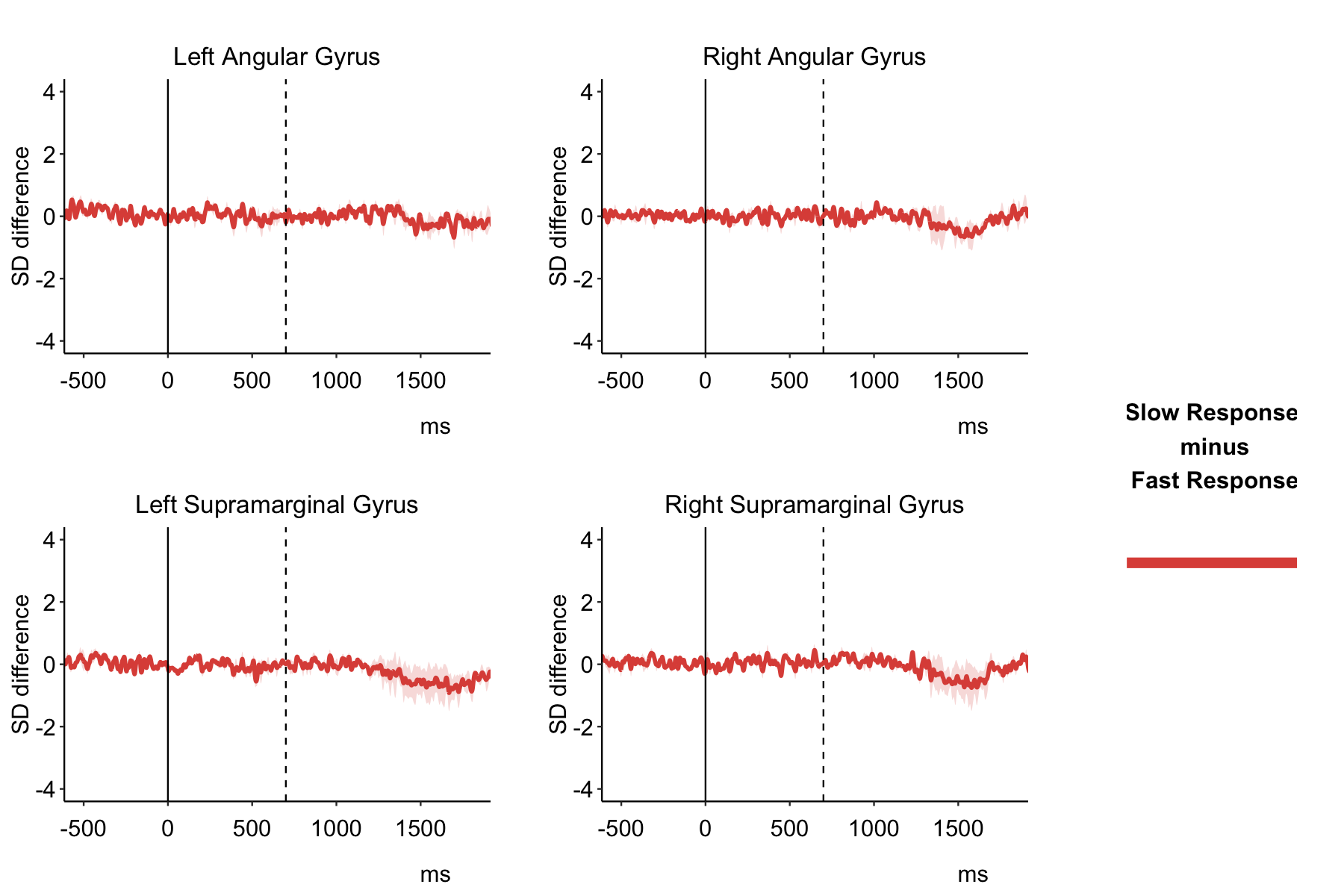


**Figure S8.2 Time course of the standard deviation of source activations**

**Time Frequency activity**

Concerning Time Frequency activity, we adopted a similar approach to the one described above for Source activity. In the case of TF responses, systematic differences were more pronounced and similar to the observed effects. For this reason, we further explored this potential bias performing a cluster permutation analysis, which showed a significant difference in standard deviation of the signal for the Right Supramarginal Gyrus, mostly expressed in frequencies from 1 to 3.5 Hz.


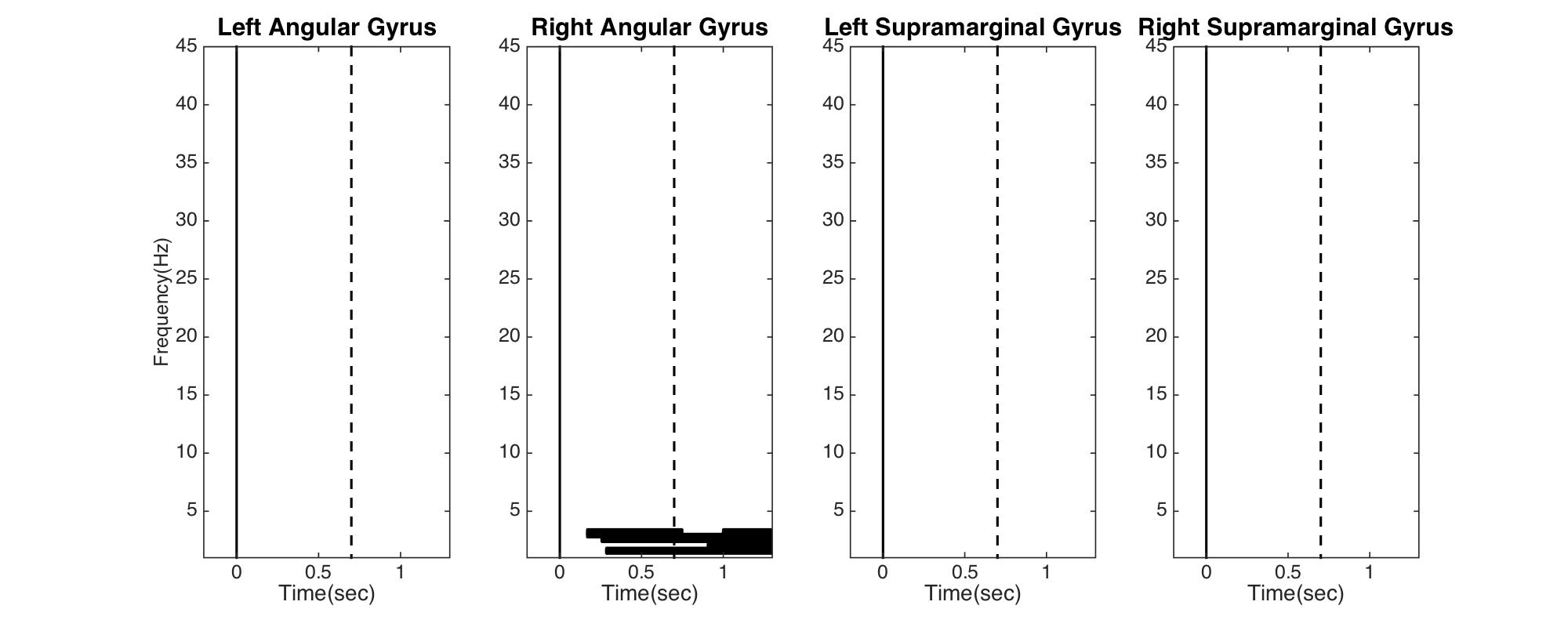


**Figure S8.3 Time**

This result points to potential bias of signal variability on statistical analysis, but it does not necessarily indicate that there is a specific bias in the present study, but could be related to mathematical transformation applied (see below). These results pointed to the need for further investigations, which we reported below.

**Further explorations**

As further exploration we perform a simulation generating synthetic EEG. Evoked signal of this EEG data was simulated as part of a sine wave with given frequency in a fixed time window (see figure below). For sake of simplicity noise was simulated as white noise, and no jitter was introduced in the data.
A sample of the generated trials is reported in figure S.8.4


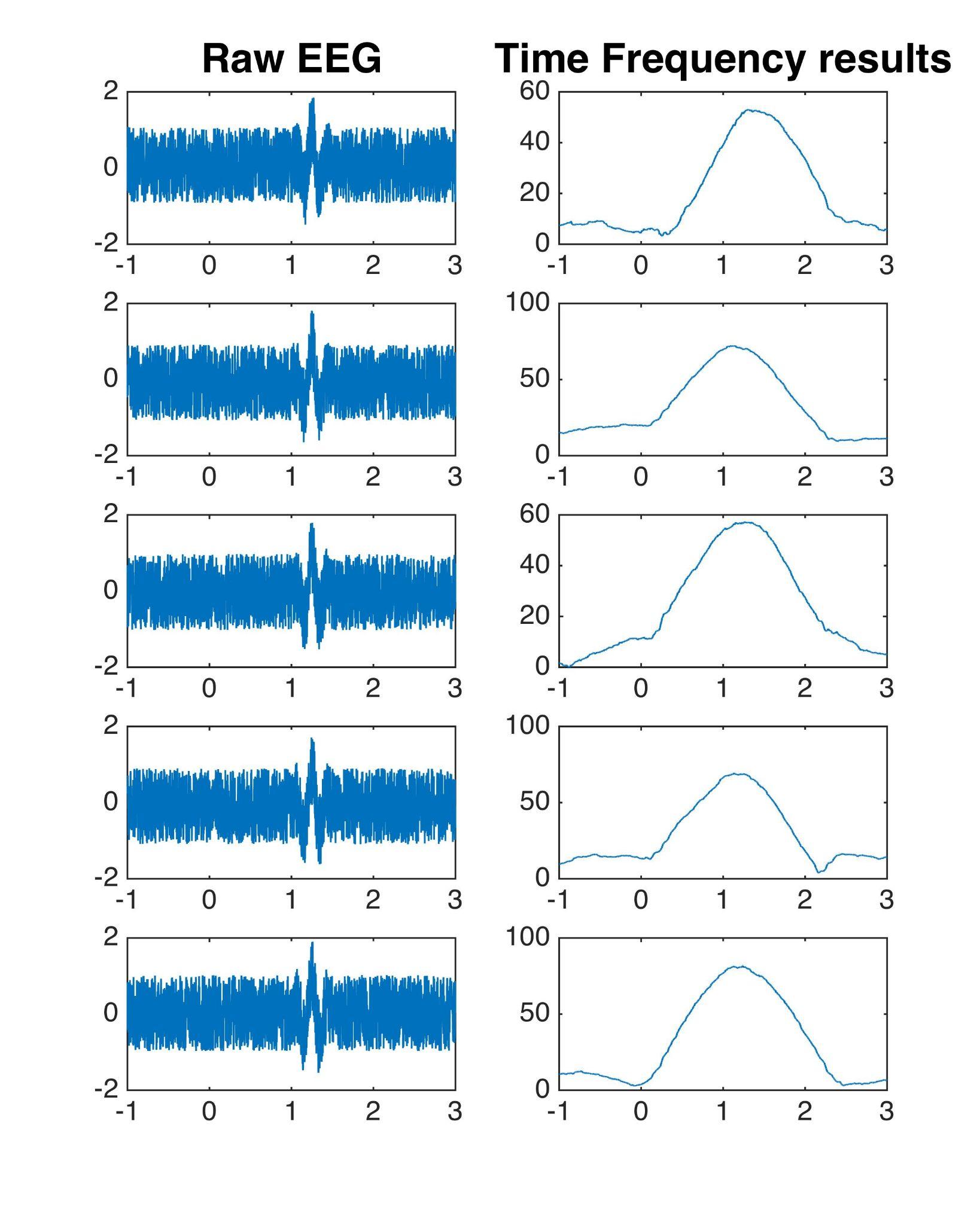


**Figure S.8.4 Sample of simulated trials**

We then calculated the average and the standard deviation of these raw trials. In Figure X.1. you can see no relationship between the ERP and the noise. Similar results, as expected, were found after calculating the z-score. These results are analogous to what we found for source activity analysis in the manuscript. See figure S.8.5


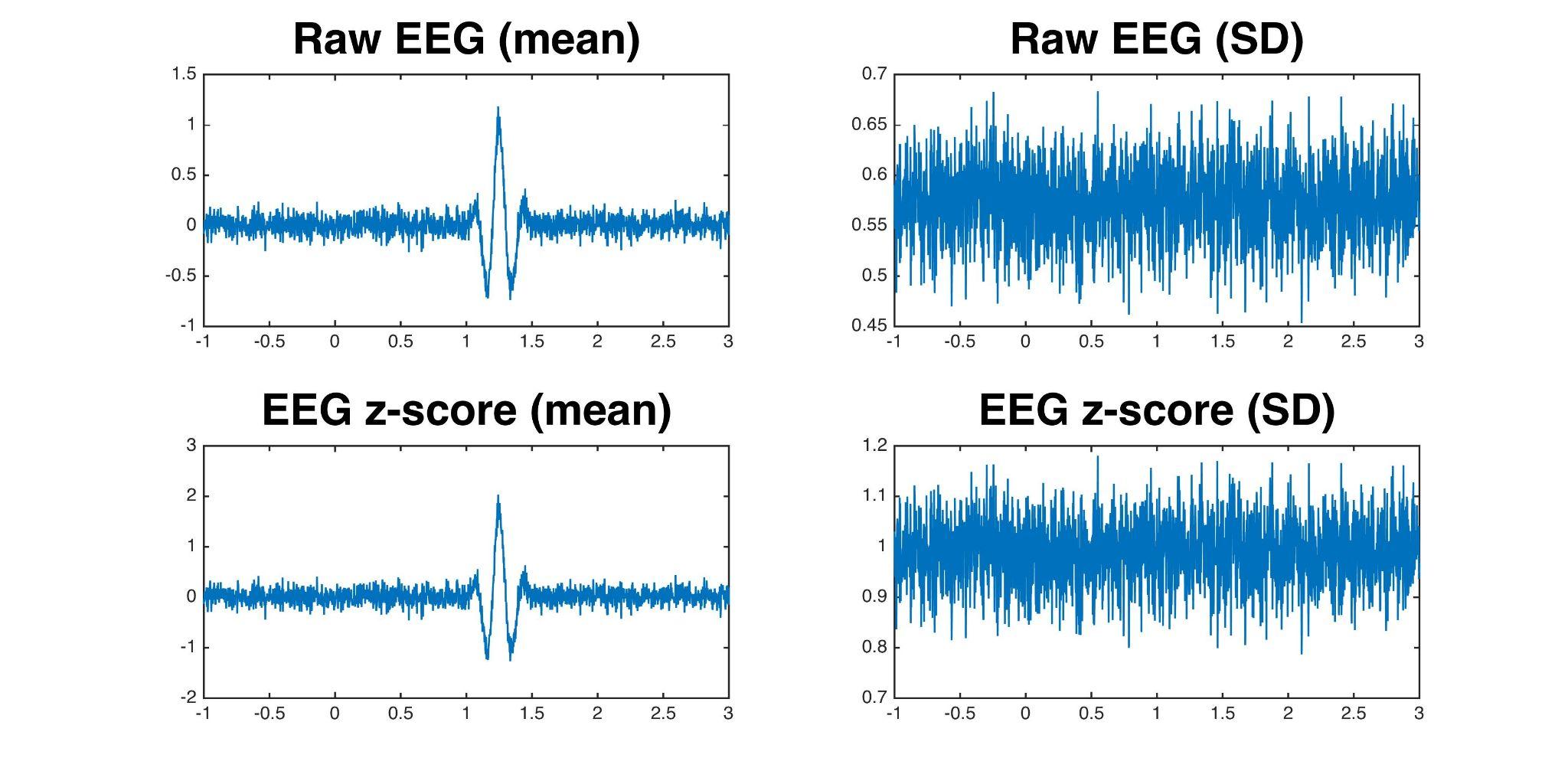


**Figure S.8.5 Mean and SD of Amplitude values simulated trials.** The figure shows the average of EEG (raw and after z-score transformation), and the average of their standard deviations (SD).

However, when, performing Time Frequency analysis on the same simulated data, both using Magnitude or calculating the ERS/ERD (see Figure S.8.6), it is possible to appreciate a systematic change in both signal amplitude and in signal variability, which are then related to the applied mathematical transformation, rather than to signal properties. This result is not surprising as the aim of Time Frequency analysis is to isolate the modulation in a specific frequency as compared to the background noise. Further considerations and implications of these results goes beyond the aim of the present manuscript, but underlines the complexity of the relationship between signal amplitude and variability in Time Frequency analysis, which may affect, in turn, statistical analyses.


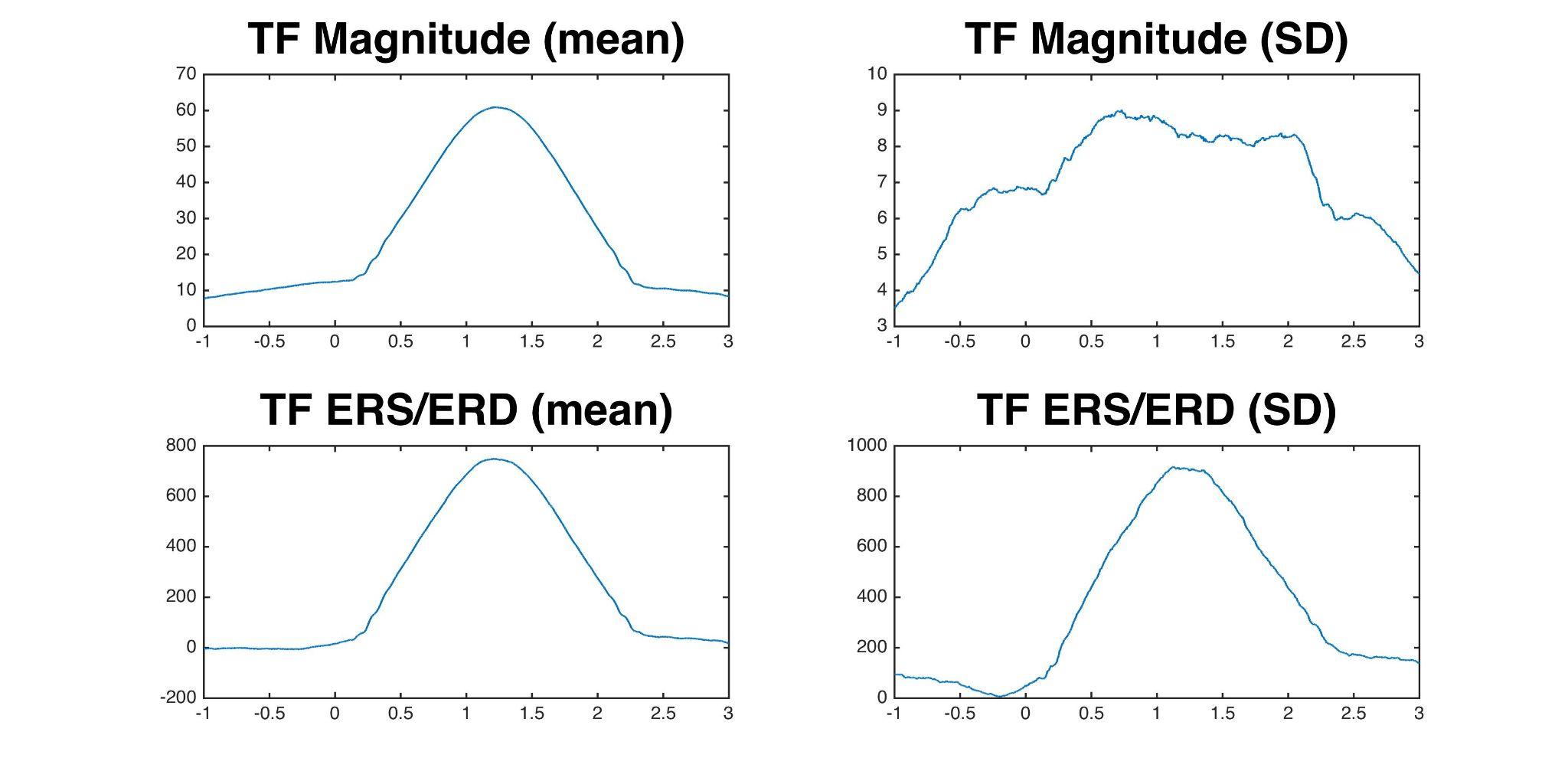


**Figure S.8.6 Mean and SD of Amplitude values simulated trials.** The figure shows the average of Magnitude Value (raw and after ERS/ERD transformation), and the average of their standard deviations (SD).

# Correlation of inversion kernel weights

A potential issue in the similarity across areas is that they may reflect some consequences of the inversion method (that is on the source estimation application) rather than on similarity of data. This is because the estimate for each vertices, is a weighted combination of the signal from sensors. If weights for two ROis are similar, then the similarity across signals of ROIs could be a mere consequence of this aspect.

To empirically calculate the similarity in weights across ROIs in our data we used the following approach:

We started from the inversion kernel which is a matrix with weights with dimensions nVertices x nSensors used for source estimation. Note that the MEG recordings of the subjects did not enter in the analysis, as the inversion kernel was calculated only on the head geometry and the noise covariance, which was calculated from empty room recordings (for sake of simplicity, we focused on the inversion kernel calculated for the first run for each subject) From this matrix, we averaged the weights of all vertices composing each ROI, in order to have a single map of weights for each ROI. Importantly, this step has a major drawback: the weights may depend on the geometrical properties of the vertices (e.g., in the more extreme case, two vertices of the same ROI, if in opposite directions with respect to a sensor, could show equal but opposite weights). Hence these results should be taken with caution. The development of a more defined method to allow comparison of weights in such cases (of ROIs with different numbers of vertices) goes beyond the goal of this exploratory analysis and this paper. We calculated the correlation of the average weights for each ROI, obtaining a correlation matrix for each participant. In summary, this resulting correlation matrix (with dimensions nROIs x nROIs) showed the similarity of average weights across ROIs. We finally averaged all the correlation matrices of subjects to obtain the Figure S9 reported below. The highest non-diagonal correlation value was 0.24, shown between Right Supramarginal and Right Angular Gyrus. This initial analysis shows scarce similarity of weights across ROIs.

*
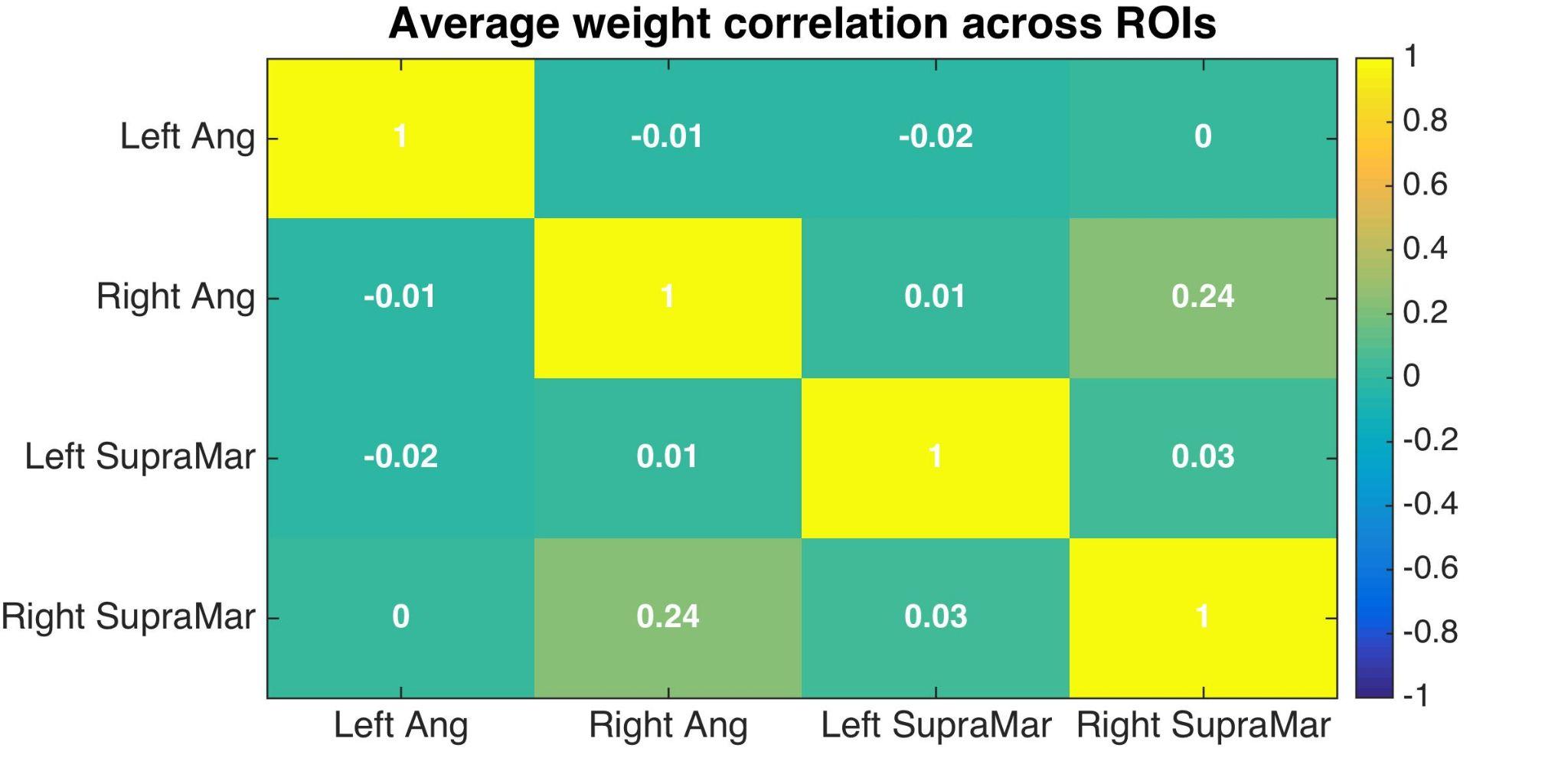
*

Figure S.9 **Correlation of average of weights of the ROIs included in the analysis**

# Details on ANOVAs presented in the main manuscript

The following tables contain all the effects and post-hocs related to the ANOVAs on ROI analysis presented in the manuscript.

A table with all the significant terms (after a further FDR correction using the p-values from all the terms in the ANOVA) is reported below.

In the case of significant interactions in the ANOVA, only the post-hocs for the higher order interactions are reported.

For post-hocs, all p-values reported in the manuscript were also corrected according to False Discovery Rate method (FDR, Benjamini & Hochberg, 1995), separately for each group of post-hocs..

Each table includes seven columns: the first reports the statistical contrast performed, the second the t-value, the third the degrees of freedom of the test, the fourth the p-value, the fifth reports an asterisk (‘*’) if the p-value is below 0.05, the sixth reports the mean value of z-scores of the first term, the seventh column reports the mean value of z-scores of the second term .

In the post-hoc tables, the first column reports the pairwise contrast, the second column reports thet-value, the third column reports the p-value, corrected with FDR method, the fourth column reports the mean of the first term, and the fifth column reports the mean of the second term.

Benjamini, Y., & Hochberg, Y. (1995). Controlling the False Discovery Rate: A Practical And Powerful Approach to Mu. *Journal of the Royal Statistical Society. Series B (Methodological)*, *57*(1), 289–300. Retrieved from http://www.jstor.org/stable/2346101

Tadel, F., Baillet, S., Mosher, J. C., Pantazis, D., & Leahy, R. M. (2011). Brainstorm: A user-friendly application for MEG/EEG analysis. *Computational Intelligence and Neuroscience*, *2011*. http://doi.org/10.1155/2011/879716

## ANOVA - Left Angular Gyrus

**ANOVA**

| Effect | DFn | DFd | F | p | p<.05 | ges |
| --- | --- | --- | --- | --- | --- | --- |
| Response Type | 1 | 20 | 9.74 | 0.005 | * | 0.03 |
| Time Interval | 12 | 240 | 23.55 | < 0.001 | * | 0.32 |
| Response Type x Time Interval | 12 | 240 | 2.44 | 0.005 | * | 0.01 |

**MAUCHLY’S TEST**

| Effect | W | p | p<.05 |  |  |  |
| --- | --- | --- | --- | --- | --- | --- |
| Time Interval | < 0.01 | < 0.001 | * |  |  |  |
| Response Type x Time Interval | < 0.01 | < 0.001 | * |  |  |  |

**SPHERICITY CORRECTIONS**

| Effect | GGe | p[GG] | p[GG]<.05 | HFe | p[HF] | p[HF]<.05 |
| --- | --- | --- | --- | --- | --- | --- |
| Time Interval | 0.28 | < 0.001 | * | 0.35 | < 0.001 | * |
| Response Type x Time Interval | 0.25 | 0.073 |  | 0.3 | 0.061 |  |

## Post-hoc Left Angular Gyrus – Response Type

| contr | p.value | t.value | df | mean.1 | mean.2 | condition_1 | condition_2 |
| --- | --- | --- | --- | --- | --- | --- | --- |
| Fast vs Slow | 0.005 | 3.12 | 20 | 1.63 | 1.34 | Fast | Slow |

## Post-hoc Left Angular Gyrus – Time Interval

| contrast | p.value | | t.value | | df | | mean.1 | | mean.2 | | interval_1 | | interval_2 | |
| --- | --- | --- | --- | --- | --- | --- | --- | --- | --- | --- | --- | --- | --- | --- |
| 0-100 vs 100-200 | < 0.001 | -7.21 | | 20 | | 0.29 | | 0.94 | | 0-100 | | 100-200 | |  |
| 0-100 vs 1000-1100 | < 0.001 | | -10.44 | | 20 | | 0.29 | | 2.13 | | 0-100 | | 1000-1100 | |
| 0-100 vs 1100-1200 | < 0.001 | | -7.93 | | 20 | | 0.29 | | 2.25 | | 0-100 | | 1100-1200 | |
| 0-100 vs 1200-1300 | < 0.001 | | -7.9 | | 20 | | 0.29 | | 2.23 | | 0-100 | | 1200-1300 | |
| 0-100 vs 200-300 | < 0.001 | | -6.58 | | 20 | | 0.29 | | 1.43 | | 0-100 | | 200-300 | |
| 0-100 vs 300-400 | < 0.001 | | -7.14 | | 20 | | 0.29 | | 1.28 | | 0-100 | | 300-400 | |
| 0-100 vs 400-500 | < 0.001 | | -6.37 | | 20 | | 0.29 | | 1.05 | | 0-100 | | 400-500 | |
| 0-100 vs 500-600 | < 0.001 | | -7.29 | | 20 | | 0.29 | | 1.25 | | 0-100 | | 500-600 | |
| 0-100 vs 600-700 | < 0.001 | | -6.26 | | 20 | | 0.29 | | 1.19 | | 0-100 | | 600-700 | |
| 0-100 vs 700-800 | < 0.001 | | -8.35 | | 20 | | 0.29 | | 1.22 | | 0-100 | | 700-800 | |
| 0-100 vs 800-900 | < 0.001 | | -8.39 | | 20 | | 0.29 | | 1.88 | | 0-100 | | 800-900 | |
| 0-100 vs 900-1000 | < 0.001 | | -9.19 | | 20 | | 0.29 | | 2.19 | | 0-100 | | 900-1000 | |
| 100-200 vs 1000-1100 | < 0.001 | | -8.23 | | 20 | | 0.94 | | 2.13 | | 100-200 | | 1000-1100 | |
| 100-200 vs 1100-1200 | < 0.001 | | -5.97 | | 20 | | 0.94 | | 2.25 | | 100-200 | | 1100-1200 | |
| 100-200 vs 1200-1300 | < 0.001 | | -5.45 | | 20 | | 0.94 | | 2.23 | | 100-200 | | 1200-1300 | |
| 100-200 vs 200-300 | 0.001 | | -4.07 | | 20 | | 0.94 | | 1.43 | | 100-200 | | 200-300 | |
| 100-200 vs 300-400 | 0.005 | | -3.14 | | 20 | | 0.94 | | 1.28 | | 100-200 | | 300-400 | |
| 100-200 vs 400-500 | 0.32 | | -1.01 | | 20 | | 0.94 | | 1.05 | | 100-200 | | 400-500 | |
| 100-200 vs 500-600 | 0.006 | | -3.1 | | 20 | | 0.94 | | 1.25 | | 100-200 | | 500-600 | |
| 100-200 vs 600-700 | 0.04 | | -2.2 | | 20 | | 0.94 | | 1.19 | | 100-200 | | 600-700 | |
| 100-200 vs 700-800 | 0.004 | | -3.27 | | 20 | | 0.94 | | 1.22 | | 100-200 | | 700-800 | |
| 100-200 vs 800-900 | < 0.001 | | -7.2 | | 20 | | 0.94 | | 1.88 | | 100-200 | | 800-900 | |
| 100-200 vs 900-1000 | < 0.001 | | -7.88 | | 20 | | 0.94 | | 2.19 | | 100-200 | | 900-1000 | |
| 1000-1100 vs 1100-1200 | 0.48 | | -0.72 | | 20 | | 2.13 | | 2.25 | | 1000-1100 | | 1100-1200 | |
| 1000-1100 vs 1200-1300 | 0.63 | | -0.49 | | 20 | | 2.13 | | 2.23 | | 1000-1100 | | 1200-1300 | |
| 1000-1100 vs 200-300 | 0.002 | | 3.53 | | 20 | | 2.13 | | 1.43 | | 1000-1100 | | 200-300 | |
| 1000-1100 vs 300-400 | < 0.001 | | 6.42 | | 20 | | 2.13 | | 1.28 | | 1000-1100 | | 300-400 | |
| 1000-1100 vs 400-500 | < 0.001 | | 6.92 | | 20 | | 2.13 | | 1.05 | | 1000-1100 | | 400-500 | |
| 1000-1100 vs 500-600 | < 0.001 | | 5.81 | | 20 | | 2.13 | | 1.25 | | 1000-1100 | | 500-600 | |
| 1000-1100 vs 600-700 | < 0.001 | | 6.14 | | 20 | | 2.13 | | 1.19 | | 1000-1100 | | 600-700 | |
| 1000-1100 vs 700-800 | < 0.001 | | 6.33 | | 20 | | 2.13 | | 1.22 | | 1000-1100 | | 700-800 | |
| 1000-1100 vs 800-900 | 0.19 | | 1.36 | | 20 | | 2.13 | | 1.88 | | 1000-1100 | | 800-900 | |
| 1000-1100 vs 900-1000 | 0.68 | | -0.41 | | 20 | | 2.13 | | 2.19 | | 1000-1100 | | 900-1000 | |
| 1100-1200 vs 1200-1300 | 0.84 | | 0.21 | | 20 | | 2.25 | | 2.23 | | 1100-1200 | | 1200-1300 | |
| 1100-1200 vs 200-300 | 0.006 | | 3.05 | | 20 | | 2.25 | | 1.43 | | 1100-1200 | | 200-300 | |
| 1100-1200 vs 300-400 | 0.001 | | 4.13 | | 20 | | 2.25 | | 1.28 | | 1100-1200 | | 300-400 | |
| 1100-1200 vs 400-500 | < 0.001 | | 5.4 | | 20 | | 2.25 | | 1.05 | | 1100-1200 | | 400-500 | |
| 1100-1200 vs 500-600 | < 0.001 | | 4.75 | | 20 | | 2.25 | | 1.25 | | 1100-1200 | | 500-600 | |
| 1100-1200 vs 600-700 | < 0.001 | | 5.11 | | 20 | | 2.25 | | 1.19 | | 1100-1200 | | 600-700 | |
| 1100-1200 vs 700-800 | < 0.001 | | 4.45 | | 20 | | 2.25 | | 1.22 | | 1100-1200 | | 700-800 | |
| 1100-1200 vs 800-900 | 0.21 | | 1.31 | | 20 | | 2.25 | | 1.88 | | 1100-1200 | | 800-900 | |
| 1100-1200 vs 900-1000 | 0.83 | | 0.22 | | 20 | | 2.25 | | 2.19 | | 1100-1200 | | 900-1000 | |
| 1200-1300 vs 200-300 | 0.01 | | 2.84 | | 20 | | 2.23 | | 1.43 | | 1200-1300 | | 200-300 | |
| 1200-1300 vs 300-400 | 0.001 | | 3.84 | | 20 | | 2.23 | | 1.28 | | 1200-1300 | | 300-400 | |
| 1200-1300 vs 400-500 | < 0.001 | | 5.02 | | 20 | | 2.23 | | 1.05 | | 1200-1300 | | 400-500 | |
| 1200-1300 vs 500-600 | 0.001 | | 4.12 | | 20 | | 2.23 | | 1.25 | | 1200-1300 | | 500-600 | |
| 1200-1300 vs 600-700 | < 0.001 | | 4.16 | | 20 | | 2.23 | | 1.19 | | 1200-1300 | | 600-700 | |
| 1200-1300 vs 700-800 | 0.001 | | 3.88 | | 20 | | 2.23 | | 1.22 | | 1200-1300 | | 700-800 | |
| 1200-1300 vs 800-900 | 0.28 | | 1.11 | | 20 | | 2.23 | | 1.88 | | 1200-1300 | | 800-900 | |
| 1200-1300 vs 900-1000 | 0.9 | | 0.13 | | 20 | | 2.23 | | 2.19 | | 1200-1300 | | 900-1000 | |
| 200-300 vs 300-400 | 0.37 | | 0.92 | | 20 | | 1.43 | | 1.28 | | 200-300 | | 300-400 | |
| 200-300 vs 400-500 | 0.05 | | 2.08 | | 20 | | 1.43 | | 1.05 | | 200-300 | | 400-500 | |
| 200-300 vs 500-600 | 0.25 | | 1.19 | | 20 | | 1.43 | | 1.25 | | 200-300 | | 500-600 | |
| 200-300 vs 600-700 | 0.19 | | 1.36 | | 20 | | 1.43 | | 1.19 | | 200-300 | | 600-700 | |
| 200-300 vs 700-800 | 0.18 | | 1.4 | | 20 | | 1.43 | | 1.22 | | 200-300 | | 700-800 | |
| 200-300 vs 800-900 | 0.009 | | -2.91 | | 20 | | 1.43 | | 1.88 | | 200-300 | | 800-900 | |
| 200-300 vs 900-1000 | < 0.001 | | -5.24 | | 20 | | 1.43 | | 2.19 | | 200-300 | | 900-1000 | |
| 300-400 vs 400-500 | 0.021 | | 2.51 | | 20 | | 1.28 | | 1.05 | | 300-400 | | 400-500 | |
| 300-400 vs 500-600 | 0.84 | | 0.21 | | 20 | | 1.28 | | 1.25 | | 300-400 | | 500-600 | |
| 300-400 vs 600-700 | 0.52 | | 0.66 | | 20 | | 1.28 | | 1.19 | | 300-400 | | 600-700 | |
| 300-400 vs 700-800 | 0.53 | | 0.64 | | 20 | | 1.28 | | 1.22 | | 300-400 | | 700-800 | |
| 300-400 vs 800-900 | < 0.001 | | -4.48 | | 20 | | 1.28 | | 1.88 | | 300-400 | | 800-900 | |
| 300-400 vs 900-1000 | < 0.001 | | -6.25 | | 20 | | 1.28 | | 2.19 | | 300-400 | | 900-1000 | |
| 400-500 vs 500-600 | 0.055 | | -2.04 | | 20 | | 1.05 | | 1.25 | | 400-500 | | 500-600 | |
| 400-500 vs 600-700 | 0.27 | | -1.13 | | 20 | | 1.05 | | 1.19 | | 400-500 | | 600-700 | |
| 400-500 vs 700-800 | 0.074 | | -1.89 | | 20 | | 1.05 | | 1.22 | | 400-500 | | 700-800 | |
| 400-500 vs 800-900 | < 0.001 | | -4.77 | | 20 | | 1.05 | | 1.88 | | 400-500 | | 800-900 | |
| 400-500 vs 900-1000 | < 0.001 | | -6.79 | | 20 | | 1.05 | | 2.19 | | 400-500 | | 900-1000 | |
| 500-600 vs 600-700 | 0.38 | | 0.89 | | 20 | | 1.25 | | 1.19 | | 500-600 | | 600-700 | |
| 500-600 vs 700-800 | 0.65 | | 0.46 | | 20 | | 1.25 | | 1.22 | | 500-600 | | 700-800 | |
| 500-600 vs 800-900 | 0.001 | | -3.75 | | 20 | | 1.25 | | 1.88 | | 500-600 | | 800-900 | |
| 500-600 vs 900-1000 | < 0.001 | | -6.07 | | 20 | | 1.25 | | 2.19 | | 500-600 | | 900-1000 | |
| 600-700 vs 700-800 | 0.7 | | -0.4 | | 20 | | 1.19 | | 1.22 | | 600-700 | | 700-800 | |
| 600-700 vs 800-900 | < 0.001 | | -4.36 | | 20 | | 1.19 | | 1.88 | | 600-700 | | 800-900 | |
| 600-700 vs 900-1000 | < 0.001 | | -5.97 | | 20 | | 1.19 | | 2.19 | | 600-700 | | 900-1000 | |
| 700-800 vs 800-900 | < 0.001 | | -5.33 | | 20 | | 1.22 | | 1.88 | | 700-800 | | 800-900 | |
| 700-800 vs 900-1000 | < 0.001 | | -6.56 | | 20 | | 1.22 | | 2.19 | | 700-800 | | 900-1000 | |
| 800-900 vs 900-1000 | 0.026 | | -2.41 | | 20 | | 1.88 | | 2.19 | | 800-900 | | 900-1000 | |

## ANOVA - Right Angular Gyrus

**ANOVA**

| Effect | DFn | DFd | F | p | p<.05 | ges |
| --- | --- | --- | --- | --- | --- | --- |
| Response Type | 1 | 20 | 2.06 | 0.167 |  | 0.01 |
| Time Interval | 12 | 240 | 20.71 | < 0.001 | * | 0.31 |
| Response Type x Time Interval | 12 | 240 | 2.01 | 0.024 | * | < 0.01 |

**MAUCHLY’S TEST**

| Effect | W | p | p<.05 |  |  |  |
| --- | --- | --- | --- | --- | --- | --- |
| Time Interval | < 0.01 | < 0.001 | * |  |  |  |
| Response Type x Time Interval | < 0.01 | < 0.001 | * |  |  |  |

**SPHERICITY CORRECTIONS**

| Effect | W | p | p<.05 |  |  |  |
| --- | --- | --- | --- | --- | --- | --- |
| Effect | GGe | p[GG] | p[GG]<.05 | HFe | p[HF] | p[HF]<.05 |
| Time Interval | 0.31 | < 0.001 | * | 0.39 | < 0.001 | * |
| Response Type x Time Interval | 0.32 | 0.103 |  | 0.41 | 0.084 |  |

**POST-HOC Right Angular Gyrus - Time Interval**

| contrast | p.value | t.value | df | mean.1 | mean.2 | interval_1 | interval_2 |
| --- | --- | --- | --- | --- | --- | --- | --- |
| 0-100 vs 100-200 | < 0.001 | -6.6 | 20 | 0.27 | 0.97 | 0-100 | 100-200 |
| 0-100 vs 1000-1100 | < 0.001 | -7.46 | 20 | 0.27 | 1.97 | 0-100 | 1000-1100 |
| 0-100 vs 1100-1200 | < 0.001 | -7.53 | 20 | 0.27 | 2.05 | 0-100 | 1100-1200 |
| 0-100 vs 1200-1300 | < 0.001 | -9.17 | 20 | 0.27 | 2.25 | 0-100 | 1200-1300 |
| 0-100 vs 200-300 | < 0.001 | -7.16 | 20 | 0.27 | 1.63 | 0-100 | 200-300 |
| 0-100 vs 300-400 | < 0.001 | -7.3 | 20 | 0.27 | 1.36 | 0-100 | 300-400 |
| 0-100 vs 400-500 | < 0.001 | -7.25 | 20 | 0.27 | 1.07 | 0-100 | 400-500 |
| 0-100 vs 500-600 | < 0.001 | -7.76 | 20 | 0.27 | 1.1 | 0-100 | 500-600 |
| 0-100 vs 600-700 | < 0.001 | -6.56 | 20 | 0.27 | 1.04 | 0-100 | 600-700 |
| 0-100 vs 700-800 | < 0.001 | -5.55 | 20 | 0.27 | 1.03 | 0-100 | 700-800 |
| 0-100 vs 800-900 | < 0.001 | -10.08 | 20 | 0.27 | 1.66 | 0-100 | 800-900 |
| 0-100 vs 900-1000 | < 0.001 | -8.05 | 20 | 0.27 | 2.1 | 0-100 | 900-1000 |
| 100-200 vs 1000-1100 | 0.001 | -3.99 | 20 | 0.97 | 1.97 | 100-200 | 1000-1100 |
| 100-200 vs 1100-1200 | 0.001 | -4.29 | 20 | 0.97 | 2.05 | 100-200 | 1100-1200 |
| 100-200 vs 1200-1300 | < 0.001 | -5.68 | 20 | 0.97 | 2.25 | 100-200 | 1200-1300 |
| 100-200 vs 200-300 | 0.001 | -4.37 | 20 | 0.97 | 1.63 | 100-200 | 200-300 |
| 100-200 vs 300-400 | 0.003 | -3.71 | 20 | 0.97 | 1.36 | 100-200 | 300-400 |
| 100-200 vs 400-500 | 0.48 | -0.85 | 20 | 0.97 | 1.07 | 100-200 | 400-500 |
| 100-200 vs 500-600 | 0.48 | -0.84 | 20 | 0.97 | 1.1 | 100-200 | 500-600 |
| 100-200 vs 600-700 | 0.74 | -0.43 | 20 | 0.97 | 1.04 | 100-200 | 600-700 |
| 100-200 vs 700-800 | 0.78 | -0.36 | 20 | 0.97 | 1.03 | 100-200 | 700-800 |
| 100-200 vs 800-900 | < 0.001 | -4.71 | 20 | 0.97 | 1.66 | 100-200 | 800-900 |
| 100-200 vs 900-1000 | 0.001 | -4.4 | 20 | 0.97 | 2.1 | 100-200 | 900-1000 |
| 1000-1100 vs 1100-1200 | 0.6 | -0.63 | 20 | 1.97 | 2.05 | 1000-1100 | 1100-1200 |
| 1000-1100 vs 1200-1300 | 0.16 | -1.6 | 20 | 1.97 | 2.25 | 1000-1100 | 1200-1300 |
| 1000-1100 vs 200-300 | 0.29 | 1.24 | 20 | 1.97 | 1.63 | 1000-1100 | 200-300 |
| 1000-1100 vs 300-400 | 0.017 | 2.83 | 20 | 1.97 | 1.36 | 1000-1100 | 300-400 |
| 1000-1100 vs 400-500 | < 0.001 | 5.35 | 20 | 1.97 | 1.07 | 1000-1100 | 400-500 |
| 1000-1100 vs 500-600 | < 0.001 | 5.72 | 20 | 1.97 | 1.1 | 1000-1100 | 500-600 |
| 1000-1100 vs 600-700 | < 0.001 | 5.69 | 20 | 1.97 | 1.04 | 1000-1100 | 600-700 |
| 1000-1100 vs 700-800 | < 0.001 | 5.83 | 20 | 1.97 | 1.03 | 1000-1100 | 700-800 |
| 1000-1100 vs 800-900 | 0.097 | 1.92 | 20 | 1.97 | 1.66 | 1000-1100 | 800-900 |
| 1000-1100 vs 900-1000 | 0.48 | -0.85 | 20 | 1.97 | 2.1 | 1000-1100 | 900-1000 |
| 1100-1200 vs 1200-1300 | 0.07 | -2.12 | 20 | 2.05 | 2.25 | 1100-1200 | 1200-1300 |
| 1100-1200 vs 200-300 | 0.18 | 1.53 | 20 | 2.05 | 1.63 | 1100-1200 | 200-300 |
| 1100-1200 vs 300-400 | 0.014 | 2.93 | 20 | 2.05 | 1.36 | 1100-1200 | 300-400 |
| 1100-1200 vs 400-500 | < 0.001 | 4.97 | 20 | 2.05 | 1.07 | 1100-1200 | 400-500 |
| 1100-1200 vs 500-600 | < 0.001 | 5.71 | 20 | 2.05 | 1.1 | 1100-1200 | 500-600 |
| 1100-1200 vs 600-700 | < 0.001 | 5.32 | 20 | 2.05 | 1.04 | 1100-1200 | 600-700 |
| 1100-1200 vs 700-800 | < 0.001 | 5.19 | 20 | 2.05 | 1.03 | 1100-1200 | 700-800 |
| 1100-1200 vs 800-900 | 0.095 | 1.94 | 20 | 2.05 | 1.66 | 1100-1200 | 800-900 |
| 1100-1200 vs 900-1000 | 0.84 | -0.23 | 20 | 2.05 | 2.1 | 1100-1200 | 900-1000 |
| 1200-1300 vs 200-300 | 0.018 | 2.79 | 20 | 2.25 | 1.63 | 1200-1300 | 200-300 |
| 1200-1300 vs 300-400 | 0.001 | 4.02 | 20 | 2.25 | 1.36 | 1200-1300 | 300-400 |
| 1200-1300 vs 400-500 | < 0.001 | 5.94 | 20 | 2.25 | 1.07 | 1200-1300 | 400-500 |
| 1200-1300 vs 500-600 | < 0.001 | 6.57 | 20 | 2.25 | 1.1 | 1200-1300 | 500-600 |
| 1200-1300 vs 600-700 | < 0.001 | 5.98 | 20 | 2.25 | 1.04 | 1200-1300 | 600-700 |
| 1200-1300 vs 700-800 | < 0.001 | 5.79 | 20 | 2.25 | 1.03 | 1200-1300 | 700-800 |
| 1200-1300 vs 800-900 | 0.012 | 3.04 | 20 | 2.25 | 1.66 | 1200-1300 | 800-900 |
| 1200-1300 vs 900-1000 | 0.57 | 0.68 | 20 | 2.25 | 2.1 | 1200-1300 | 900-1000 |
| 200-300 vs 300-400 | 0.12 | 1.79 | 20 | 1.63 | 1.36 | 200-300 | 300-400 |
| 200-300 vs 400-500 | 0.014 | 2.93 | 20 | 1.63 | 1.07 | 200-300 | 400-500 |
| 200-300 vs 500-600 | 0.035 | 2.48 | 20 | 1.63 | 1.1 | 200-300 | 500-600 |
| 200-300 vs 600-700 | 0.036 | 2.46 | 20 | 1.63 | 1.04 | 200-300 | 600-700 |
| 200-300 vs 700-800 | 0.038 | 2.42 | 20 | 1.63 | 1.03 | 200-300 | 700-800 |
| 200-300 vs 800-900 | 0.88 | -0.15 | 20 | 1.63 | 1.66 | 200-300 | 800-900 |
| 200-300 vs 900-1000 | 0.094 | -1.95 | 20 | 1.63 | 2.1 | 200-300 | 900-1000 |
| 300-400 vs 400-500 | 0.017 | 2.84 | 20 | 1.36 | 1.07 | 300-400 | 400-500 |
| 300-400 vs 500-600 | 0.17 | 1.58 | 20 | 1.36 | 1.1 | 300-400 | 500-600 |
| 300-400 vs 600-700 | 0.13 | 1.74 | 20 | 1.36 | 1.04 | 300-400 | 600-700 |
| 300-400 vs 700-800 | 0.12 | 1.78 | 20 | 1.36 | 1.03 | 300-400 | 700-800 |
| 300-400 vs 800-900 | 0.088 | -2 | 20 | 1.36 | 1.66 | 300-400 | 800-900 |
| 300-400 vs 900-1000 | 0.008 | -3.2 | 20 | 1.36 | 2.1 | 300-400 | 900-1000 |
| 400-500 vs 500-600 | 0.81 | -0.3 | 20 | 1.07 | 1.1 | 400-500 | 500-600 |
| 400-500 vs 600-700 | 0.84 | 0.23 | 20 | 1.07 | 1.04 | 400-500 | 600-700 |
| 400-500 vs 700-800 | 0.81 | 0.3 | 20 | 1.07 | 1.03 | 400-500 | 700-800 |
| 400-500 vs 800-900 | < 0.001 | -6.13 | 20 | 1.07 | 1.66 | 400-500 | 800-900 |
| 400-500 vs 900-1000 | < 0.001 | -5.42 | 20 | 1.07 | 2.1 | 400-500 | 900-1000 |
| 500-600 vs 600-700 | 0.46 | 0.91 | 20 | 1.1 | 1.04 | 500-600 | 600-700 |
| 500-600 vs 700-800 | 0.53 | 0.75 | 20 | 1.1 | 1.03 | 500-600 | 700-800 |
| 500-600 vs 800-900 | < 0.001 | -5.19 | 20 | 1.1 | 1.66 | 500-600 | 800-900 |
| 500-600 vs 900-1000 | < 0.001 | -5.71 | 20 | 1.1 | 2.1 | 500-600 | 900-1000 |
| 600-700 vs 700-800 | 0.87 | 0.17 | 20 | 1.04 | 1.03 | 600-700 | 700-800 |
| 600-700 vs 800-900 | < 0.001 | -5.28 | 20 | 1.04 | 1.66 | 600-700 | 800-900 |
| 600-700 vs 900-1000 | < 0.001 | -6.03 | 20 | 1.04 | 2.1 | 600-700 | 900-1000 |
| 700-800 vs 800-900 | < 0.001 | -5.46 | 20 | 1.03 | 1.66 | 700-800 | 800-900 |
| 700-800 vs 900-1000 | < 0.001 | -6.13 | 20 | 1.03 | 2.1 | 700-800 | 900-1000 |
| 800-900 vs 900-1000 | 0.011 | -3.07 | 20 | 1.66 | 2.1 | 800-900 | 900-1000 |

## ANOVA - Left Supramarginal Gyrus

**ANOVA**

| Effect | DFn | DFd | F | p | p<.05 | ges |
| --- | --- | --- | --- | --- | --- | --- |
| Response Type | 1 | 20 | 5.83 | 0.025 | * | 0.01 |
| Time Interval | 12 | 240 | 33.73 | < 0.001 | * | 0.38 |
| Response Type x Time Interval | 12 | 240 | 2.79 | 0.001 | * | 0.01 |

**MAUCHLY’S TEST**

| Effect | W | p | p<.05 |  |  |  |
| --- | --- | --- | --- | --- | --- | --- |
| Time Interval | < 0.01 | < 0.001 | * |  |  |  |
| Response Type x Time Interval | < 0.01 | < 0.001 | * |  |  |  |

**SPHERICITY CORRECTIONS**

| Effect | GGe | p[GG] | p[GG]<.05 | HFe | p[HF] | p[HF]<.05 |
| --- | --- | --- | --- | --- | --- | --- |
| Time Interval | 0.29 | < 0.001 | * | 0.36 | < 0.001 | * |
| Response Type x Time Interval | 0.25 | 0.05 | * | 0.29 | 0.039 | * |

**POST-HOC Left Supramarginal Gyrus - Time Interval**

| contrast | p.value | t.value | df | mean.1 | mean.2 | interval_1 | interval_2 |
| --- | --- | --- | --- | --- | --- | --- | --- |
| 0-100 vs 100-200 | < 0.001 | -5.63 | 20 | 0.21 | 0.76 | 0-100 | 100-200 |
| 0-100 vs 1000-1100 | < 0.001 | -9.43 | 20 | 0.21 | 2.43 | 0-100 | 1000-1100 |
| 0-100 vs 1100-1200 | < 0.001 | -8.28 | 20 | 0.21 | 2.61 | 0-100 | 1100-1200 |
| 0-100 vs 1200-1300 | < 0.001 | -9.24 | 20 | 0.21 | 2.76 | 0-100 | 1200-1300 |
| 0-100 vs 200-300 | < 0.001 | -8.22 | 20 | 0.21 | 1.23 | 0-100 | 200-300 |
| 0-100 vs 300-400 | < 0.001 | -6.77 | 20 | 0.21 | 1.36 | 0-100 | 300-400 |
| 0-100 vs 400-500 | < 0.001 | -5.99 | 20 | 0.21 | 1.29 | 0-100 | 400-500 |
| 0-100 vs 500-600 | < 0.001 | -6.96 | 20 | 0.21 | 1.48 | 0-100 | 500-600 |
| 0-100 vs 600-700 | < 0.001 | -7.36 | 20 | 0.21 | 1.42 | 0-100 | 600-700 |
| 0-100 vs 700-800 | < 0.001 | -9.22 | 20 | 0.21 | 1.37 | 0-100 | 700-800 |
| 0-100 vs 800-900 | < 0.001 | -9.44 | 20 | 0.21 | 1.83 | 0-100 | 800-900 |
| 0-100 vs 900-1000 | < 0.001 | -11.65 | 20 | 0.21 | 2.26 | 0-100 | 900-1000 |
| 100-200 vs 1000-1100 | < 0.001 | -7.92 | 20 | 0.76 | 2.43 | 100-200 | 1000-1100 |
| 100-200 vs 1100-1200 | < 0.001 | -6.66 | 20 | 0.76 | 2.61 | 100-200 | 1100-1200 |
| 100-200 vs 1200-1300 | < 0.001 | -7.46 | 20 | 0.76 | 2.76 | 100-200 | 1200-1300 |
| 100-200 vs 200-300 | 0.001 | -3.97 | 20 | 0.76 | 1.23 | 100-200 | 200-300 |
| 100-200 vs 300-400 | < 0.001 | -4.84 | 20 | 0.76 | 1.36 | 100-200 | 300-400 |
| 100-200 vs 400-500 | 0.006 | -3.26 | 20 | 0.76 | 1.29 | 100-200 | 400-500 |
| 100-200 vs 500-600 | 0.001 | -4.21 | 20 | 0.76 | 1.48 | 100-200 | 500-600 |
| 100-200 vs 600-700 | 0.001 | -4.25 | 20 | 0.76 | 1.42 | 100-200 | 600-700 |
| 100-200 vs 700-800 | < 0.001 | -5.62 | 20 | 0.76 | 1.37 | 100-200 | 700-800 |
| 100-200 vs 800-900 | < 0.001 | -9.6 | 20 | 0.76 | 1.83 | 100-200 | 800-900 |
| 100-200 vs 900-1000 | < 0.001 | -10.57 | 20 | 0.76 | 2.26 | 100-200 | 900-1000 |
| 1000-1100 vs 1100-1200 | 0.25 | -1.31 | 20 | 2.43 | 2.61 | 1000-1100 | 1100-1200 |
| 1000-1100 vs 1200-1300 | 0.21 | -1.43 | 20 | 2.43 | 2.76 | 1000-1100 | 1200-1300 |
| 1000-1100 vs 200-300 | < 0.001 | 6.34 | 20 | 2.43 | 1.23 | 1000-1100 | 200-300 |
| 1000-1100 vs 300-400 | < 0.001 | 7.33 | 20 | 2.43 | 1.36 | 1000-1100 | 300-400 |
| 1000-1100 vs 400-500 | < 0.001 | 7.04 | 20 | 2.43 | 1.29 | 1000-1100 | 400-500 |
| 1000-1100 vs 500-600 | < 0.001 | 5.29 | 20 | 2.43 | 1.48 | 1000-1100 | 500-600 |
| 1000-1100 vs 600-700 | < 0.001 | 6.09 | 20 | 2.43 | 1.42 | 1000-1100 | 600-700 |
| 1000-1100 vs 700-800 | < 0.001 | 6.07 | 20 | 2.43 | 1.37 | 1000-1100 | 700-800 |
| 1000-1100 vs 800-900 | 0.006 | 3.25 | 20 | 2.43 | 1.83 | 1000-1100 | 800-900 |
| 1000-1100 vs 900-1000 | 0.26 | 1.28 | 20 | 2.43 | 2.26 | 1000-1100 | 900-1000 |
| 1100-1200 vs 1200-1300 | 0.38 | -0.98 | 20 | 2.61 | 2.76 | 1100-1200 | 1200-1300 |
| 1100-1200 vs 200-300 | < 0.001 | 5.44 | 20 | 2.61 | 1.23 | 1100-1200 | 200-300 |
| 1100-1200 vs 300-400 | < 0.001 | 5.53 | 20 | 2.61 | 1.36 | 1100-1200 | 300-400 |
| 1100-1200 vs 400-500 | < 0.001 | 6.62 | 20 | 2.61 | 1.29 | 1100-1200 | 400-500 |
| 1100-1200 vs 500-600 | < 0.001 | 5.91 | 20 | 2.61 | 1.48 | 1100-1200 | 500-600 |
| 1100-1200 vs 600-700 | < 0.001 | 6.29 | 20 | 2.61 | 1.42 | 1100-1200 | 600-700 |
| 1100-1200 vs 700-800 | < 0.001 | 5.56 | 20 | 2.61 | 1.37 | 1100-1200 | 700-800 |
| 1100-1200 vs 800-900 | 0.011 | 2.96 | 20 | 2.61 | 1.83 | 1100-1200 | 800-900 |
| 1100-1200 vs 900-1000 | 0.18 | 1.54 | 20 | 2.61 | 2.26 | 1100-1200 | 900-1000 |
| 1200-1300 vs 200-300 | < 0.001 | 6.06 | 20 | 2.76 | 1.23 | 1200-1300 | 200-300 |
| 1200-1300 vs 300-400 | < 0.001 | 5.23 | 20 | 2.76 | 1.36 | 1200-1300 | 300-400 |
| 1200-1300 vs 400-500 | < 0.001 | 6.57 | 20 | 2.76 | 1.29 | 1200-1300 | 400-500 |
| 1200-1300 vs 500-600 | < 0.001 | 6.37 | 20 | 2.76 | 1.48 | 1200-1300 | 500-600 |
| 1200-1300 vs 600-700 | < 0.001 | 6.49 | 20 | 2.76 | 1.42 | 1200-1300 | 600-700 |
| 1200-1300 vs 700-800 | < 0.001 | 6.11 | 20 | 2.76 | 1.37 | 1200-1300 | 700-800 |
| 1200-1300 vs 800-900 | 0.006 | 3.29 | 20 | 2.76 | 1.83 | 1200-1300 | 800-900 |
| 1200-1300 vs 900-1000 | 0.11 | 1.85 | 20 | 2.76 | 2.26 | 1200-1300 | 900-1000 |
| 200-300 vs 300-400 | 0.47 | -0.8 | 20 | 1.23 | 1.36 | 200-300 | 300-400 |
| 200-300 vs 400-500 | 0.8 | -0.27 | 20 | 1.23 | 1.29 | 200-300 | 400-500 |
| 200-300 vs 500-600 | 0.26 | -1.27 | 20 | 1.23 | 1.48 | 200-300 | 500-600 |
| 200-300 vs 600-700 | 0.35 | -1.06 | 20 | 1.23 | 1.42 | 200-300 | 600-700 |
| 200-300 vs 700-800 | 0.41 | -0.91 | 20 | 1.23 | 1.37 | 200-300 | 700-800 |
| 200-300 vs 800-900 | 0.002 | -3.78 | 20 | 1.23 | 1.83 | 200-300 | 800-900 |
| 200-300 vs 900-1000 | < 0.001 | -6.82 | 20 | 1.23 | 2.26 | 200-300 | 900-1000 |
| 300-400 vs 400-500 | 0.57 | 0.6 | 20 | 1.36 | 1.29 | 300-400 | 400-500 |
| 300-400 vs 500-600 | 0.52 | -0.72 | 20 | 1.36 | 1.48 | 300-400 | 500-600 |
| 300-400 vs 600-700 | 0.71 | -0.41 | 20 | 1.36 | 1.42 | 300-400 | 600-700 |
| 300-400 vs 700-800 | 0.97 | -0.04 | 20 | 1.36 | 1.37 | 300-400 | 700-800 |
| 300-400 vs 800-900 | 0.001 | -4.01 | 20 | 1.36 | 1.83 | 300-400 | 800-900 |
| 300-400 vs 900-1000 | < 0.001 | -7.04 | 20 | 1.36 | 2.26 | 300-400 | 900-1000 |
| 400-500 vs 500-600 | 0.039 | -2.36 | 20 | 1.29 | 1.48 | 400-500 | 500-600 |
| 400-500 vs 600-700 | 0.12 | -1.79 | 20 | 1.29 | 1.42 | 400-500 | 600-700 |
| 400-500 vs 700-800 | 0.53 | -0.69 | 20 | 1.29 | 1.37 | 400-500 | 700-800 |
| 400-500 vs 800-900 | 0.007 | -3.17 | 20 | 1.29 | 1.83 | 400-500 | 800-900 |
| 400-500 vs 900-1000 | < 0.001 | -6.27 | 20 | 1.29 | 2.26 | 400-500 | 900-1000 |
| 500-600 vs 600-700 | 0.38 | 1 | 20 | 1.48 | 1.42 | 500-600 | 600-700 |
| 500-600 vs 700-800 | 0.33 | 1.1 | 20 | 1.48 | 1.37 | 500-600 | 700-800 |
| 500-600 vs 800-900 | 0.096 | -1.91 | 20 | 1.48 | 1.83 | 500-600 | 800-900 |
| 500-600 vs 900-1000 | < 0.001 | -4.63 | 20 | 1.48 | 2.26 | 500-600 | 900-1000 |
| 600-700 vs 700-800 | 0.54 | 0.66 | 20 | 1.42 | 1.37 | 600-700 | 700-800 |
| 600-700 vs 800-900 | 0.02 | -2.69 | 20 | 1.42 | 1.83 | 600-700 | 800-900 |
| 600-700 vs 900-1000 | < 0.001 | -5.76 | 20 | 1.42 | 2.26 | 600-700 | 900-1000 |
| 700-800 vs 800-900 | 0.001 | -4.08 | 20 | 1.37 | 1.83 | 700-800 | 800-900 |
| 700-800 vs 900-1000 | < 0.001 | -7.41 | 20 | 1.37 | 2.26 | 700-800 | 900-1000 |
| 800-900 vs 900-1000 | 0.002 | -3.7 | 20 | 1.83 | 2.26 | 800-900 | 900-1000 |

## ANOVA - Right Supramarginal Gyrus

**ANOVA**

| Effect | DFn | DFd | F | p | p<.05 | ges |
| --- | --- | --- | --- | --- | --- | --- |
| Response Type | 1 | 20 | 1.56 | 0.226 |  | 0.01 |
| Interval | 12 | 240 | 26.17 | < 0.001 | * | 0.36 |
| Response Type x Time Interval | 12 | 240 | 4.07 | < 0.001 | * | 0.02 |

**MAUCHLY’S SPHERICITY TEST**

| Effect | W | p | p<.05 |  |  |  |
| --- | --- | --- | --- | --- | --- | --- |
| Time Interval | < 0.01 | < 0.001 | * |  |  |  |
| Response Type x Time Interval | < 0.01 | < 0.001 | * |  |  |  |

**SPHERICITY CORRECTIONS**

| Effect | GGe | p[GG] | p[GG]<.05 | HFe | p[HF] | p[HF]<.05 |
| --- | --- | --- | --- | --- | --- | --- |
| Time Interval | 0.37 | < 0.001 | * | 0.49 | < 0.001 | * |
| Response Type x Time Interval | 0.31 | 0.006 | * | 0.38 | 0.003 | * |

**POST-HOCS Right Supramarginal Gyrus – Response Type x Time Interval**

| contrast | p.value | t.value | df | mean.1 | mean.2 | Response Type_1 | Time Interval_1 | Response Type_2 | Time Interval_2 |
| --- | --- | --- | --- | --- | --- | --- | --- | --- | --- |
| Fast_0-100 vs Fast_100-200 | < 0.001 | -5.54 | 20 | 0.32 | 0.92 | Fast | 0-100 | Fast | 100-200 |
| Fast_0-100 vs Fast_1000-1100 | < 0.001 | -8.44 | 20 | 0.32 | 2.05 | Fast | 0-100 | Fast | 1000-1100 |
| Fast_0-100 vs Fast_1100-1200 | < 0.001 | -10.2 | 20 | 0.32 | 2.5 | Fast | 0-100 | Fast | 1100-1200 |
| Fast_0-100 vs Fast_1200-1300 | < 0.001 | -9.73 | 20 | 0.32 | 3.01 | Fast | 0-100 | Fast | 1200-1300 |
| Fast_0-100 vs Fast_200-300 | < 0.001 | -7.20 | 20 | 0.32 | 1.5 | Fast | 0-100 | Fast | 200-300 |
| Fast_0-100 vs Fast_300-400 | < 0.001 | -7.89 | 20 | 0.32 | 1.31 | Fast | 0-100 | Fast | 300-400 |
| Fast_0-100 vs Fast_400-500 | < 0.001 | -7.17 | 20 | 0.32 | 1.13 | Fast | 0-100 | Fast | 400-500 |
| Fast_0-100 vs Fast_500-600 | < 0.001 | -7.71 | 20 | 0.32 | 1.37 | Fast | 0-100 | Fast | 500-600 |
| Fast_0-100 vs Fast_600-700 | < 0.001 | -8.73 | 20 | 0.32 | 1.18 | Fast | 0-100 | Fast | 600-700 |
| Fast_0-100 vs Fast_700-800 | < 0.001 | -6.66 | 20 | 0.32 | 1.2 | Fast | 0-100 | Fast | 700-800 |
| Fast_0-100 vs Fast_800-900 | < 0.001 | -7.97 | 20 | 0.32 | 1.68 | Fast | 0-100 | Fast | 800-900 |
| Fast_0-100 vs Fast_900-1000 | < 0.001 | -8.2 | 20 | 0.32 | 1.72 | Fast | 0-100 | Fast | 900-1000 |
| Fast_0-100 vs Slow_0-100 | 0.66 | 0.52 | 20 | 0.32 | 0.28 | Fast | 0-100 | Slow | 0-100 |
| Fast_0-100 vs Slow_100-200 | < 0.001 | -4.7 | 20 | 0.32 | 0.94 | Fast | 0-100 | Slow | 100-200 |
| Fast_0-100 vs Slow_1000-1100 | < 0.001 | -6.48 | 20 | 0.32 | 1.85 | Fast | 0-100 | Slow | 1000-1100 |
| Fast_0-100 vs Slow_1100-1200 | < 0.001 | -8.38 | 20 | 0.32 | 2.11 | Fast | 0-100 | Slow | 1100-1200 |
| Fast_0-100 vs Slow_1200-1300 | < 0.001 | -9.62 | 20 | 0.32 | 2.31 | Fast | 0-100 | Slow | 1200-1300 |
| Fast_0-100 vs Slow_200-300 | < 0.001 | -8.38 | 20 | 0.32 | 1.45 | Fast | 0-100 | Slow | 200-300 |
| Fast_0-100 vs Slow_300-400 | < 0.001 | -5.07 | 20 | 0.32 | 1.26 | Fast | 0-100 | Slow | 300-400 |
| Fast_0-100 vs Slow_400-500 | < 0.001 | -5 | 20 | 0.32 | 1.15 | Fast | 0-100 | Slow | 400-500 |
| Fast_0-100 vs Slow_500-600 | < 0.001 | -6.08 | 20 | 0.32 | 1.36 | Fast | 0-100 | Slow | 500-600 |
| Fast_0-100 vs Slow_600-700 | < 0.001 | -5.78 | 20 | 0.32 | 1.09 | Fast | 0-100 | Slow | 600-700 |
| Fast_0-100 vs Slow_700-800 | < 0.001 | -4.75 | 20 | 0.32 | 1.1 | Fast | 0-100 | Slow | 700-800 |
| Fast_0-100 vs Slow_800-900 | < 0.001 | -6.32 | 20 | 0.32 | 1.62 | Fast | 0-100 | Slow | 800-900 |
| Fast_0-100 vs Slow_900-1000 | < 0.001 | -8.26 | 20 | 0.32 | 1.75 | Fast | 0-100 | Slow | 900-1000 |
| Fast_100-200 vs Fast_1000-1100 | < 0.001 | -4.89 | 20 | 0.92 | 2.05 | Fast | 100-200 | Fast | 1000-1100 |
| Fast_100-200 vs Fast_1100-1200 | < 0.001 | -6.34 | 20 | 0.92 | 2.5 | Fast | 100-200 | Fast | 1100-1200 |
| Fast_100-200 vs Fast_1200-1300 | < 0.001 | -6.92 | 20 | 0.92 | 3.01 | Fast | 100-200 | Fast | 1200-1300 |
| Fast_100-200 vs Fast_200-300 | 0.002 | -3.85 | 20 | 0.92 | 1.5 | Fast | 100-200 | Fast | 200-300 |
| Fast_100-200 vs Fast_300-400 | 0.002 | -3.85 | 20 | 0.92 | 1.31 | Fast | 100-200 | Fast | 300-400 |
| Fast_100-200 vs Fast_400-500 | 0.13 | -1.78 | 20 | 0.92 | 1.13 | Fast | 100-200 | Fast | 400-500 |
| Fast_100-200 vs Fast_500-600 | 0.011 | -3.12 | 20 | 0.92 | 1.37 | Fast | 100-200 | Fast | 500-600 |
| Fast_100-200 vs Fast_600-700 | 0.12 | -1.84 | 20 | 0.92 | 1.18 | Fast | 100-200 | Fast | 600-700 |
| Fast_100-200 vs Fast_700-800 | 0.16 | -1.65 | 20 | 0.92 | 1.2 | Fast | 100-200 | Fast | 700-800 |
| Fast_100-200 vs Fast_800-900 | 0.002 | -3.93 | 20 | 0.92 | 1.68 | Fast | 100-200 | Fast | 800-900 |
| Fast_100-200 vs Fast_900-1000 | 0.002 | -4.06 | 20 | 0.92 | 1.72 | Fast | 100-200 | Fast | 900-1000 |
| Fast_100-200 vs Slow_0-100 | < 0.001 | 5.6 | 20 | 0.92 | 0.28 | Fast | 100-200 | Slow | 0-100 |
| Fast_100-200 vs Slow_100-200 | 0.85 | -0.22 | 20 | 0.92 | 0.94 | Fast | 100-200 | Slow | 100-200 |
| Fast_100-200 vs Slow_1000-1100 | 0.002 | -3.89 | 20 | 0.92 | 1.85 | Fast | 100-200 | Slow | 1000-1100 |
| Fast_100-200 vs Slow_1100-1200 | < 0.001 | -5.29 | 20 | 0.92 | 2.11 | Fast | 100-200 | Slow | 1100-1200 |
| Fast_100-200 vs Slow_1200-1300 | < 0.001 | -6.1 | 20 | 0.92 | 2.31 | Fast | 100-200 | Slow | 1200-1300 |
| Fast_100-200 vs Slow_200-300 | 0.001 | -4.27 | 20 | 0.92 | 1.45 | Fast | 100-200 | Slow | 200-300 |
| Fast_100-200 vs Slow_300-400 | 0.093 | -1.98 | 20 | 0.92 | 1.26 | Fast | 100-200 | Slow | 300-400 |
| Fast_100-200 vs Slow_400-500 | 0.19 | -1.54 | 20 | 0.92 | 1.15 | Fast | 100-200 | Slow | 400-500 |
| Fast_100-200 vs Slow_500-600 | 0.02 | -2.8 | 20 | 0.92 | 1.36 | Fast | 100-200 | Slow | 500-600 |
| Fast_100-200 vs Slow_600-700 | 0.37 | -1.05 | 20 | 0.92 | 1.09 | Fast | 100-200 | Slow | 600-700 |
| Fast_100-200 vs Slow_700-800 | 0.45 | -0.9 | 20 | 0.92 | 1.1 | Fast | 100-200 | Slow | 700-800 |
| Fast_100-200 vs Slow_800-900 | 0.007 | -3.33 | 20 | 0.92 | 1.62 | Fast | 100-200 | Slow | 800-900 |
| Fast_100-200 vs Slow_900-1000 | 0.001 | -4.62 | 20 | 0.92 | 1.75 | Fast | 100-200 | Slow | 900-1000 |
| Fast_1000-1100 vs Fast_1100-1200 | 0.023 | -2.74 | 20 | 2.05 | 2.5 | Fast | 1000-1100 | Fast | 1100-1200 |
| Fast_1000-1100 vs Fast_1200-1300 | 0.007 | -3.3 | 20 | 2.05 | 3.01 | Fast | 1000-1100 | Fast | 1200-1300 |
| Fast_1000-1100 vs Fast_200-300 | 0.075 | 2.11 | 20 | 2.05 | 1.5 | Fast | 1000-1100 | Fast | 200-300 |
| Fast_1000-1100 vs Fast_300-400 | 0.001 | 4.32 | 20 | 2.05 | 1.31 | Fast | 1000-1100 | Fast | 300-400 |
| Fast_1000-1100 vs Fast_400-500 | < 0.001 | 5.14 | 20 | 2.05 | 1.13 | Fast | 1000-1100 | Fast | 400-500 |
| Fast_1000-1100 vs Fast_500-600 | 0.011 | 3.11 | 20 | 2.05 | 1.37 | Fast | 1000-1100 | Fast | 500-600 |
| Fast_1000-1100 vs Fast_600-700 | < 0.001 | 4.88 | 20 | 2.05 | 1.18 | Fast | 1000-1100 | Fast | 600-700 |
| Fast_1000-1100 vs Fast_700-800 | < 0.001 | 5.19 | 20 | 2.05 | 1.2 | Fast | 1000-1100 | Fast | 700-800 |
| Fast_1000-1100 vs Fast_800-900 | 0.026 | 2.65 | 20 | 2.05 | 1.68 | Fast | 1000-1100 | Fast | 800-900 |
| Fast_1000-1100 vs Fast_900-1000 | 0.016 | 2.91 | 20 | 2.05 | 1.72 | Fast | 1000-1100 | Fast | 900-1000 |
| Fast_1000-1100 vs Slow_0-100 | < 0.001 | 8.06 | 20 | 2.05 | 0.28 | Fast | 1000-1100 | Slow | 0-100 |
| Fast_1000-1100 vs Slow_100-200 | < 0.001 | 5.03 | 20 | 2.05 | 0.94 | Fast | 1000-1100 | Slow | 100-200 |
| Fast_1000-1100 vs Slow_1000-1100 | 0.28 | 1.26 | 20 | 2.05 | 1.85 | Fast | 1000-1100 | Slow | 1000-1100 |
| Fast_1000-1100 vs Slow_1100-1200 | 0.8 | -0.29 | 20 | 2.05 | 2.11 | Fast | 1000-1100 | Slow | 1100-1200 |
| Fast_1000-1100 vs Slow_1200-1300 | 0.27 | -1.29 | 20 | 2.05 | 2.31 | Fast | 1000-1100 | Slow | 1200-1300 |
| Fast_1000-1100 vs Slow_200-300 | 0.024 | 2.7 | 20 | 2.05 | 1.45 | Fast | 1000-1100 | Slow | 200-300 |
| Fast_1000-1100 vs Slow_300-400 | 0.001 | 4.17 | 20 | 2.05 | 1.26 | Fast | 1000-1100 | Slow | 300-400 |
| Fast_1000-1100 vs Slow_400-500 | 0.001 | 4.23 | 20 | 2.05 | 1.15 | Fast | 1000-1100 | Slow | 400-500 |
| Fast_1000-1100 vs Slow_500-600 | 0.019 | 2.82 | 20 | 2.05 | 1.36 | Fast | 1000-1100 | Slow | 500-600 |
| Fast_1000-1100 vs Slow_600-700 | < 0.001 | 4.85 | 20 | 2.05 | 1.09 | Fast | 1000-1100 | Slow | 600-700 |
| Fast_1000-1100 vs Slow_700-800 | < 0.001 | 4.81 | 20 | 2.05 | 1.1 | Fast | 1000-1100 | Slow | 700-800 |
| Fast_1000-1100 vs Slow_800-900 | 0.05 | 2.32 | 20 | 2.05 | 1.62 | Fast | 1000-1100 | Slow | 800-900 |
| Fast_1000-1100 vs Slow_900-1000 | 0.085 | 2.03 | 20 | 2.05 | 1.75 | Fast | 1000-1100 | Slow | 900-1000 |
| Fast_1100-1200 vs Fast_1200-1300 | 0.011 | -3.09 | 20 | 2.5 | 3.01 | Fast | 1100-1200 | Fast | 1200-1300 |
| Fast_1100-1200 vs Fast_200-300 | 0.003 | 3.68 | 20 | 2.5 | 1.5 | Fast | 1100-1200 | Fast | 200-300 |
| Fast_1100-1200 vs Fast_300-400 | < 0.001 | 5.33 | 20 | 2.5 | 1.31 | Fast | 1100-1200 | Fast | 300-400 |
| Fast_1100-1200 vs Fast_400-500 | < 0.001 | 6.55 | 20 | 2.5 | 1.13 | Fast | 1100-1200 | Fast | 400-500 |
| Fast_1100-1200 vs Fast_500-600 | < 0.001 | 5.01 | 20 | 2.5 | 1.37 | Fast | 1100-1200 | Fast | 500-600 |
| Fast_1100-1200 vs Fast_600-700 | < 0.001 | 6.22 | 20 | 2.5 | 1.18 | Fast | 1100-1200 | Fast | 600-700 |
| Fast_1100-1200 vs Fast_700-800 | < 0.001 | 6.23 | 20 | 2.5 | 1.2 | Fast | 1100-1200 | Fast | 700-800 |
| Fast_1100-1200 vs Fast_800-900 | 0.001 | 4.22 | 20 | 2.5 | 1.68 | Fast | 1100-1200 | Fast | 800-900 |
| Fast_1100-1200 vs Fast_900-1000 | 0.003 | 3.72 | 20 | 2.5 | 1.72 | Fast | 1100-1200 | Fast | 900-1000 |
| Fast_1100-1200 vs Slow_0-100 | < 0.001 | 9.53 | 20 | 2.5 | 0.28 | Fast | 1100-1200 | Slow | 0-100 |
| Fast_1100-1200 vs Slow_100-200 | < 0.001 | 6.77 | 20 | 2.5 | 0.94 | Fast | 1100-1200 | Slow | 100-200 |
| Fast_1100-1200 vs Slow_1000-1100 | 0.016 | 2.91 | 20 | 2.5 | 1.85 | Fast | 1100-1200 | Slow | 1000-1100 |
| Fast_1100-1200 vs Slow_1100-1200 | 0.039 | 2.46 | 20 | 2.5 | 2.11 | Fast | 1100-1200 | Slow | 1100-1200 |
| Fast_1100-1200 vs Slow_1200-1300 | 0.34 | 1.12 | 20 | 2.5 | 2.31 | Fast | 1100-1200 | Slow | 1200-1300 |
| Fast_1100-1200 vs Slow_200-300 | 0.001 | 4.54 | 20 | 2.5 | 1.45 | Fast | 1100-1200 | Slow | 200-300 |
| Fast_1100-1200 vs Slow_300-400 | < 0.001 | 5.19 | 20 | 2.5 | 1.26 | Fast | 1100-1200 | Slow | 300-400 |
| Fast_1100-1200 vs Slow_400-500 | < 0.001 | 5.74 | 20 | 2.5 | 1.15 | Fast | 1100-1200 | Slow | 400-500 |
| Fast_1100-1200 vs Slow_500-600 | 0.001 | 4.63 | 20 | 2.5 | 1.36 | Fast | 1100-1200 | Slow | 500-600 |
| Fast_1100-1200 vs Slow_600-700 | < 0.001 | 6.33 | 20 | 2.5 | 1.09 | Fast | 1100-1200 | Slow | 600-700 |
| Fast_1100-1200 vs Slow_700-800 | < 0.001 | 6.04 | 20 | 2.5 | 1.1 | Fast | 1100-1200 | Slow | 700-800 |
| Fast_1100-1200 vs Slow_800-900 | 0.001 | 4.34 | 20 | 2.5 | 1.62 | Fast | 1100-1200 | Slow | 800-900 |
| Fast_1100-1200 vs Slow_900-1000 | 0.005 | 3.5 | 20 | 2.5 | 1.75 | Fast | 1100-1200 | Slow | 900-1000 |
| Fast_1200-1300 vs Fast_200-300 | < 0.001 | 5.42 | 20 | 3.01 | 1.5 | Fast | 1200-1300 | Fast | 200-300 |
| Fast_1200-1300 vs Fast_300-400 | < 0.001 | 5.61 | 20 | 3.01 | 1.31 | Fast | 1200-1300 | Fast | 300-400 |
| Fast_1200-1300 vs Fast_400-500 | < 0.001 | 6.33 | 20 | 3.01 | 1.13 | Fast | 1200-1300 | Fast | 400-500 |
| Fast_1200-1300 vs Fast_500-600 | < 0.001 | 5.48 | 20 | 3.01 | 1.37 | Fast | 1200-1300 | Fast | 500-600 |
| Fast_1200-1300 vs Fast_600-700 | < 0.001 | 6.12 | 20 | 3.01 | 1.18 | Fast | 1200-1300 | Fast | 600-700 |
| Fast_1200-1300 vs Fast_700-800 | < 0.001 | 5.81 | 20 | 3.01 | 1.2 | Fast | 1200-1300 | Fast | 700-800 |
| Fast_1200-1300 vs Fast_800-900 | 0.001 | 4.41 | 20 | 3.01 | 1.68 | Fast | 1200-1300 | Fast | 800-900 |
| Fast_1200-1300 vs Fast_900-1000 | 0.001 | 4.24 | 20 | 3.01 | 1.72 | Fast | 1200-1300 | Fast | 900-1000 |
| Fast_1200-1300 vs Slow_0-100 | < 0.001 | 9.54 | 20 | 3.01 | 0.28 | Fast | 1200-1300 | Slow | 0-100 |
| Fast_1200-1300 vs Slow_100-200 | < 0.001 | 7.09 | 20 | 3.01 | 0.94 | Fast | 1200-1300 | Slow | 100-200 |
| Fast_1200-1300 vs Slow_1000-1100 | 0.006 | 3.44 | 20 | 3.01 | 1.85 | Fast | 1200-1300 | Slow | 1000-1100 |
| Fast_1200-1300 vs Slow_1100-1200 | 0.006 | 3.36 | 20 | 3.01 | 2.11 | Fast | 1200-1300 | Slow | 1100-1200 |
| Fast_1200-1300 vs Slow_1200-1300 | 0.023 | 2.72 | 20 | 3.01 | 2.31 | Fast | 1200-1300 | Slow | 1200-1300 |
| Fast_1200-1300 vs Slow_200-300 | < 0.001 | 5.75 | 20 | 3.01 | 1.45 | Fast | 1200-1300 | Slow | 200-300 |
| Fast_1200-1300 vs Slow_300-400 | < 0.001 | 5.56 | 20 | 3.01 | 1.26 | Fast | 1200-1300 | Slow | 300-400 |
| Fast_1200-1300 vs Slow_400-500 | < 0.001 | 5.67 | 20 | 3.01 | 1.15 | Fast | 1200-1300 | Slow | 400-500 |
| Fast_1200-1300 vs Slow_500-600 | < 0.001 | 5.01 | 20 | 3.01 | 1.36 | Fast | 1200-1300 | Slow | 500-600 |
| Fast_1200-1300 vs Slow_600-700 | < 0.001 | 6.05 | 20 | 3.01 | 1.09 | Fast | 1200-1300 | Slow | 600-700 |
| Fast_1200-1300 vs Slow_700-800 | < 0.001 | 5.73 | 20 | 3.01 | 1.1 | Fast | 1200-1300 | Slow | 700-800 |
| Fast_1200-1300 vs Slow_800-900 | < 0.001 | 4.69 | 20 | 3.01 | 1.62 | Fast | 1200-1300 | Slow | 800-900 |
| Fast_1200-1300 vs Slow_900-1000 | 0.002 | 4.04 | 20 | 3.01 | 1.75 | Fast | 1200-1300 | Slow | 900-1000 |
| Fast_200-300 vs Fast_300-400 | 0.32 | 1.16 | 20 | 1.5 | 1.31 | Fast | 200-300 | Fast | 300-400 |
| Fast_200-300 vs Fast_400-500 | 0.11 | 1.87 | 20 | 1.5 | 1.13 | Fast | 200-300 | Fast | 400-500 |
| Fast_200-300 vs Fast_500-600 | 0.6 | 0.62 | 20 | 1.5 | 1.37 | Fast | 200-300 | Fast | 500-600 |
| Fast_200-300 vs Fast_600-700 | 0.21 | 1.48 | 20 | 1.5 | 1.18 | Fast | 200-300 | Fast | 600-700 |
| Fast_200-300 vs Fast_700-800 | 0.28 | 1.26 | 20 | 1.5 | 1.2 | Fast | 200-300 | Fast | 700-800 |
| Fast_200-300 vs Fast_800-900 | 0.57 | -0.66 | 20 | 1.5 | 1.68 | Fast | 200-300 | Fast | 800-900 |
| Fast_200-300 vs Fast_900-1000 | 0.41 | -0.98 | 20 | 1.5 | 1.72 | Fast | 200-300 | Fast | 900-1000 |
| Fast_200-300 vs Slow_0-100 | < 0.001 | 6.94 | 20 | 1.5 | 0.28 | Fast | 200-300 | Slow | 0-100 |
| Fast_200-300 vs Slow_100-200 | 0.012 | 3.05 | 20 | 1.5 | 0.94 | Fast | 200-300 | Slow | 100-200 |
| Fast_200-300 vs Slow_1000-1100 | 0.34 | -1.13 | 20 | 1.5 | 1.85 | Fast | 200-300 | Slow | 1000-1100 |
| Fast_200-300 vs Slow_1100-1200 | 0.081 | -2.06 | 20 | 1.5 | 2.11 | Fast | 200-300 | Slow | 1100-1200 |
| Fast_200-300 vs Slow_1200-1300 | 0.016 | -2.91 | 20 | 1.5 | 2.31 | Fast | 200-300 | Slow | 1200-1300 |
| Fast_200-300 vs Slow_200-300 | 0.65 | 0.54 | 20 | 1.5 | 1.45 | Fast | 200-300 | Slow | 200-300 |
| Fast_200-300 vs Slow_300-400 | 0.36 | 1.08 | 20 | 1.5 | 1.26 | Fast | 200-300 | Slow | 300-400 |
| Fast_200-300 vs Slow_400-500 | 0.22 | 1.43 | 20 | 1.5 | 1.15 | Fast | 200-300 | Slow | 400-500 |
| Fast_200-300 vs Slow_500-600 | 0.62 | 0.6 | 20 | 1.5 | 1.36 | Fast | 200-300 | Slow | 500-600 |
| Fast_200-300 vs Slow_600-700 | 0.14 | 1.72 | 20 | 1.5 | 1.09 | Fast | 200-300 | Slow | 600-700 |
| Fast_200-300 vs Slow_700-800 | 0.2 | 1.51 | 20 | 1.5 | 1.1 | Fast | 200-300 | Slow | 700-800 |
| Fast_200-300 vs Slow_800-900 | 0.74 | -0.38 | 20 | 1.5 | 1.62 | Fast | 200-300 | Slow | 800-900 |
| Fast_200-300 vs Slow_900-1000 | 0.38 | -1.02 | 20 | 1.5 | 1.75 | Fast | 200-300 | Slow | 900-1000 |
| Fast_300-400 vs Fast_400-500 | 0.14 | 1.74 | 20 | 1.31 | 1.13 | Fast | 300-400 | Fast | 400-500 |
| Fast_300-400 vs Fast_500-600 | 0.74 | -0.38 | 20 | 1.31 | 1.37 | Fast | 300-400 | Fast | 500-600 |
| Fast_300-400 vs Fast_600-700 | 0.46 | 0.88 | 20 | 1.31 | 1.18 | Fast | 300-400 | Fast | 600-700 |
| Fast_300-400 vs Fast_700-800 | 0.53 | 0.74 | 20 | 1.31 | 1.2 | Fast | 300-400 | Fast | 700-800 |
| Fast_300-400 vs Fast_800-900 | 0.047 | -2.35 | 20 | 1.31 | 1.68 | Fast | 300-400 | Fast | 800-900 |
| Fast_300-400 vs Fast_900-1000 | 0.02 | -2.8 | 20 | 1.31 | 1.72 | Fast | 300-400 | Fast | 900-1000 |
| Fast_300-400 vs Slow_0-100 | < 0.001 | 7.81 | 20 | 1.31 | 0.28 | Fast | 300-400 | Slow | 0-100 |
| Fast_300-400 vs Slow_100-200 | 0.01 | 3.15 | 20 | 1.31 | 0.94 | Fast | 300-400 | Slow | 100-200 |
| Fast_300-400 vs Slow_1000-1100 | 0.013 | -3.02 | 20 | 1.31 | 1.85 | Fast | 300-400 | Slow | 1000-1100 |
| Fast_300-400 vs Slow_1100-1200 | 0.002 | -4.01 | 20 | 1.31 | 2.11 | Fast | 300-400 | Slow | 1100-1200 |
| Fast_300-400 vs Slow_1200-1300 | < 0.001 | -4.71 | 20 | 1.31 | 2.31 | Fast | 300-400 | Slow | 1200-1300 |
| Fast_300-400 vs Slow_200-300 | 0.32 | -1.17 | 20 | 1.31 | 1.45 | Fast | 300-400 | Slow | 200-300 |
| Fast_300-400 vs Slow_300-400 | 0.72 | 0.41 | 20 | 1.31 | 1.26 | Fast | 300-400 | Slow | 300-400 |
| Fast_300-400 vs Slow_400-500 | 0.34 | 1.11 | 20 | 1.31 | 1.15 | Fast | 300-400 | Slow | 400-500 |
| Fast_300-400 vs Slow_500-600 | 0.78 | -0.32 | 20 | 1.31 | 1.36 | Fast | 300-400 | Slow | 500-600 |
| Fast_300-400 vs Slow_600-700 | 0.23 | 1.41 | 20 | 1.31 | 1.09 | Fast | 300-400 | Slow | 600-700 |
| Fast_300-400 vs Slow_700-800 | 0.31 | 1.18 | 20 | 1.31 | 1.1 | Fast | 300-400 | Slow | 700-800 |
| Fast_300-400 vs Slow_800-900 | 0.15 | -1.7 | 20 | 1.31 | 1.62 | Fast | 300-400 | Slow | 800-900 |
| Fast_300-400 vs Slow_900-1000 | 0.005 | -3.46 | 20 | 1.31 | 1.75 | Fast | 300-400 | Slow | 900-1000 |
| Fast_400-500 vs Fast_500-600 | 0.027 | -2.64 | 20 | 1.13 | 1.37 | Fast | 400-500 | Fast | 500-600 |
| Fast_400-500 vs Fast_600-700 | 0.64 | -0.56 | 20 | 1.13 | 1.18 | Fast | 400-500 | Fast | 600-700 |
| Fast_400-500 vs Fast_700-800 | 0.57 | -0.68 | 20 | 1.13 | 1.2 | Fast | 400-500 | Fast | 700-800 |
| Fast_400-500 vs Fast_800-900 | 0.004 | -3.58 | 20 | 1.13 | 1.68 | Fast | 400-500 | Fast | 800-900 |
| Fast_400-500 vs Fast_900-1000 | 0.002 | -3.93 | 20 | 1.13 | 1.72 | Fast | 400-500 | Fast | 900-1000 |
| Fast_400-500 vs Slow_0-100 | < 0.001 | 5.56 | 20 | 1.13 | 0.28 | Fast | 400-500 | Slow | 0-100 |
| Fast_400-500 vs Slow_100-200 | 0.29 | 1.24 | 20 | 1.13 | 0.94 | Fast | 400-500 | Slow | 100-200 |
| Fast_400-500 vs Slow_1000-1100 | 0.004 | -3.58 | 20 | 1.13 | 1.85 | Fast | 400-500 | Slow | 1000-1100 |
| Fast_400-500 vs Slow_1100-1200 | < 0.001 | -5.04 | 20 | 1.13 | 2.11 | Fast | 400-500 | Slow | 1100-1200 |
| Fast_400-500 vs Slow_1200-1300 | < 0.001 | -5.73 | 20 | 1.13 | 2.31 | Fast | 400-500 | Slow | 1200-1300 |
| Fast_400-500 vs Slow_200-300 | 0.092 | -1.99 | 20 | 1.13 | 1.45 | Fast | 400-500 | Slow | 200-300 |
| Fast_400-500 vs Slow_300-400 | 0.52 | -0.76 | 20 | 1.13 | 1.26 | Fast | 400-500 | Slow | 300-400 |
| Fast_400-500 vs Slow_400-500 | 0.85 | -0.21 | 20 | 1.13 | 1.15 | Fast | 400-500 | Slow | 400-500 |
| Fast_400-500 vs Slow_500-600 | 0.18 | -1.58 | 20 | 1.13 | 1.36 | Fast | 400-500 | Slow | 500-600 |
| Fast_400-500 vs Slow_600-700 | 0.81 | 0.27 | 20 | 1.13 | 1.09 | Fast | 400-500 | Slow | 600-700 |
| Fast_400-500 vs Slow_700-800 | 0.86 | 0.19 | 20 | 1.13 | 1.1 | Fast | 400-500 | Slow | 700-800 |
| Fast_400-500 vs Slow_800-900 | 0.024 | -2.69 | 20 | 1.13 | 1.62 | Fast | 400-500 | Slow | 800-900 |
| Fast_400-500 vs Slow_900-1000 | < 0.001 | -4.92 | 20 | 1.13 | 1.75 | Fast | 400-500 | Slow | 900-1000 |
| Fast_500-600 vs Fast_600-700 | 0.11 | 1.9 | 20 | 1.37 | 1.18 | Fast | 500-600 | Fast | 600-700 |
| Fast_500-600 vs Fast_700-800 | 0.23 | 1.4 | 20 | 1.37 | 1.2 | Fast | 500-600 | Fast | 700-800 |
| Fast_500-600 vs Fast_800-900 | 0.13 | -1.79 | 20 | 1.37 | 1.68 | Fast | 500-600 | Fast | 800-900 |
| Fast_500-600 vs Fast_900-1000 | 0.092 | -1.99 | 20 | 1.37 | 1.72 | Fast | 500-600 | Fast | 900-1000 |
| Fast_500-600 vs Slow_0-100 | < 0.001 | 5.95 | 20 | 1.37 | 0.28 | Fast | 500-600 | Slow | 0-100 |
| Fast_500-600 vs Slow_100-200 | 0.05 | 2.32 | 20 | 1.37 | 0.94 | Fast | 500-600 | Slow | 100-200 |
| Fast_500-600 vs Slow_1000-1100 | 0.095 | -1.97 | 20 | 1.37 | 1.85 | Fast | 500-600 | Slow | 1000-1100 |
| Fast_500-600 vs Slow_1100-1200 | 0.008 | -3.26 | 20 | 1.37 | 2.11 | Fast | 500-600 | Slow | 1100-1200 |
| Fast_500-600 vs Slow_1200-1300 | 0.002 | -4.04 | 20 | 1.37 | 2.31 | Fast | 500-600 | Slow | 1200-1300 |
| Fast_500-600 vs Slow_200-300 | 0.71 | -0.43 | 20 | 1.37 | 1.45 | Fast | 500-600 | Slow | 200-300 |
| Fast_500-600 vs Slow_300-400 | 0.7 | 0.45 | 20 | 1.37 | 1.26 | Fast | 500-600 | Slow | 300-400 |
| Fast_500-600 vs Slow_400-500 | 0.29 | 1.22 | 20 | 1.37 | 1.15 | Fast | 500-600 | Slow | 400-500 |
| Fast_500-600 vs Slow_500-600 | 0.96 | 0.04 | 20 | 1.37 | 1.36 | Fast | 500-600 | Slow | 500-600 |
| Fast_500-600 vs Slow_600-700 | 0.12 | 1.81 | 20 | 1.37 | 1.09 | Fast | 500-600 | Slow | 600-700 |
| Fast_500-600 vs Slow_700-800 | 0.17 | 1.62 | 20 | 1.37 | 1.1 | Fast | 500-600 | Slow | 700-800 |
| Fast_500-600 vs Slow_800-900 | 0.28 | -1.26 | 20 | 1.37 | 1.62 | Fast | 500-600 | Slow | 800-900 |
| Fast_500-600 vs Slow_900-1000 | 0.046 | -2.36 | 20 | 1.37 | 1.75 | Fast | 500-600 | Slow | 900-1000 |
| Fast_600-700 vs Fast_700-800 | 0.82 | -0.26 | 20 | 1.18 | 1.2 | Fast | 600-700 | Fast | 700-800 |
| Fast_600-700 vs Fast_800-900 | 0.003 | -3.77 | 20 | 1.18 | 1.68 | Fast | 600-700 | Fast | 800-900 |
| Fast_600-700 vs Fast_900-1000 | 0.002 | -3.87 | 20 | 1.18 | 1.72 | Fast | 600-700 | Fast | 900-1000 |
| Fast_600-700 vs Slow_0-100 | < 0.001 | 5.93 | 20 | 1.18 | 0.28 | Fast | 600-700 | Slow | 0-100 |
| Fast_600-700 vs Slow_100-200 | 0.23 | 1.4 | 20 | 1.18 | 0.94 | Fast | 600-700 | Slow | 100-200 |
| Fast_600-700 vs Slow_1000-1100 | 0.012 | -3.04 | 20 | 1.18 | 1.85 | Fast | 600-700 | Slow | 1000-1100 |
| Fast_600-700 vs Slow_1100-1200 | 0.001 | -4.37 | 20 | 1.18 | 2.11 | Fast | 600-700 | Slow | 1100-1200 |
| Fast_600-700 vs Slow_1200-1300 | < 0.001 | -5.13 | 20 | 1.18 | 2.31 | Fast | 600-700 | Slow | 1200-1300 |
| Fast_600-700 vs Slow_200-300 | 0.22 | -1.43 | 20 | 1.18 | 1.45 | Fast | 600-700 | Slow | 200-300 |
| Fast_600-700 vs Slow_300-400 | 0.74 | -0.39 | 20 | 1.18 | 1.26 | Fast | 600-700 | Slow | 300-400 |
| Fast_600-700 vs Slow_400-500 | 0.88 | 0.16 | 20 | 1.18 | 1.15 | Fast | 600-700 | Slow | 400-500 |
| Fast_600-700 vs Slow_500-600 | 0.36 | -1.08 | 20 | 1.18 | 1.36 | Fast | 600-700 | Slow | 500-600 |
| Fast_600-700 vs Slow_600-700 | 0.54 | 0.73 | 20 | 1.18 | 1.09 | Fast | 600-700 | Slow | 600-700 |
| Fast_600-700 vs Slow_700-800 | 0.62 | 0.59 | 20 | 1.18 | 1.1 | Fast | 600-700 | Slow | 700-800 |
| Fast_600-700 vs Slow_800-900 | 0.045 | -2.38 | 20 | 1.18 | 1.62 | Fast | 600-700 | Slow | 800-900 |
| Fast_600-700 vs Slow_900-1000 | 0.003 | -3.72 | 20 | 1.18 | 1.75 | Fast | 600-700 | Slow | 900-1000 |
| Fast_700-800 vs Fast_800-900 | 0.003 | -3.75 | 20 | 1.2 | 1.68 | Fast | 700-800 | Fast | 800-900 |
| Fast_700-800 vs Fast_900-1000 | 0.002 | -3.89 | 20 | 1.2 | 1.72 | Fast | 700-800 | Fast | 900-1000 |
| Fast_700-800 vs Slow_0-100 | < 0.001 | 5.16 | 20 | 1.2 | 0.28 | Fast | 700-800 | Slow | 0-100 |
| Fast_700-800 vs Slow_100-200 | 0.25 | 1.34 | 20 | 1.2 | 0.94 | Fast | 700-800 | Slow | 100-200 |
| Fast_700-800 vs Slow_1000-1100 | 0.009 | -3.2 | 20 | 1.2 | 1.85 | Fast | 700-800 | Slow | 1000-1100 |
| Fast_700-800 vs Slow_1100-1200 | 0.001 | -4.34 | 20 | 1.2 | 2.11 | Fast | 700-800 | Slow | 1100-1200 |
| Fast_700-800 vs Slow_1200-1300 | < 0.001 | -5.14 | 20 | 1.2 | 2.31 | Fast | 700-800 | Slow | 1200-1300 |
| Fast_700-800 vs Slow_200-300 | 0.29 | -1.23 | 20 | 1.2 | 1.45 | Fast | 700-800 | Slow | 200-300 |
| Fast_700-800 vs Slow_300-400 | 0.78 | -0.32 | 20 | 1.2 | 1.26 | Fast | 700-800 | Slow | 300-400 |
| Fast_700-800 vs Slow_400-500 | 0.82 | 0.26 | 20 | 1.2 | 1.15 | Fast | 700-800 | Slow | 400-500 |
| Fast_700-800 vs Slow_500-600 | 0.43 | -0.93 | 20 | 1.2 | 1.36 | Fast | 700-800 | Slow | 500-600 |
| Fast_700-800 vs Slow_600-700 | 0.51 | 0.78 | 20 | 1.2 | 1.09 | Fast | 700-800 | Slow | 600-700 |
| Fast_700-800 vs Slow_700-800 | 0.52 | 0.75 | 20 | 1.2 | 1.1 | Fast | 700-800 | Slow | 700-800 |
| Fast_700-800 vs Slow_800-900 | 0.05 | -2.31 | 20 | 1.2 | 1.62 | Fast | 700-800 | Slow | 800-900 |
| Fast_700-800 vs Slow_900-1000 | 0.004 | -3.66 | 20 | 1.2 | 1.75 | Fast | 700-800 | Slow | 900-1000 |
| Fast_800-900 vs Fast_900-1000 | 0.72 | -0.41 | 20 | 1.68 | 1.72 | Fast | 800-900 | Fast | 900-1000 |
| Fast_800-900 vs Slow_0-100 | < 0.001 | 7.07 | 20 | 1.68 | 0.28 | Fast | 800-900 | Slow | 0-100 |
| Fast_800-900 vs Slow_100-200 | 0.003 | 3.71 | 20 | 1.68 | 0.94 | Fast | 800-900 | Slow | 100-200 |
| Fast_800-900 vs Slow_1000-1100 | 0.47 | -0.86 | 20 | 1.68 | 1.85 | Fast | 800-900 | Slow | 1000-1100 |
| Fast_800-900 vs Slow_1100-1200 | 0.062 | -2.21 | 20 | 1.68 | 2.11 | Fast | 800-900 | Slow | 1100-1200 |
| Fast_800-900 vs Slow_1200-1300 | 0.011 | -3.09 | 20 | 1.68 | 2.31 | Fast | 800-900 | Slow | 1200-1300 |
| Fast_800-900 vs Slow_200-300 | 0.38 | 1.03 | 20 | 1.68 | 1.45 | Fast | 800-900 | Slow | 200-300 |
| Fast_800-900 vs Slow_300-400 | 0.078 | 2.09 | 20 | 1.68 | 1.26 | Fast | 800-900 | Slow | 300-400 |
| Fast_800-900 vs Slow_400-500 | 0.021 | 2.78 | 20 | 1.68 | 1.15 | Fast | 800-900 | Slow | 400-500 |
| Fast_800-900 vs Slow_500-600 | 0.19 | 1.55 | 20 | 1.68 | 1.36 | Fast | 800-900 | Slow | 500-600 |
| Fast_800-900 vs Slow_600-700 | 0.006 | 3.4 | 20 | 1.68 | 1.09 | Fast | 800-900 | Slow | 600-700 |
| Fast_800-900 vs Slow_700-800 | 0.005 | 3.47 | 20 | 1.68 | 1.1 | Fast | 800-900 | Slow | 700-800 |
| Fast_800-900 vs Slow_800-900 | 0.67 | 0.51 | 20 | 1.68 | 1.62 | Fast | 800-900 | Slow | 800-900 |
| Fast_800-900 vs Slow_900-1000 | 0.68 | -0.49 | 20 | 1.68 | 1.75 | Fast | 800-900 | Slow | 900-1000 |
| Fast_900-1000 vs Slow_0-100 | < 0.001 | 6.95 | 20 | 1.72 | 0.28 | Fast | 900-1000 | Slow | 0-100 |
| Fast_900-1000 vs Slow_100-200 | 0.003 | 3.67 | 20 | 1.72 | 0.94 | Fast | 900-1000 | Slow | 100-200 |
| Fast_900-1000 vs Slow_1000-1100 | 0.61 | -0.61 | 20 | 1.72 | 1.85 | Fast | 900-1000 | Slow | 1000-1100 |
| Fast_900-1000 vs Slow_1100-1200 | 0.15 | -1.68 | 20 | 1.72 | 2.11 | Fast | 900-1000 | Slow | 1100-1200 |
| Fast_900-1000 vs Slow_1200-1300 | 0.037 | -2.48 | 20 | 1.72 | 2.31 | Fast | 900-1000 | Slow | 1200-1300 |
| Fast_900-1000 vs Slow_200-300 | 0.26 | 1.32 | 20 | 1.72 | 1.45 | Fast | 900-1000 | Slow | 200-300 |
| Fast_900-1000 vs Slow_300-400 | 0.052 | 2.3 | 20 | 1.72 | 1.26 | Fast | 900-1000 | Slow | 300-400 |
| Fast_900-1000 vs Slow_400-500 | 0.023 | 2.72 | 20 | 1.72 | 1.15 | Fast | 900-1000 | Slow | 400-500 |
| Fast_900-1000 vs Slow_500-600 | 0.18 | 1.59 | 20 | 1.72 | 1.36 | Fast | 900-1000 | Slow | 500-600 |
| Fast_900-1000 vs Slow_600-700 | 0.006 | 3.39 | 20 | 1.72 | 1.09 | Fast | 900-1000 | Slow | 600-700 |
| Fast_900-1000 vs Slow_700-800 | 0.006 | 3.36 | 20 | 1.72 | 1.1 | Fast | 900-1000 | Slow | 700-800 |
| Fast_900-1000 vs Slow_800-900 | 0.64 | 0.56 | 20 | 1.72 | 1.62 | Fast | 900-1000 | Slow | 800-900 |
| Fast_900-1000 vs Slow_900-1000 | 0.85 | -0.2 | 20 | 1.72 | 1.75 | Fast | 900-1000 | Slow | 900-1000 |
| Slow_0-100 vs Slow_100-200 | < 0.001 | -7.41 | 20 | 0.28 | 0.94 | Slow | 0-100 | Slow | 100-200 |
| Slow_0-100 vs Slow_1000-1100 | < 0.001 | -6.87 | 20 | 0.28 | 1.85 | Slow | 0-100 | Slow | 1000-1100 |
| Slow_0-100 vs Slow_1100-1200 | < 0.001 | -8.71 | 20 | 0.28 | 2.11 | Slow | 0-100 | Slow | 1100-1200 |
| Slow_0-100 vs Slow_1200-1300 | < 0.001 | -10.16 | 20 | 0.28 | 2.31 | Slow | 0-100 | Slow | 1200-1300 |
| Slow_0-100 vs Slow_200-300 | < 0.001 | -9.52 | 20 | 0.28 | 1.45 | Slow | 0-100 | Slow | 200-300 |
| Slow_0-100 vs Slow_300-400 | < 0.001 | -6.56 | 20 | 0.28 | 1.26 | Slow | 0-100 | Slow | 300-400 |
| Slow_0-100 vs Slow_400-500 | < 0.001 | -5.24 | 20 | 0.28 | 1.15 | Slow | 0-100 | Slow | 400-500 |
| Slow_0-100 vs Slow_500-600 | < 0.001 | -5.9 | 20 | 0.28 | 1.36 | Slow | 0-100 | Slow | 500-600 |
| Slow_0-100 vs Slow_600-700 | < 0.001 | -5.64 | 20 | 0.28 | 1.09 | Slow | 0-100 | Slow | 600-700 |
| Slow_0-100 vs Slow_700-800 | < 0.001 | -4.64 | 20 | 0.28 | 1.1 | Slow | 0-100 | Slow | 700-800 |
| Slow_0-100 vs Slow_800-900 | < 0.001 | -6.69 | 20 | 0.28 | 1.62 | Slow | 0-100 | Slow | 800-900 |
| Slow_0-100 vs Slow_900-1000 | < 0.001 | -8.01 | 20 | 0.28 | 1.75 | Slow | 0-100 | Slow | 900-1000 |
| Slow_100-200 vs Slow_1000-1100 | 0.001 | -4.23 | 20 | 0.94 | 1.85 | Slow | 100-200 | Slow | 1000-1100 |
| Slow_100-200 vs Slow_1100-1200 | < 0.001 | -5.87 | 20 | 0.94 | 2.11 | Slow | 100-200 | Slow | 1100-1200 |
| Slow_100-200 vs Slow_1200-1300 | < 0.001 | -6.8 | 20 | 0.94 | 2.31 | Slow | 100-200 | Slow | 1200-1300 |
| Slow_100-200 vs Slow_200-300 | 0.001 | -4.11 | 20 | 0.94 | 1.45 | Slow | 100-200 | Slow | 200-300 |
| Slow_100-200 vs Slow_300-400 | 0.045 | -2.38 | 20 | 0.94 | 1.26 | Slow | 100-200 | Slow | 300-400 |
| Slow_100-200 vs Slow_400-500 | 0.19 | -1.55 | 20 | 0.94 | 1.15 | Slow | 100-200 | Slow | 400-500 |
| Slow_100-200 vs Slow_500-600 | 0.03 | -2.59 | 20 | 0.94 | 1.36 | Slow | 100-200 | Slow | 500-600 |
| Slow_100-200 vs Slow_600-700 | 0.4 | -0.99 | 20 | 0.94 | 1.09 | Slow | 100-200 | Slow | 600-700 |
| Slow_100-200 vs Slow_700-800 | 0.5 | -0.8 | 20 | 0.94 | 1.1 | Slow | 100-200 | Slow | 700-800 |
| Slow_100-200 vs Slow_800-900 | 0.004 | -3.54 | 20 | 0.94 | 1.62 | Slow | 100-200 | Slow | 800-900 |
| Slow_100-200 vs Slow_900-1000 | 0.001 | -4.53 | 20 | 0.94 | 1.75 | Slow | 100-200 | Slow | 900-1000 |
| Slow_1000-1100 vs Slow_1100-1200 | 0.1 | -1.94 | 20 | 1.85 | 2.11 | Slow | 1000-1100 | Slow | 1100-1200 |
| Slow_1000-1100 vs Slow_1200-1300 | 0.03 | -2.59 | 20 | 1.85 | 2.31 | Slow | 1000-1100 | Slow | 1200-1300 |
| Slow_1000-1100 vs Slow_200-300 | 0.16 | 1.64 | 20 | 1.85 | 1.45 | Slow | 1000-1100 | Slow | 200-300 |
| Slow_1000-1100 vs Slow_300-400 | 0.004 | 3.65 | 20 | 1.85 | 1.26 | Slow | 1000-1100 | Slow | 300-400 |
| Slow_1000-1100 vs Slow_400-500 | 0.001 | 4.14 | 20 | 1.85 | 1.15 | Slow | 1000-1100 | Slow | 400-500 |
| Slow_1000-1100 vs Slow_500-600 | 0.047 | 2.36 | 20 | 1.85 | 1.36 | Slow | 1000-1100 | Slow | 500-600 |
| Slow_1000-1100 vs Slow_600-700 | < 0.001 | 4.63 | 20 | 1.85 | 1.09 | Slow | 1000-1100 | Slow | 600-700 |
| Slow_1000-1100 vs Slow_700-800 | 0.001 | 4.56 | 20 | 1.85 | 1.1 | Slow | 1000-1100 | Slow | 700-800 |
| Slow_1000-1100 vs Slow_800-900 | 0.25 | 1.34 | 20 | 1.85 | 1.62 | Slow | 1000-1100 | Slow | 800-900 |
| Slow_1000-1100 vs Slow_900-1000 | 0.5 | 0.79 | 20 | 1.85 | 1.75 | Slow | 1000-1100 | Slow | 900-1000 |
| Slow_1100-1200 vs Slow_1200-1300 | 0.025 | -2.69 | 20 | 2.11 | 2.31 | Slow | 1100-1200 | Slow | 1200-1300 |
| Slow_1100-1200 vs Slow_200-300 | 0.021 | 2.77 | 20 | 2.11 | 1.45 | Slow | 1100-1200 | Slow | 200-300 |
| Slow_1100-1200 vs Slow_300-400 | 0.001 | 4.29 | 20 | 2.11 | 1.26 | Slow | 1100-1200 | Slow | 300-400 |
| Slow_1100-1200 vs Slow_400-500 | < 0.001 | 6.24 | 20 | 2.11 | 1.15 | Slow | 1100-1200 | Slow | 400-500 |
| Slow_1100-1200 vs Slow_500-600 | 0.002 | 4.09 | 20 | 2.11 | 1.36 | Slow | 1100-1200 | Slow | 500-600 |
| Slow_1100-1200 vs Slow_600-700 | < 0.001 | 5.96 | 20 | 2.11 | 1.09 | Slow | 1100-1200 | Slow | 600-700 |
| Slow_1100-1200 vs Slow_700-800 | < 0.001 | 5.97 | 20 | 2.11 | 1.1 | Slow | 1100-1200 | Slow | 700-800 |
| Slow_1100-1200 vs Slow_800-900 | 0.005 | 3.46 | 20 | 2.11 | 1.62 | Slow | 1100-1200 | Slow | 800-900 |
| Slow_1100-1200 vs Slow_900-1000 | 0.081 | 2.06 | 20 | 2.11 | 1.75 | Slow | 1100-1200 | Slow | 900-1000 |
| Slow_1200-1300 vs Slow_200-300 | 0.003 | 3.82 | 20 | 2.31 | 1.45 | Slow | 1200-1300 | Slow | 200-300 |
| Slow_1200-1300 vs Slow_300-400 | < 0.001 | 5.03 | 20 | 2.31 | 1.26 | Slow | 1200-1300 | Slow | 300-400 |
| Slow_1200-1300 vs Slow_400-500 | < 0.001 | 6.94 | 20 | 2.31 | 1.15 | Slow | 1200-1300 | Slow | 400-500 |
| Slow_1200-1300 vs Slow_500-600 | < 0.001 | 5.2 | 20 | 2.31 | 1.36 | Slow | 1200-1300 | Slow | 500-600 |
| Slow_1200-1300 vs Slow_600-700 | < 0.001 | 6.96 | 20 | 2.31 | 1.09 | Slow | 1200-1300 | Slow | 600-700 |
| Slow_1200-1300 vs Slow_700-800 | < 0.001 | 7.3 | 20 | 2.31 | 1.1 | Slow | 1200-1300 | Slow | 700-800 |
| Slow_1200-1300 vs Slow_800-900 | < 0.001 | 4.68 | 20 | 2.31 | 1.62 | Slow | 1200-1300 | Slow | 800-900 |
| Slow_1200-1300 vs Slow_900-1000 | 0.016 | 2.91 | 20 | 2.31 | 1.75 | Slow | 1200-1300 | Slow | 900-1000 |
| Slow_200-300 vs Slow_300-400 | 0.29 | 1.23 | 20 | 1.45 | 1.26 | Slow | 200-300 | Slow | 300-400 |
| Slow_200-300 vs Slow_400-500 | 0.18 | 1.58 | 20 | 1.45 | 1.15 | Slow | 200-300 | Slow | 400-500 |
| Slow_200-300 vs Slow_500-600 | 0.7 | 0.46 | 20 | 1.45 | 1.36 | Slow | 200-300 | Slow | 500-600 |
| Slow_200-300 vs Slow_600-700 | 0.098 | 1.95 | 20 | 1.45 | 1.09 | Slow | 200-300 | Slow | 600-700 |
| Slow_200-300 vs Slow_700-800 | 0.17 | 1.61 | 20 | 1.45 | 1.1 | Slow | 200-300 | Slow | 700-800 |
| Slow_200-300 vs Slow_800-900 | 0.54 | -0.72 | 20 | 1.45 | 1.62 | Slow | 200-300 | Slow | 800-900 |
| Slow_200-300 vs Slow_900-1000 | 0.18 | -1.59 | 20 | 1.45 | 1.75 | Slow | 200-300 | Slow | 900-1000 |
| Slow_300-400 vs Slow_400-500 | 0.57 | 0.68 | 20 | 1.26 | 1.15 | Slow | 300-400 | Slow | 400-500 |
| Slow_300-400 vs Slow_500-600 | 0.69 | -0.47 | 20 | 1.26 | 1.36 | Slow | 300-400 | Slow | 500-600 |
| Slow_300-400 vs Slow_600-700 | 0.42 | 0.95 | 20 | 1.26 | 1.09 | Slow | 300-400 | Slow | 600-700 |
| Slow_300-400 vs Slow_700-800 | 0.48 | 0.83 | 20 | 1.26 | 1.1 | Slow | 300-400 | Slow | 700-800 |
| Slow_300-400 vs Slow_800-900 | 0.1 | -1.93 | 20 | 1.26 | 1.62 | Slow | 300-400 | Slow | 800-900 |
| Slow_300-400 vs Slow_900-1000 | 0.01 | -3.15 | 20 | 1.26 | 1.75 | Slow | 300-400 | Slow | 900-1000 |
| Slow_400-500 vs Slow_500-600 | 0.093 | -1.98 | 20 | 1.15 | 1.36 | Slow | 400-500 | Slow | 500-600 |
| Slow_400-500 vs Slow_600-700 | 0.65 | 0.54 | 20 | 1.15 | 1.09 | Slow | 400-500 | Slow | 600-700 |
| Slow_400-500 vs Slow_700-800 | 0.74 | 0.38 | 20 | 1.15 | 1.1 | Slow | 400-500 | Slow | 700-800 |
| Slow_400-500 vs Slow_800-900 | 0.014 | -2.99 | 20 | 1.15 | 1.62 | Slow | 400-500 | Slow | 800-900 |
| Slow_400-500 vs Slow_900-1000 | 0.001 | -4.25 | 20 | 1.15 | 1.75 | Slow | 400-500 | Slow | 900-1000 |
| Slow_500-600 vs Slow_600-700 | 0.04 | 2.44 | 20 | 1.36 | 1.09 | Slow | 500-600 | Slow | 600-700 |
| Slow_500-600 vs Slow_700-800 | 0.079 | 2.08 | 20 | 1.36 | 1.1 | Slow | 500-600 | Slow | 700-800 |
| Slow_500-600 vs Slow_800-900 | 0.19 | -1.53 | 20 | 1.36 | 1.62 | Slow | 500-600 | Slow | 800-900 |
| Slow_500-600 vs Slow_900-1000 | 0.042 | -2.42 | 20 | 1.36 | 1.75 | Slow | 500-600 | Slow | 900-1000 |
| Slow_600-700 vs Slow_700-800 | 0.94 | -0.09 | 20 | 1.09 | 1.1 | Slow | 600-700 | Slow | 700-800 |
| Slow_600-700 vs Slow_800-900 | 0.008 | -3.26 | 20 | 1.09 | 1.62 | Slow | 600-700 | Slow | 800-900 |
| Slow_600-700 vs Slow_900-1000 | < 0.001 | -5.41 | 20 | 1.09 | 1.75 | Slow | 600-700 | Slow | 900-1000 |
| Slow_700-800 vs Slow_800-900 | 0.004 | -3.58 | 20 | 1.1 | 1.62 | Slow | 700-800 | Slow | 800-900 |
| Slow_700-800 vs Slow_900-1000 | < 0.001 | -5.05 | 20 | 1.1 | 1.75 | Slow | 700-800 | Slow | 900-1000 |
| Slow_800-900 vs Slow_900-1000 | 0.45 | -0.89 | 20 | 1.62 | 1.75 | Slow | 800-900 | Slow | 900-1000 |

## Corrected P-values for ANOVAs across all ROI

The following table reports the p-values from the ANOVA in the preceding tables after a correction with FDR method.

The FDR corrected p-values (last column) correspond to those reported in the main manuscript.

| ROI | Effect | DFn | DFd | F | p | p<.05 | p.corrGG | sig.GG | p FDR |
| --- | --- | --- | --- | --- | --- | --- | --- | --- | --- |
| G_pariet_inf.Angular.L | Response Type | 1 | 20 | 9.74 | 0.03 | * |  |  | 0.03 |
| G_pariet_inf.Angular.L | Time Interval | 12 | 240 | 23.55 | <0.001 | * | <0.001 | * | <0.001 |
| G_pariet_inf.Angular.L | Response Type  x Time Interval | 12 | 240 | 2.44 | 0.07 | * | 0.07 |  | 0.15 |
| G_pariet_inf.Angular.R | Time Interval | 12 | 240 | 20.71 | <0.001 | * | <0.001 | * | <0.001 |
| G_pariet_inf.Angular.R | Response Type  x Time Interval | 12 | 240 | 2.01 | 0.10 | * | 0.10 |  | 0.15 |
| G_pariet_inf.Supramar.L | Response Type | 1 | 20 | 5.83 | 0.03 | * |  |  | 0.10 |
| G_pariet_inf.Supramar.L | Time Interval | 12 | 240 | 33.73 | <0.001 | * | <0.001 | * | <0.001 |
| G_pariet_inf.Supramar.L | Response Type  x Time Interval | 12 | 240 | 2.79 | 0.05 | * | 0.05 | * | 0.15 |
| G_pariet_inf.Supramar.R | Time Interval | 12 | 240 | 26.17 | <0.001 | * | <0.001 | * | <0.001 |
| G_pariet_inf.Supramar.R | Response Type x Time Interval | 12 | 240 | 4.07 | 0.01 | * | 0.001 | * | 0.03 |

# Details on additional ANOVA

This section contains all the effects and post-hocs related to the additional ANOVAs on the data of ERF on ROI. This ANOVA, uses type 1 Sum of Squares due to small number of observations in relation to number of levels (which prevented us to use the more appropriate type 3 ANOVA) included also the ROI as factor with for levels (Left Angular Gyrus, Right Angular Gyrus, Left Supramarginal Gyrus, Right Supramarginal Gyrus). All reported p-values were corrected according to the FDR method. In the case of significant interactions in the ANOVA, only the post-hocs for the higher order interactions are reported.

Analysis of this ANOVA confirms the analysis reported in the manuscript, but they should be taken with caution as the effects are calculated sequentially.

ANOVA with ROI as factor

| Effect | DFn | DFd | F | p | P<05 | ges |
| --- | --- | --- | --- | --- | --- | --- |
| Response Type | 1 | 20 1 | 10.33 | 0.004 | * | 0.02 |
| Time Interval | 12 | 2 40 | 5.52 | <0.001 | * | 0.41 |
| ROI | 3 | 60 | 0.712 | 0.55 |  | 0.01 |
| Response Type x Time Interval | 12 | 240 | 4.75 | <0.001 | * | 0.01 |
| Responst Type x ROI | 3 | 60 | 0.96 | 0.41 |  | 0.002 |
| Time Interval x ROI | 36 | 720 | 2.05 | 0.0003 | * | 0.03 |
| Response Type x Time Interval x ROI | 36 1 | 720 | 1.49 | 0.03 | * | 0.001 |

## POST-HOCS for Additional ANOVAs

Given the very large number of post-hoc associated with the interaction (5356), the data can be retrieved from the OSF repository from this permalink <https://osf.io/b32xy/> , in the form of .Rdata file. This file can be handled within the R environment.

# References

Benjamini, Y., & Hochberg, Y. (1995). Controlling the False Discovery Rate: A Practical And Powerful Approach to Mu. *Journal of the Royal Statistical Society. Series B (Methodological)*, *57*(1), 289–300. Retrieved from http://www.jstor.org/stable/2346101

Tadel, F., Baillet, S., Mosher, J. C., Pantazis, D., & Leahy, R. M. (2011). Brainstorm: A user-friendly application for MEG/EEG analysis. *Computational Intelligence and Neuroscience*, *2011*. http://doi.org/10.1155/2011/879716
